# Small extracellular vesicles orchestrated pathological communications between breast cancer cells and cardiomyocytes as a novel mechanism exacerbating anthracycline cardiotoxicity by fueling ferroptosis

**DOI:** 10.1016/j.redox.2025.103843

**Published:** 2025-09-03

**Authors:** Dong Han, Tianhu Wang, Xiaoyao Li, Cheng Qin, Yingjie Zhang, Tingwen Zhou, Shan Gao, Weiwei Zhang, Yongjun Wang, Yan Ma, Feng Cao

**Affiliations:** a National Clinical Research Center for Geriatric Diseases, The Second Medical Center, Chinese PLA General Hospital, 100853, Beijing, China; b Arrhythmia Center, State Key Laboratory of Cardiovascular Disease, Fuwai Hospital, National Center for Cardiovascular Diseases, Chinese Academy of Medical Sciences and Peking Union Medical College, Beijing, China; c Department of Cardiovascular Surgery, Union Hospital, Tongji Medical College, Huazhong University of Science and Technology, 1277# Jiefang Avenue, Wuhan, Hubei, 430022, China; d Institute of Geriatric Medicine, The Second Medical Center, Chinese PLA General Hospital, 100853, Beijing, China

**Keywords:** Cardio-oncology, Extracellular vesicles, Cardiotoxicity, Doxorubicin, microRNA

## Abstract

Small extracellular vesicles (sEVs) critically orchestrate inter-tissue and inter-organ communications and may play essential roles in heart-tumor interaction. However, whether cancer-secreted sEVs affect the progression of doxorubicin-induced cardiotoxicity (DOXIC) *via* orchestrating the tumor cell-cardiomyocyte crosstalk has not yet been explored. Herein, we reveal that Doxorubicin (DOX)-treated breast cancer cells secrete sEVs (D-BCC-sEVs) that exacerbate DOX-induced ferroptosis of human iPSC-derived cardiomyocytes (hiCMs). miRNA expression profiling and experimental validations reveal that miR-338-3p is upregulated in D-BCC-sEVs and mediate its detrimental effects. Incubation of hiCMs with D-BCC-sEVs or overexpression of miR-338-3p alone intensifies DOX-induced ferroptosis. N^6^-methyladenosine (m^6^A) is revealed to mediate the upregulation of miR-338-3p in D-BCCs. D-BCCs-enriched miR-338-3p is packaged in sEVs and transferred into hiCMs in a RBMX-dependent manner, miR-338-3p further targets anti-ferroptotic genes CP, SLC7A11, and GPX4 to facilitate their degradation. Therapeutically, dual-functional decoying sEVs encapsulated with miR-338-3p inhibitor mitigate DOXIC in an orthotopic breast cancer mouse model. Clinically, plasma sEVs isolated from patients experiencing DOXIC enhance DOX-induced ferroptosis in hiCM, which is rescued by miR-338-3p inhibitor. Our findings uncovered for the first time that DOX-treated BCCs exacerbated DOXIC through releasing pro-ferroptotic miR-338-3p-enriched sEVs. Therefore, targeting sEVs-mediated tumor/cardiomyocyte pathological communication may offer a novel approach for the management of DOXIC.

## Graphical abstract

**Figure:**
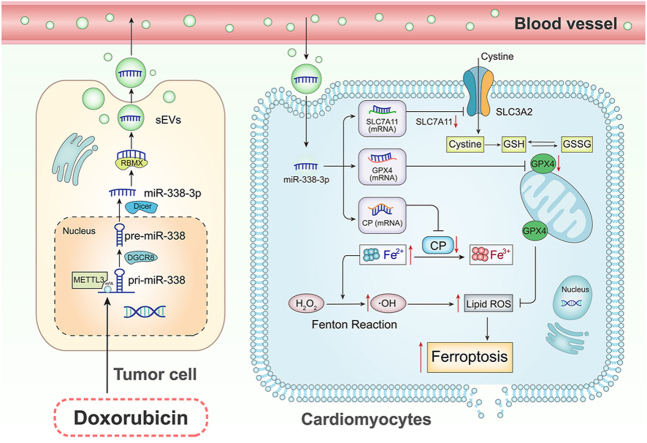

**Table:** Nonstandard Abbreviations and Acronyms

Abbreviations	Full name
CP	Ceruloplasmin
D-BCC-sEVs	doxorubicin-induced breast cancer-cell-secreted sEVs
DGCR8	DiGeorge syndrome critical region 8
DOX	Doxorubicin
DOXIC	DOX-induced cardiotoxicity
D-sEVs	Small extracellular vesicles derived from DOX-induced cadiomyopathy patients
E/A	The ratio of the peak early transmitral flow velocity to the peak late transmitral flow velocity
E/E′	the ratio of the peak early transmitral flow velocity to the peak early diastolic mitral annular velocity
ELAVL1	ELAV like RNA binding protein 1
EMSA	Electrophoretic mobility shift assay
GPX4	Glutathione Peroxidase 4
H&E	Hematoxylin and eosin
hiCMs	Human induced pluripotent stem cell-derived cardiomyocytes
LVEF	Left ventricular ejection fraction
LVES	Left ventricular fractional shortening
MT	Mutant
N-BCC-sEVs	Small extracellular vesicles derived from normal control breast cancer cells
NC inh	Negative control inhibitor
N-sEVs	Small extracellular vesicles derived from normal healthy control
N^6^-methyladenosine	m^6^A
NTA	Nanoparticle tracking analysis
RBMX	RNA Binding Motif Protein X-Linked
RBP	RNA binding protein
sEVs	Small extracellular vesicles
SLC7A11	Solute Carrier Family 7 Member 11
TDN	Tetrahedral DNA
TfR1	Transferrin receptor protein 1
3′-UTR	3′-untranslated regions
WGA	Wheat germ agglutinin
WT	Wild type
231 cells	MDA-MB-231 cells
338 inh	miR-338-3p inhibitor
338 mim	miR-338-3p mimic

## 1 Introduction

Recent years have witnessed a huge progress in new therapies for cancer, which resulted in a remarkable improvement in the chances of surviving a diagnosis of cancer, with the 5-year survival rate for early-stage breast cancer increasing from 79 % in 1990 to 88 % in 2012 and 90 % in 2017 [1,2]. However, these survived cancer patients were increasingly inflicted with cardiovascular events secondary to the malignant process itself or its treatment [3]. According to a comprehensive review of breast cancer survivors in the United States, the risk of death from cardiovascular disease was shown to exceed the risk of death from the initial malignancy or recurring disease *per se* [4]. Among the various anti-tumor agents inducing adverse cardiovascular events in cancer survivors, anthracyclines remain the best recognized and mostly frequently used chemotherapeutic drugs in various solid and hematologic tumors [5]. However, the optimal therapeutic strategies to avoid or lessen doxorubicin-induced cardiotoxicity (DOXIC) are still being worked out, and the management of this cardiotoxicity is still a hotly researched topic.

Recent studies have proposed a bidirectional interaction between cancer and cardiovascular disease (CVD) [6]. On one hand, cardiovascular disease can serve as risk factors for cancer. Patients with heart failure (HF) possessed a 1.68-fold greater chance of developing cancer, even after adjustment for body mass index, smoking, and co-morbidities [7]. Several recent preclinical studies have further shown that the existence of cardiovascular diseases can also directly drive tumor growth. In the context of myocardial infarction (MI)-induced HF, the formation of tumors in tumor-prone C57BL/6-Apc^Min^ mice accelerated dramatically [8]. Similarly, plasma extracted from mice with aortic constriction promoted tumor cell growth [9]. On the other hand, cancer can also serve as risk factors for cardiovascular disease. In comparison with healthy controls, the risk of CVD (including HF) was significantly elevated in survivors of multiple myeloma [incidence rate ratio (IRR) 1.7], lung cancer (IRR 1.58) and breast cancer (IRR 1.13) [10]. In animal models, the existence of cancer promoted cardiac atrophy and subsequently deteriorated cardiac function [11]. The progression of breast cancer in spontaneous breast cancer mice correlated with subclinical left ventricular dysfunction, which can be partly ascribed to defective cardiomyocyte Ca^2+^ handling [12]. However, whether cancer and heart interacted in the context of DOXIC and whether this interaction further alters the course of DOXIC is unknown.

Small extracellular vesicles (sEVs) have emerged as a novel messaging system of the organism, mediating cell-cell and interorgan communication *via* transporting bioactive proteins, mRNAs, and microRNA (miRNAs) [13]. Previous studies have demonstrated that sEVs play a crucial role in the interorgan communication between heart and other remote organs/tissues [14]. Dysfunctional adipocyte-derived sEVs exacerbated myocardial ischemia/reperfusion injury (MI/RI) in the diabetic heart *via* delivering the destructive miR-130b-3p into the heart [15]. Reciprocally, myocardial infarction altered the cardiomyocyte-adipocyte communication *via* sEVs-transmitted miR-23-27-24 cluster derived from injured cardiomyocytes, thereby impairing adipose tissue function [16]. Likewise, sEVs secreted by brown adipose tissue contribute to exercise-afforded cardioprotection *via* delivering the cardioprotective miRNAs into the heart [17]. In addition to sEVs-mediated heart-adipose tissue crosstalk, there are also many other studies reporting sEV-mediated communication between heart and other organs such as heart-skeletal muscle crosstalk [18], heart-kidney crosstalk [19], heart-bone marrow crosstalk [20]. Interestingly, a recent study hinted a potential sEVs-mediated inter-organ communication between myeloma and heart. sEVs isolated from the serum of multiple myeloma patients contain circ-G042080 that was positively correlated with multiple myoloma-related myocardial damage markers and could induce myocardial injury [21]. Of note, a recently published study documented that sEVs containing miR-22-3p secreted from cardiomyocytes blunted the sensitivity of osteosarcoma cells to erastin-induced ferroptosis in ischemic heart failure [22]. However, if and how sEVs participated in the possible interorgan communication between cancer and heart in the context of DOXIC remains unknown.

In the present study, the effects of breast cancer-derived sEVs on doxorubicin-induced cardiac injury was evaluated through *in vivo* proof of concept experiments and *in vitro* investigations dissecting the molecular mechanisms. The International Society for Extracellular Vesicles (ISEV) discourages the use of biogenesis-based terms such as exosomes unless such an EV population is specifically separated and characterized [23]. Therefore, we used the term “Small Extracellular Vesicles (sEVs)” in the present study for scientific rigor. The results show that doxorubicin-induced breast cancer-cell-secreted sEVs (D-BCC-sEVs) aggravate anthracycline (doxorubicin) cardiotoxicity through transmitting miR-338-3p to incite ferroptosis in hiCMs. Targeting abnormal BC-sEVs releasing or miR-338-3p-mediated pathologic communication between injured breast cancer cells and cardiomyocytes may be a novel strategy to block additional doxorubicin toxicity to heart. These results may provide novel insights into the pathogenesis of tumor and cardiovascular comorbidities.

## 2 Materials and methods

### 2.1 Data availability statement

The miRNA sequencing data are available in Gene Expression Omnibus under GSE192763.

Data, analysis methods, and study materials will be made available to other researchers for the purposes of reproducing the results or replicating the procedure on request to the corresponding authors. Additional methods details are available in the Supplemental Material.

### 2.2 Animals

The female C57BL/6J wild-type mice (Beijing SPF Biotechnology, China) were fed in a pathogen-free environment in PLA general Hospital Animal Laboratory center with access to water and food, and allowed to eat and drink ad libitum. The female C57BL/6J mice aged 8–10 weeks old were randomly separated into different treatment groups using an online randomization tool developed by GraphPad. Doxorubicin exposure model of DOXIC was performed in mice with an intraperitoneal injection of 5 mg/kg every one week on day 0, day7, day 14, day 21, and day 28 (cumulating dose of 25 mg/kg of body weight). Control animals received an identical volume of PBS (Vehicle). The animal protocols met the requirements of the Institutional Animal Care and Use Committee and were authorized by the PLA General Hospital Animal Care and Use Committee. All animal experiments complied with applicable ethical guidelines for using animals in research.

### 2.3 Statistical analysis

Continuous data are expressed as mean ± SD unless otherwise specified. Normality was checked using the Kolmogorov-Smirnov test, and non-normal data were log-transformed before analysis. Comparison between two or more groups was performed using the students’ *t*-test and ANOVA for normal variables. To analyze data from experiments with 2-group and 2-treatment, 2-way ANOVA was used, and the effect of group and treatment were analyzed. All tests were two tailed. Bonferroni adjustment was used to account for multiple testing. Biological replicates (individual mice or biological replicates) are shown as individual data points superimposed to bar charts. Significance was accepted at p < 0.05.

## 3 Results

### 3.1 Breast cancer cells coculture increases the susceptibility of DOX-treated cardiomyocytes to ferroptosis

**Fig. 1:**
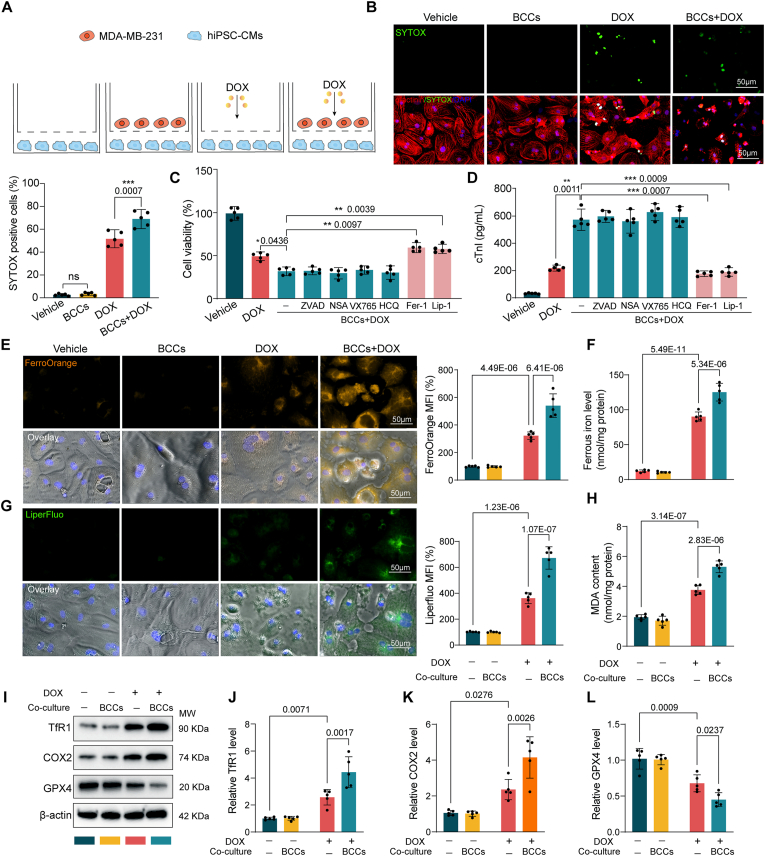
Breast cancer cells coculture increased the susceptibility of DOX-treated cardiomyocytes to ferroptosis. (A) schematic diagram showing the coculture model of breast cancer cells (MDA-MB-231, 231 cells hereafter) and human induced pluripotent stem cell derived cardiomyocytes (hiCMs) in transwell chambers in the presence or absence of DOX (DOX coculture system); (B) Representative images and quantitative analysis of SYTOX staining denoting cell death of hiCMs after 1 μM DOX treatment for 24 h in the presence or absence of 231 cells; (C-D) hiCMs in the coculture system was challenged with 1 μM DOX in the presence or absence of the indicated regulated cell death inhibitors (ZVAD,10 μM; NSA, 1 μM; VX765, 3 μM; HCQ, 50 μM; Fer-1, 1 μM; Lip-1, 100 nM) for 24 h, and cell viability was assayed (C) and cTnI leakage (D) in the supernatant was examined (n = 5); (E) Representative FerroOrange fluorescent images of hiCMs denoting intracellular ferrous iron deposition were presented and quantitative analysis of fluorescence was performed (n = 5); (F) Ferrous iron level in the cell lysates was quantified by iron assay kit (n = 5); (G) Representative Liperfluo fluorescent images of hiCMs denoting lipid peroxidation levels were presented and quantitative analysis of fluorescence intensities was performed (n = 5); (H) The MDA concentration in cell lysates was measured by assay kit (n = 5); (I) The protein levels of TfR1, COX2, and GPX4 were examined by Western blot in hiCMs (n = 5). Fer-1 (ferrostatin-1); Lip-1 (liproxstatin-1); ZVAD (ZVAD-FMK), NSA (necrosulfonamide); HCQ (hydroxychloroquine); hiCMs (human induced pluripotent stem cell derived cardiomyocytes). All data were collected from at least 3 independent experiments. Unless otherwise indicated, data were analyzed by 1-way ANOVA, followed by Bonferroni post hoc test.

To decipher the potential interaction of breast cancer cells (BCCs) with cardiomyocytes in DOX-induced cardiotoxicity (DOXIC), a transwell based coculture system was employed as depicted in Fig. 1A. Human induced pluripotent stem cell derived cardiomyocytes (hiCMs) was cocultured with human breast cancer cell (BCC) line MDA-MB-231(abbreviated as 231 cells hereafter), and treated with 1 μM DOX for 24 h. Intriguingly, the presence of 231 cells in the coculture system intensified DOX-induced hiCM cell death as revealed by SYTOX staining (Fig. 1B). Consistently, DOX-treated hiCM experienced more prominent cell viability loss when cocultured with 231 cells (Fig. 1C). In an attempt to determine which regulated cell death (RCD) participates in this process, a series of RCD inhibitors were separately added into the hiCMs upon 231 cells coculture and DOX challenge. The exacerbated cell viability loss induced by 231 cells coculture was rescued by cotreatment with ferroptosis inhibitors ferrostatin-1(Fer-1) and liproxstatin-1 (Lip-1) but not Z-VAD-FMK (ZVAD, an apoptosis inhibitor), necrosulfonamide (NSA, a necroptosis inhibitor), VX765 (a pyroptosis inhibitor), or hydroxychloroquine (HCQ, an autophagy inhibitor), indicating that ferroptosis was the major cell death type (Fig. 1C). The cTnI leakage data further corroborated that ferroptosis inhibitors Fer-1 and Lip-1 rescued hiCMs injury induced by 231 cells coculture (Fig. 1D). To further validate that the exacerbated cell death was indeed ferroptosis, the generally accepted features of ferroptosis including ferrous iron accumulation and lipid peroxidation [24] were also assessed. The results showed that DOX-treated hiCM had higher ferrous iron accumulation (revealed by FerroOrange labile ferrous ion detecting probe and colorimetric ion assay) and lipid peroxidation level (revealed by Liperfluo fluorescent probe) when cocultured with 231 cells (Fig. 1E–G). In addition, malondialdehyde (MDA), a stable end product of lipid peroxidation, further confirmed the increased lipid peroxidation level during DOX treatment in the coculture system (Fig. 1H). Besides, the expression levels of ferroptosis marker genes were verified by Western blots. DOX with coculture led to the up-regulated TfR1 and COX2 (positive markers of ferroptosis), down-regulated GPX4 (a negative marker of ferroptosis) in hiCMs (Fig. 1J). Worthy of note, breast cancer cells coculture in the normal condition (without DOX treatment) did not alter the cell viability and cell death of DOX-challenged hiCM. Collectively, these results suggested the existence of possible deleterious communication between BCCs and hiCMs in response to DOX treatment that could aggravate DOXIC by sensitizing hiCMs to ferroptosis.

### 3.2 Small extracellular vesicles (sEVs) mediate the pathological communications between breast cancer cells and cardiomyocytes and aggravate DOX-induced cardiomyocytes ferroptosis

To determine whether small extracellular vesicles (sEVs) mediated the pathological communications between BCCs and cardiomyocytes in DOXIC, we depleted sEVs from 231 cells by pretreating cells with sEVs secretion inhibitor GW4869 (10 μM, 48 h) before coculture [25]. The results showed that pretreatment of 231 cells with GW4869 abolished the increase in cell viability loss and cTnI leakage in hiCMs cocultured with 231 cells and exposed to DOX treatment (Supplementary Fig. 1A and B). Nanoparticle tracking analysis (NTA) revealed that GW4869 effectively reduced sEVs production (Supplementary Fig. 1C and D). This suggest that the aggravated DOXIC in cardiomyocytes cocultured with BCCs is attributed to sEV transmission from 231 cells to hiCMs. To further validate that 231 cells-derived sEVs were involved in coculture-exacerbated hiCMs injury, we examined the expression changes of Rab27a and Rab27b, Rab-GTPases indispensable for sEV secretion in mammalian cells [26]. We observed a significant induction of mRNA level of Rab27a but not Rab27b in DOX-challenged 231 cells (Supplementary Fig. 1E). Next, short hairpin RNAs (shRNAs) targeting Rab27a and Rab27b were synthesized and used to transduce 231 cells. The efficacy of specific targeting of two shRNAs were validated (Supplementary Fig. 1F). ShRNA targeting Rab27a but not Rab27b depleted sEV release from DOX-challenged 231 cells, as measured by nanoparticle tracking assay (Supplementary Fig. 1G and H), which elucidated that Rab27a silencing is effective in suppressing sEV secretion from 231 cells. Moreover, Rab27a silencing phenocopied the effects of GW4869 in abolishing coculture-exacerbated hiCMs cell viability loss and cTnI leakage (Supplementary Fig. 1I and J). In addition, both GW4869 pretreatment and Rab27a silencing were effective in reversing BCCs coculture-induced ferrous iron accumulation and malondialdehyde generation (Supplementary Fig. 1 K-N), suggesting that BCCs coculture increased the susceptibility of DOX-treated cardiomyocytes to ferroptosis in a sEVs dependent manner.

**Fig. 2:**
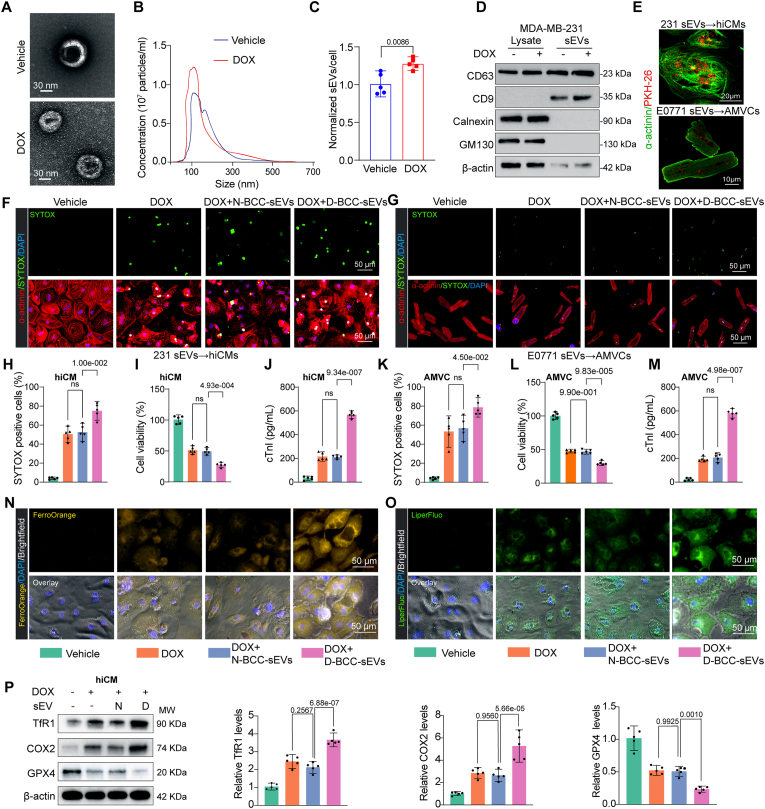
Small extracellular vesicles (sEVs) from DOX-treated breast cancer cells aggravated DOX-induced cardiomyocytes ferroptosis. (A) Transmission electron microscopy (TEM) was used to characterize sEVs from vehicle and DOX-treated breast cancer cells (N-BCC-sEVs and D-BCC-sEVs, respectively); (B) The nanosight tracking analysis (NTA) was used to determine particle size distributions of isolated sEVs; (C) Normalized sEVs secretion per cell, the data were analyzed by Student *t*-test; (D) Immunoblot of N-BCC-sEVs and D-BCC-sEVs probed for positive and negative sEV markers; (E) *In vitro* BCC-sEVs uptake analysis. PKH26-labeled BCC sEVs were harvested from the conditioned culture medium of MDA-MB-231 cells and E0771 cells and subsequently incubated with hiCMs and adult mouse ventricular myocytes (AMVCs), respectively for 6 h; (F, G) Representative images of SYTOX staining denoting cell death of hiCMs (F) and AMVCs (G) after 1 μM DOX treatment for 24 h in the presence or absence of N-BCC-sEVs and D-BCC-sEVs; (H) The SYTOX positive ratio of hiCMs were calculated based on F (n = 5); (I) Cell viability of hiCMs following DOX treatment and 231-sEVs incubation was measured (n = 5); (J) cTnI leakage of hiCMs following DOX treatment and 231-sEVs incubation was measured (n = 5); (K) The SYTOX positive ratio of AMVCs were calculated based on G (n = 5); (L) Cell viability of AMVCs following DOX treatment and E0771-sEVs incubation was measured (n = 5); (M) cTnI leakage of AMVCs following DOX treatment and E0771-sEVs incubation was measured (n = 5); (N) Representative FerroOrange fluorescent images of hiCMs denoting intracellular ferrous iron deposition were presented (n = 5); (O) Representative Liperfluo fluorescent images of hiCMs denoting lipid peroxidation levels were presented; (P) The protein levels of TfR1, COX2, and GPX4 were detected by Western blot in hiCMs following treatment mentioned in F. hiCMs (human induced pluripotent stem cell derived cardiomyocytes); AMVCs (adult mouse ventricular myocytes); All data were collected from at least 3 independent experiments. Unless otherwise indicated, data were analyzed by 1-way ANOVA, followed by Bonferroni post hoc test.

Next, to evaluate the direct effects of doxorubicin-induced breast cancer-cell-secreted sEVs (D-BCC-sEVs) on DOXIC in cardiomyocytes, sEVs were isolated from the conditioned medium of vehicle/DOX-treated 231 cells or murine BCC line E0771 cells and purified with a standard protocol. Using electron microscopy, immunoblotting, and nanoparticle tracking analysis (NTA), we confirmed that isolated particles manifested the characteristic size, morphology, and surface markers of sEVs [23] (Fig. 2A–D). Specifically, we found that DOX treatment significantly upregulated the number of sEVs secreted from 231 cells *in vitro* (Fig. 2B), which was in agreement with a previous study [27]*.* We next incubated cultured hiCM and adult mouse ventricular myocytes (AMVCs) with PKH26-labeled D-BCC-sEVs from 231 cells and E0771 cells, respectively, to probe the internalization of sEVs. We observed that D-BCC-sEVs were successfully internalized by cardiomyocytes after a 6 h incubation as indicated by PKH26 fluorescence distributions (Fig. 2D). Furthermore, compared with sEVs derived from nontreated BCCs (N-BCC-sEVs), sEVs derived from DOX-treated 231 cells (D-BCC-sEVs) markedly worsened DOX-induced cell death, cell viability loss and cTnI leakage in hiCM (Fig. 2F–H-J). These findings were corroborated in murine E0771 breast cancer cells and AMVCs (Fig. 2G–K-M). Ferroptosis related assays revealed that D-BCC-sEVs but not N-BCC-sEVs incubation increased intracellular ferrous iron accumulation and lipid peroxidation in DOX-treated hiCMs (Fig. 2N and O, Supplementary Fig. 2A–D). Intracellular glutathione (GSH) is a key antioxidant against ferroptosis [28]. Examination of intracellular GSH and its oxidized state glutathione disulfide (GSSG) revealed that GSH reduced, GSSG accumulated and the ratio of GSH/GSSG decreased in D-BCC-sEVs-challenged hiCMs exposed to DOX (Supplementary Fig. 2E–G). Furthermore, Western blot data showed that D-BCC-sEVs increased the expression levels of ferroptosis positive markers TfR1 and COX2 whereas decreased GPX4 expression (Fig. 2P). Collectively, these results suggested that D-BCC-sEVs exacerbated DOXIC through intensifying ferroptosis in cardiomyocytes.

### 3.3 D-BCC-sEVs exacerbate DOX-induced myocardial injury and ferroptosis in mice

**Fig. 3:**
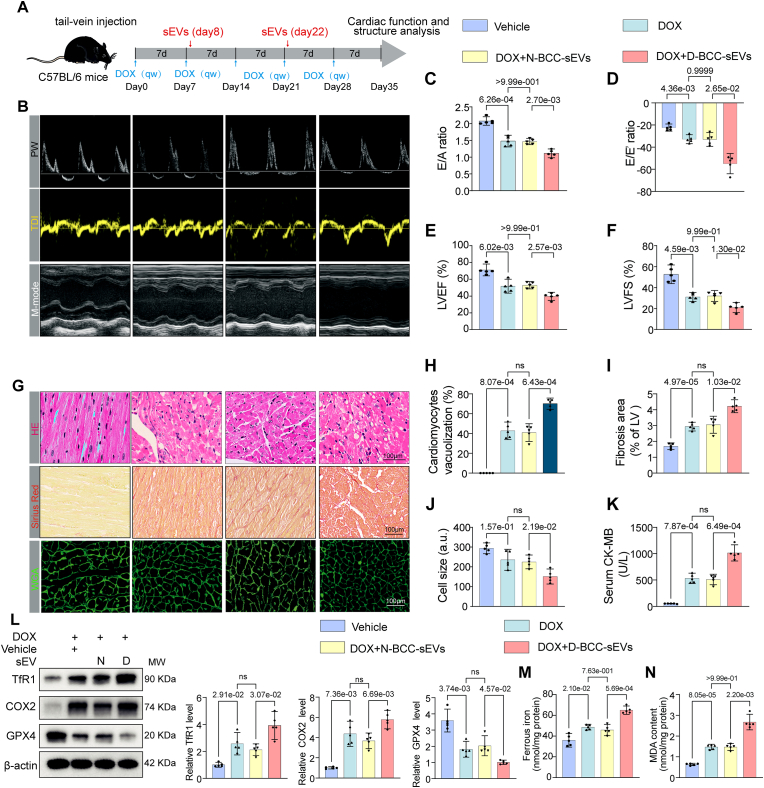
sEVs secreted from DOX-treated breast cancer cells (D-BCC-sEVs) exacerbate DOX-induced myocardial injury and ferroptosis in mice. (A) Schematic illustration showing DOX exposure and tail-vein injection of sEVs from vehicle and DOX-treated E0771 breast cancer cells (N-BCC-sEVs and D-BCC-sEVs, respectively) in C57BL/6J mice; (B) Representative echocardiographic images to assess the cardiac function including Pulse-Wave Doppler (PW), Tissue Doppler Imaging (TDI) and M-mode images in DOX exposure and sEVs-treated mice; Quantitative analysis of E/A ratios (C), left ventricular ejection fraction (LVEF) (D), left ventricular fraction shortening (LVFS) (E), E/E′ ratio (F) in DOX exposure and sEVs-treated mice (n = 5); (G) Representative images of hematoxylin and eosin staining to visualize myocardial histological changes (upper), Sirius red staining (middle) to indicate myocardial fibrosis, and wheat germ agglutinin (WGA) immunofluorescence staining (lower) to evaluate myocardial atrophy; (H) The statistics of cardiac vacuolization ratio in ventricular tissues (n = 5); (I) The fibrotic area per left ventricle was quantified based on Sirius red staining (n = 5); (J) Cell size was quantified based on WGA staining (n = 5); (K) Serum level of Creatine kinase-MB (CK-MB) was detected by colorimetric method; (L) The protein levels of TfR1, COX2, and GPX4 were detected and quantified by Western blot in murine ventricular tissues (n = 5); (M) Ferrous iron level in the homogenization of cardiac tissues was quantified by iron assay kit (n = 5); (N) The MDA concentration in the homogenization of cardiac tissues was measured by assay kit (n = 5); E/A, the ratio of the peak early transmitral flow velocity to the peak late transmitral flow velocity; E/E′, the ratio of the peak early transmitral flow velocity to the peak early diastolic mitral annular velocity. All data were collected from at least 3 independent experiments. Unless otherwise indicated, data were analyzed by 1-way ANOVA, followed by Bonferroni post hoc test.

To determine whether D-BCC-sEVs were able to aggravate DOX-induced myocardial injury *in vivo*, sEVs derived from DOX-treated syngeneic E0771 breast cancer cells (D-BCC-sEVs) or normal control E0771 cells (N-BCC-sEVs) were intravenously administered to DOX-treated C57BL/6J mice without tumor (Fig. 3A). D-BCC-sEVs but not N-BCC-sEVs significantly aggravated DOX-induced cardiac dysfunction, as revealed by more pronounced reductions in left ventricular ejection fraction (LVEF), left ventricular fractional shortening (LVES), E/A, and E/Eʹratios (Fig. 3B–F). Furthermore, histological hematoxylin and eosin (H&E) staining revealed that D-BCC-sEVs treatment increased DOX-induced vacuolization of left ventricular tissue (Fig. 3G and H). Sirius red staining demonstrated that D-BCC-sEVs enhanced DOX-induced cardiac fibrosis (Fig. 3G and I). Wheat germ agglutinin (WGA) staining revealed that D-BCC-sEVs aggravated DOX-induced myocardial atrophy (Fig. 3G and J). In addition, serum Creatine Kinase MB (CK-MB), an indicator of cardiac injury, was also significantly induced by D-BCC-sEVs (Fig. 3K). Worthy of note, Western blot revealed that D-BCC-sEVs significantly augmented ferroptosis-related positive markers TfR1 and COX2 expressions, paralleling with a decline in ferroptosis-related negative marker GPX4 expression (Fig. 3L). Ferroptosis related assays further valiated that D-BCC-sEVs but not N-BCC-sEVs injection increased myocardial ferrous iron accumulation and lipid peroxidation (Fig. 3M and N). These results showed that D-BCC-sEVs exacerbated DOX-induced myocardial injury and ferroptosis *in vivo*.

### 3.4 Blockage of sEVs release from breast cancer tissue (BCT) mitigates doxorubicin toxicity to heart in an orthotopic breast cancer mouse model

To further explore whether breast cancer tissues (BCT) participate in DOXIC aggravating effects by secreting sEVs into circulation, an orthotopic breast cancer mouse model was constructed and lentiviral shRNA targeting Rab27a, which has been proved to effectively inhibit sEVs secretion in BCCs, was intratumorally injected (Supplementary Fig. 3A). In contrast to negative control shRNA (shNC), inhibiting sEVs secretion by shRab27a reversed DOX-induced reductions in cardiac systolic and diastolic functional parameters including LVEF, LVFS, E/A, and E/Eʹ as evaluated by echocardiographic studies (Supplementary Fig. 3B–E), inhibited myocardial vacuolization, fibrosis, and atrophy (Supplementary Fig. 3F–I), and reduced DOX-induced serum levels of Creatine Kinase-MB (CK-MB) (Supplementary Fig. 3J and K). Furthermore, we also examined ferroptosis related markers in the cardiac tissue. Inhibiting sEVs secretion by shRab27a counteracted DOX-induced accumulation of malondialdehyde (MDA) and ferrous iron in cardiac tissue (Supplementary Fig. 3L and M), suggesting that shRab27a prevented myocardial ferroptosis. Furthermore, Western blot-based detection of ferroptosis-related markers including TfR1, COX2 and GPX4 further corroborated that inhibiting sEVs release from BCT relieved myocardial ferroptosis (Supplementary Fig. 3N).

### 3.5 miR-338-3p is required for D-BCC-sEVs to aggravate DOX-induced cardiomyocytes ferroptosis

Many studies have shown that sEVs carry miRNAs as critical regulators responsible for sEV-mediated remote communications between heart and remote organ [14]. Therefore, we determined to investigate whether miRNA cargos delivered by D-BCC-sEVs contributed to its DOXIC aggravating effect. To this end, we first examined the effects of sEVs from Dicer-knockout (KO) 231 cells that depleted all miRNAs [29]. Dicer-KO reversed the DOXIC aggravating effects of D-BCC-sEVs as evidenced by less cell viability loss and cTnI leakage in DOX-treated hiCMs (Supplementary Fig. 4A–C). Moreover, Dicer-KO also abolished the effects of D-BCC-sEVs to aggravate ferroptosis in DOX-treated hiCM as manifested by reduced ferrous iron and MDA levels (Supplementary Fig. 4D and E). These results suggested that the key molecules account for the effects of D-BCC-sEVs were miRNAs.

**Fig. 4:**
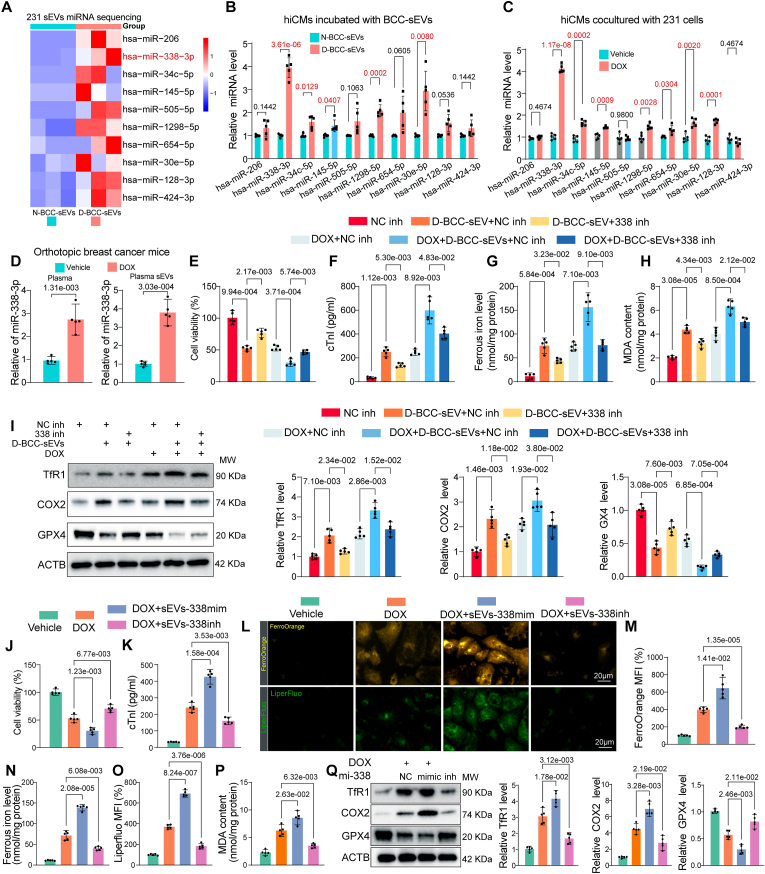
miR-338-3p is required for D-BCC-sEVs to aggravate DOX-induced cardiomyocytes ferroptosis. (A) Heatmap showing differentially upregulated miRNAs in MDA-MB-231 sEVs induced by DOX; (B) qPCR analysis of the 10 upregulated miRNAs in recipient hiCMs incubated with D-BCC-sEVs or N-BCC-sEVs (n = 5), the data were analyzed by unpaired 2-tailed Student *t*-test and multiple comparisons P values were corrected by Holm-Sidak method (10 tests); (C) qPCR analysis of the 10 upregulated miRNAs in hiCMs cocultured with 231 cells and exposed to DOX (n = 5), the data were analyzed by unpaired 2-tailed Student *t*-test and multiple comparisons P values were corrected by Holm-Sidak method (10 tests); (D) qPCR analysis of miR-338-3p level in plasma and plasma sEVs of DOX-challenged orthotopic breast cancer mouse (n = 5); (E) Cell viability and (F) cTnI leakage were measured in hiCMs treated with D-BCC-sEVs in the presence of negative inhibitor (NC inh) or miR-338-3p inhibitor (338 inh) transfection in both normal and DOX conditions (n = 5); (G) Ferrous iron level in the cell lysates of hiCMs following the treatment mentioned in F was quantified by iron assay kit (n = 5); (H) The MDA concentration in cell lysates of hiCMs following the treatment mentioned in F was measured by assay kit (n = 5); (I) The protein levels of TfR1, COX2, and GPX4 were detected and quantified by Western blot in the cell lysates of hiCMs following the treatment mentioned in F (n = 5); (J) Cell viability and (K) cTnI leakage were measured in hiCMs treated with DOX and sEVs from Dicer-KO 231 cells that depleted all endogenous miRNAs and loaded with exogenous miR-338-3p mimic and inhibitor to exclude the potential involvement of other miRNAs (n = 5); (L) Representative Liperfluo (upper panel) denoting lipid peroxidation levels and FerroOrange (lower panel) fluorescent images denoting intracellular ferrous iron deposition in hiCMs were presented and quantitative analysis of fluorescence intensities (M, O) was performed following the treatment mentioned in J (n = 5); Ferrous iron level (N) and MDA concentration (P) in the cell lysates of hiCMs following the treatment mentioned in J was quantified by assay kit (n = 5); (Q) The protein levels of TfR1, COX2, and GPX4 were detected by Western blot in hiCMs following treatment mentioned in J. All data were collected from at least 3 independent experiments. Unless otherwise indicated, data were analyzed by 1-way ANOVA, followed by Bonferroni post hoc test.

To identify the specific functional miRNA in D-BCC-sEVs, differentially expressed miRNAs in D-BCC-sEVs and N-BCC-sEVs were revealed by miRNA sequencing. The results showed that 10 miRNAs were significantly up-regulated and 2 miRNAs were down-regulated (fold change≥2, p < 0.05) (Fig. 4A). The detailed sequencing results are presented in the Supplemental Table Ⅰ. We used qPCR to detect the expression level of the 10 upregulated miRNAs in recipient hiCMs incubated with D-BCC-sEVs or N-BCC-sEVs, as well as in hiCMs cocultured with 231 cells and exposed to DOX. Five of the ten upregulated miRNAs were consistently upregulated in both conditions, including has-miR-338-3p, has-miR-24c-5p, has-miR-145-5p, has-miR-1298-5p, and has-miR-30e-5p (Fig. 4B and C). We then transfected hiCMs with the mimics of 5 candidate miRNAs separately and exposed them to DOX. The results showed that miR-338-3p was the only miRNA that aggravated DOX-induced hiCM injury as evidenced by cell viability loss and cTnI leakage (Supplementary Fig. 4F and G). Furthermore, miR-338-3p was significantly elevated in both plasma and plasma sEVs of DOX-challenged orthotopic breast cancer mouse (Fig. 4D). Besides, miR-338-3p sequence is highly-conserved among mammals (Supplementary Fig. 4H). We thus focused on miR-338-3p and assumed that miR-338-3p might be the specific functional miRNA in D-BCC-sEVs.

To evaluate whether the increased cardiac miR-338-3p is of breast cancer origin and transmitted to recipient heart/cardiomyocytes in a sEV-dependent manner, we first detected the levels of miR-338-3p in orthotopic breast cancer tumor bearing mice and non-tumor-bearing mice, the results showed that miR-338-3p was consistently up-regulated by DOX in plasma, plasma sEVs and cardiac tissues of tumor bearing mice but not in non-tumor-bearing mice or tumor bearing mice subjected to intratumoral injection of lentiviral shRNA targeting Rab27a (Supplementary Fig. 5A–C). Next, we also examined the levels of miR-338-3p in DOX-treated non-tumor-bearing mice intravenously injected with N-BCC-sEVs or D-BCC-sEVs, D-BCC-sEVs but not N-BCC-sEVs overtly increased the levels of miR-338-3p in plasma, plasma sEVs and cardiac tissues (Supplementary Fig. 5D–F). Notably, D-BCC-sEVs failed to enhance the cardiac levels of primary miR-338-3p (pri-miR-338), eliminating the possibility of cardiac endogenous pri-miR-338 transcription (Supplementary Fig. 5G). In addition, we co-transduced 231 cells with PKH67-miR-338-3p-mimic and lentiviral RFP-CD63 and used them as donor cells. Colocalization of PKH67 and RFP was observed in hiCMs recipient cells following incubation with 231/CD63-RFP + PKH67-miR-338-3p cells (Supplementary Fig. 5H). We also transfected the exogenously synthesized miR-338-3p mimic into 231 cells for the subsequent incubation with hiCMs using a transwell system. Notably, the miR-338-3p-overexpressed 231 cells (BCC^338mimic^), but not the negative control (BCC^miR−NC^), showed an efficient miR-338-3p transmission to recipient hiCMs (Supplementary Fig. 5I). However, this transmission was abolished when the 231 donor cells were pretreated with GW4869 or Rab27a shRNA (Supplementary Fig. 5I). Analogously, pretreatment of 231 cells with GW4869 or Rab27a silencing efficiently blocked the transmission of miR-338-3p from donor 231 cells to recipient hiCMs in the DOX-treated coculture system (Supplementary Fig. 5J and K). Notably, D-BCC-sEVs or sEVs isolated from miR-338-3p-overexpressed 231 cells (BCC^338mimic^-sEVs) incubation failed to increase the primary miR-338-3p (pri-miR-338) in hiCMs, demonstrating that increase of mature miR-338-3p in recipient hiCMs following BCC-sEVs incubation was not due to pri-miR-338 transcription (Supplementary Fig. 5L). Moreover, the increased miR-338-3p levels in recipient hiCMs were not affected by the RNA polymerase II inhibitor, actinomycin D (ActD, 1 μg/mL; Supplementary Fig. 5M), which also excluded the involvement of endogenous induction. These results collectively demonstrated that the donor BCC/BCT-released miR-338-3p could be transferred to recipient cardiomyocytes/heart in a sEVs-dependent pathway for remote intercellular/interorgan communication.

To verify whether the D-BCC-sEVs transmitted miR-338-3p to aggravate DOXIC, we first used miR-338-3p inhibitor transfection to interfere with the effects of D-BCC-sEVs. miR-338-3p inhibitor successfully blocked the expression of transmitted miR-338-3p (Supplementary Fig. 5N), as well as the DOXIC aggravating effects of D-BCC-sEVs as evidenced by mitigated cell viability loss and cTnI leakage compared with its negative control inhibitor (Fig. 4E and F). Furthermore, miR-338-3p inhibitor also antagonized the pro-ferroptotic effects of D-BCC-sEVs as manifested by compromised ferrous iron and MDA levels as well as the reversal of ferroptosis marker proteins (Fig. 4G–I). Then, we isolated sEVs from Dicer-KO 231 cells that depleted all endogenous miRNAs and loaded with exogenous miR-338-3p mimic and inhibitor to exclude the potential involvement of other miRNAs. The efficacies of these sEVs in modulating miR-338-3p in recipient hiCMs were validated (Supplementary Fig. 5O). By detecting the changes in cTnI release and cell viability, we demonstrated that sEV-miR-338-3p mimic exacerbated whereas sEV-miR-338-3p inhibitor alleviated DOX-induced cell death in hiCMs (Fig. 4J and K). Additionally, miR-338-3p mimic exacerbated whereas miR-338-3p inhibitor alleviated ferrous iron and MDA accumulation in DOX-treated hiCMs (Fig. 4L–P). Similar changes in ferroptosis marker proteins including TfR1, COX2 and GPX4 were identified using Western blot in hiCMs (Fig. 4Q). Altogether, these findings unravel the necessity of miR-338-3p for D-BCC-sEVs-mediated deterioration of DOX-induced hiCM ferroptosis.

### 3.6 Excessive miR-338-3p maturation *via* N^6^-methyladenosine mediates the upregulation of miR-338-3p in DOX-treated breast cancer cells

**Fig. 5:**
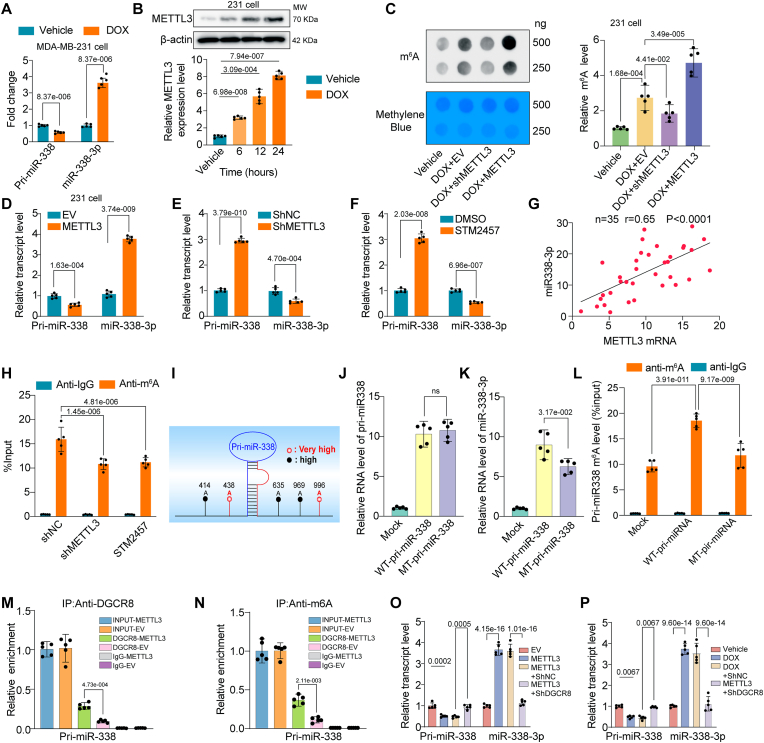
N6-methyladenosine-induced pri-miR-338 maturation mediates the upregulation of miR-338-3p in DOX-treated breast cancer cells. (A) qPCR analysis showed the relative pri-miR-338 and mature miR-338-3p levels in MDA-MB-231 cells treated with DOX (n = 5), the data were analyzed by unpaired 2-tailed Student *t*-test; (B) The protein expression of the major m^6^A methyltransferase methyltransferase-like 3 (METTL3) was examined by Western blot in 1 μM DOX-treated 231 cells at different time points (n = 5); (C) The total m^6^A level in 231 cells treated with DOX and transfected with plasmids that overexpress METTL3 or ShRNA targeting METTL3 (ShMETTL3) was determined by m^6^A dot blot assay. Methylene blue (MB) staining was used as a loading control (n = 5); (D) qPCR analysis showed the relative pri-miR-338 and mature miR-338-3p levels in MDA-MB-231 cells transfected with plasmids that overexpress METTL3 or empty vector (n = 5), the data were analyzed by unpaired 2-tailed Student *t*-test; (E) qPCR analysis showed the relative pri-miR-338 and mature miR-338-3p levels in MDA-MB-231 cells transfected with negative control short hairpin RNA (ShNC) or ShRNA targeting METTL3 (ShMETTL3), the data were analyzed by unpaired 2-tailed Student *t*-test (n = 5); (F) qPCR analysis showed the relative pri-miR-338 and mature miR-338-3p levels in MDA-MB-231 cells treated with DMSO or 10 μM STM2457 for 24 h (n = 5), the data were analyzed by unpaired 2-tailed Student *t*-test; (G) A moderate positive correlation between the expression of METTL3 and miR-338-3p was showed in breast cancer tissues of DOX-treated orthotopic breast cancer mouse; (H) MeRIP assay showing the m^6^A modification level of pri-miR-338 in the presence of METTL3 silence or STM2457 treatment (n = 5); (I) Bioinformatic analysis showed the five potential m^6^A methylation sites resided in the flanking sequence of pri-miR-338; (J) The relative level of pri-miR-338 upon transfected with wild-type pri-miR-338 or mutant plasmids in 231 cells (n = 5); (K) The relative level of miR-338-3p upon transfected with wild-type pri-miR-338 or mutant plasmids in 231 cells (n = 5); (L) MeRIP assay showing the m^6^A modification level of pri-miR-338 upon transfected with wild-type pri-miR-338 or mutant plasmids in 231 cells (n = 5); (M) Detection of pri-miR-338 binding to DGCR8 by immunoprecipitation of DGCR8-associated RNA from control and METTL3 overexpression cells followed by qPCR(n = 5); (N) MeRIP assay based detection of pri-miR-338 m^6^A modification level by immunoprecipitation of m^6^A modified miRNA in control or METTL3 overexpressed 231 cells followed by qPCR (n = 5); (O) qPCR analysis showed the relative pri-miR-338 and mature miR-338-3p levels in empty vector or METTL3-overexpressed 231 cells co-transfected with negative control short hairpin RNA (ShNC) or ShRNA targeting DGCR8 (ShDGCR8) (n = 5); (P) qPCR analysis showed the relative pri-miR-338 and mature miR-338-3p levels in DOX-treated 231 cells transfected with negative control short hairpin RNA (ShNC) or ShRNA targeting DGCR8 (ShDGCR8) (n = 5); All data were collected from at least 3 independent experiments. Unless otherwise indicated, data were analyzed by 1-way ANOVA, followed by Bonferroni post hoc test.

Next, we wondered how miR-338-3p was upregulated in DOX-treated BCCs and whether blockage of this mechanism was effective in interrupting the pathological communications between breast cancer cells and cardiomyocytes. To this end, we first detected the expression levels of primary and mature miR-338-3p in DOX-treated 231 cells. The results show that mature miR-338-3p was upregulated whereas primary miR-338-3p was downregulated in DOX-treated 231 cells, suggesting that the maturation of miR-338-3p was significantly enhanced (Fig. 5A). As N^6^-methyladenosine (m^6^A) modification is an important mechanism contributing to miRNA processing and maturation [30], we surmised that N^6^-methyladenosine might account for the enhanced miR-338-3p maturation. Intriguingly, the major m^6^A methyltransferase methyltransferase-like 3 (METTL3) expression was time-dependently upregulated in DOX-treated 231 cells (Fig. 5B). In agreement with METTL3 level, RNA m^6^A dot blot analyses showed that DOX overtly increased the global m^6^A level of 231 cells, and this increasement could be attenuated and accentuated by METTL3 knockdown and overexpression, respectively (Fig. 5C). Next, we manipulated METTL3 in 231 cells by transfecting 231 cells with plasmids that overexpress METTL3 or ShRNA targeting METTL3 (ShMETTL3) to knockdown METTL3 (Supplementary Supplementary Fig. 8A). We found that the level of mature miR-338-3p was enhanced by METTL3 overexpression but inhibited by METTL3 silence in 231 cells, whereas the primary miR-338 level exhibited the opposite trend (Fig. 5D and E). STM2457, a selective inhibitor of METTL3 followed a pattern that was similar to METTL3 silence to block the maturation of miR-338-3p (Fig. 5F). Furthermore, the correlation analysis revealed a positive correlation between the expression of METTL3 and miR-338-3p transcript level (r = 0.65, P < 0.0001) in breast cancer tissues of DOX-treated orthotopic breast cancer mouse (Fig. 5G). These findings suggested a METTL3/m^6^A-dependent manner for miR-338-3p upregulation in DOX-treated breast cancer cells.

To further assess the mechanistic underpinnings of m^6^A in miR-338-3p upregulation, we immunoprecipitated m^6^A from RNA of negative control and METTL3-silenced 231 cells as well as STM2457-treated 231 cells, m^6^A RNA immunoprecipitation (MeRIP) assays revealed that METTL3 silence or STM2457 treatment significantly decreased the amount of pri-miR-338 modified by m^6^A (Fig. 5H). We further analyzed primary miR-338 sequence and identified several consensus m^6^A motifs (RRACH) within the pri-miR-338 sequence using a sequence-based N^6^-methyladenosine (m^6^A) modification site predictor [31] (Fig. 5I and Supplementary Fig. 8B and C). A total of five putative m^6^A sites with high or very high confidence were identified (Supplementary Fig. 8C). We next validated the m^6^A site within the pri-miR-338 sequence by a single-base elongation- and ligation-based quantitative PCR amplification method (called SELECT) [32] in DOX-induced 231 cells. Only the qPCR cycle threshold (Ct) values of m^6^A 438 decreased upon METTL3 silence and STM2457 treatment, suggesting that this site was the genuine m^6^A site responsive to METTL3 regulation (Supplementary Fig. 8D). To determine whether m^6^A 438 site participated in the m^6^A modification of miR-338-3p, we constructed expression vectors containing wild-type or mutant m^6^A motif. In the mutant vector, adenine in m^6^A 438 site was replaced with guanine. The 231 cells were transfected with equal amounts of the wild-type and mutant pri-miR-338 vectors. The transcript level of pri-miR-338 was comparable between the wild-type and mutant cells (Fig. 5J). However, mutation of the m^6^A 438 site in pri-miR-338 significantly reduced its processing to the mature form (Fig. 5K), with a concomitant reduction in the amount of pri-miR-338 modified by m^6^A (Fig. 5L). These findings confirmed that the m^6^A 438 site is essential in the m^6^A modification of pri-miR-338 that dictates its maturation.

M^6^A-mediated primary miRNA maturation usually requires the recognition and procession by DGCR8 [30]. Herein, we found an increased level of pri-miR-338 binding by DGCR8 immunoprecipitated from METTL3 overexpressed 231 cells. As expected, MeRIP assay revealed that METTL3 overexpression significantly increased the amount of pri-miR338 modified by m^6^A (Fig. 5M and N). Furthermore, DGCR8 was silenced with shRNA to examine its effect on miR-338 maturation (Supplementary Fig. 7A). METTL3 overexpression or DOX treatment-induced miR-338-3p maturation from pri-miR338 was abrogated by DGCR8 silencing in 231 cells (Fig. 5O and P). Taken together, these results indicated that the upregulated METTL3 in response to DOX could enhance the recognition of pri-miR-338 by DGCR8 and the subsequent processing to mature form in an m^6^A/DGCR8 dependent manner.

Next, we sought to investigate whether blockage of the m^6^A-mediated primary pri-miR-338 maturation in BCCs could suppress sEVs-transmitted miR-338-3p and D-BCC-sEVs-induced DOXIC aggravating effects. The transmission of miR-338-3p were compromised when the 231 donor cells were pretreated with shRNAs targeting METTL3 and DGCR8 to silence their expressions, or STM2457 to inhibit METTL3 activity in the coculture system exposed to DOX (Supplementary Fig. 7B–D). Similarly, D-BCC-sEVs derived from METTL3 or DGCR8-silenced or STM2457 pretreated 231 cells abrogated the transmissions of miR-338-3p by D-BCC-sEVs (Supplementary Fig. 7E–G). Consequently, hiCM co-cultured with METTL3 or DGCR8-silenced or STM2457 pretreated 231 cells experienced less DOX-induced cell viability loss and cTnI leakage (Supplementary Fig. 7H and I). Furthermore, detection of ferroptosis indicators including ferrous ion concentration and lipid peroxidation MDA level revealed that D-BCC-sEVs derived from METTL3 or DGCR8-silenced 231 cells mitigated D-BCC-sEVs-aggravated ferroptosis (Supplementary Fig. 7J and K). These data further suggested that blocking miR-338-3p upregulation by interfering its METTL3/m^6^A/DGCR8 maturation mechanism was a feasible strategy to prevent the adverse transmitting of miR-338-3p between breast cancer cells and cardiomyocytes that aggravated DOXIC.

### 3.7 RBMX selectively packages miR-338-3p into sEVs of BCCs

**Fig. 6:**
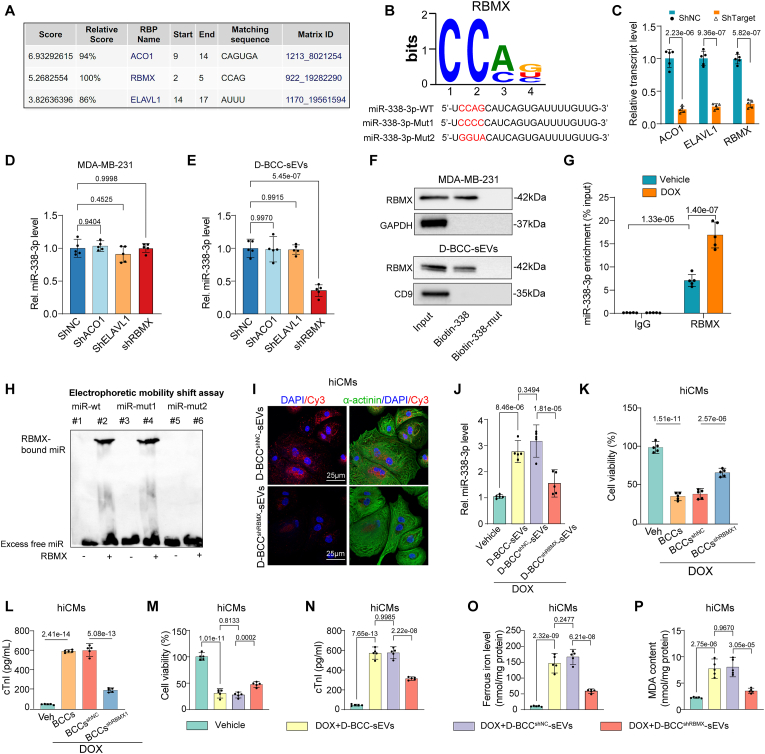
MiR-338-3p is selectively packaged into sEVs by RBMX in breast cancer cells. (A) RBPDB analysis of the specific interaction between miR-338-3p and RBP motifs (threshold 0.8). (B) The “CCA-rich” binding motif of RBMX from RBPDB database was matched with miR-338-3p wildtype and mutated sequences; (C) qPCR analysis to verify the knock down efficacy of shRNAs targeting ACO1, ELAVL1, and RBMX in 231 cells (n = 5); The data in Supplementary Fig. 8C were analyzed by unpaired 2-tailed Student *t*-test and multiple comparisons P values were corrected by Holm-Sidak method; (D, E) Relative levels of miR-338-3p in 231 cells (D) and sEVs from DOX-treated 231 cells (D-BCC-sEVs) (E) transfected with negative control ShRNA (ShNC), ShRNA targeting ACO1 (ShACO1), ShRNA targeting ELAVL1 (ShELAVL1), and shRNA targeting RBMX (shRBMX) (n = 5); (F) The biotin-labeled miR-338-5p wildtype or mutated probe was used to capture RBMX in 231 cells and D-BCC-sEVs by RNA pull down assay (n = 5); (G) RNA immunoprecipitation (RIP) assay using an anti-RBMX antibody followed by qPCR based detection of miR-338-3p in 231 cells (n = 5), data were analyzed by 2-way ANOVA, followed by Bonferroni post hoc test; (H) Electrophoretic mobility shift assay (EMSA) showing the binding between miR-338-3p probe and RBMX; (I) sEVs were isolated from RBMX-silenced 231 cells with Cy3-miR-338-3p co-transfection. After hiCM were incubated with 231-derived sEVs containing Cy3-miR-338-5p, the Cy3 fluorescence was observed in hiCMs under confocal microscopy (n = 5); (J) The effects of RBMX knockdown in 231 cells on miR-338-3p transmission by D-BCC-sEVs from 231 cells to hiCMs were examined by qPCR detection of miR-338-3p level in hiCMs (n = 5); (K, L) Negative control ShRNA (ShNC) or ShRNA targeting RBMX (ShRBMX)-transfected 231 cells were cocultured with hiCMs in a transwell system and challenged with 1 μM DOX for 24 h, cell viability (K) and cTnI leakage (L) were examined (n = 5); (M, N) D-BCC-sEVs were isolated from RBMX silenced or negative control 231 cells (BCC^shRBMX^ and BCC^shNC^, respectively), their effects on DOX-induced cell viability (M) and cTnI leakage (N) in hiCMs were examined (n = 5); (O, P) Ferrous iron level (O) and MDA concentration (P) in the cell lysates of hiCMs following the treatment mentioned in J was quantified by assay kit (n = 5). All data were collected from at least 3 independent experiments. Unless otherwise indicated, data were analyzed by 1-way ANOVA, followed by Bonferroni post hoc test.

Recently, RNA-binding proteins (RBPs) have been revealed to play important roles in sorting and packaging transcripts into sEVs [33]. We next sought to investigate the RBP-mediated sorting and packaging mechanisms of miR-338-3p into sEVs of BCCs. To this end, we firstly explored the possible miR-338-3p binding RBP using the bioinformatic prediction algorithms RBPBD (threshold >0.8) [34]. The results showed that Aconitase 1 (ACO1), RNA Binding Motif Protein X-Linked (RBMX), and ELAV Like RNA Binding Protein 1 (ELAVL1) have specific miR-338-3p binding sites (Fig. 6A). Further research revealed that knockdown of RBMX but not ACO1 and ELAVL1 in 231 cells by specific shRNAs significantly reduced sEVs miR-338-3p levels while intracellular miR-338-3p levels remained almost unchanged (Fig. 6C–E), suggesting that RBMX might mediate the package of miR-338-3p into sEVs. The RBMX binding motif and the matched miR-338-3p sequence and its mutated forms were shown in Fig. 6B. Next, we performed an RNA pull-down assay to identify whether RBMX existed in the pull-down product of miR-338-3p. The results demonstrated that wildtype miR-338-3p probe was able to capture RBMX efficiently in 231 cells as well as in sEVs of 231 cells. However, miR-338-3p probe binding ability to RBMX was obliterated when the “CCAG” sequence of miR-338-3p was mutated (Fig. 6F). To further confirm their interaction, we performed an RNA immunoprecipitation (RIP) assay using an anti-RBMX antibody in 231 cells, which showed that miR-338-3p was enriched in the immunoprecipitates of RBMX (Fig. 6G). In order to further confirm the binding sites between miR-338-3p and RBMX, wildtype or mutated versions of miR-338-3p were tested for binding to RBMX in an electrophoretic mobility shift assay (EMSA). The first mutation (miR-338-3p-mut1) was only partial, as it preserved the motif and change the last base from G to A. The second mutation (miR-338-3p-mut2) was complete, as the purines and pyrimidines were exchanged throughout the motif (Fig. 6B–H). EMSA revealed that native miR-338-3p and the partially mutated miR-338-3p-mut1 were shown to preserve the binding ability to RBMX, whereas binding was absent for miR-338-3p-mut2 (Fig. 6H). Next, we knocked down RBMX in 231 cells with Cy3-miR-338-3p co-transfection and isolated the sEVs. After hiCM were incubated with 231-derived sEVs containing Cy3-miR-338-5p, the Cy3 fluorescence was observed in hiCM, whereas RBMX knockdown in 231 cells led to a sharp decrease of Cy3 fluorescence, indicating that RBMX knockdown reduced sEVs-transmitted miR-338-3p from 231 cells to hiCMs (Fig. 6I), this result was also confirmed by qPCR showing that RBMX knockdown in 231 cells reduced miR-338-3p transmission by D-BCC-sEVs from 231 cells to hiCMs (Fig. 6J). These results further confirmed that specific binding between miR-338-3p and RBMX accounted for miR-338-3p packaging into sEVs of BCCs.

To further evaluate the functional significance of this packaging mechanism in the pathological communication between BCCs and cardiomyocytes in DOXIC, we silenced RBMX in 231 cells and cocultured RBMX-silenced 231 cells with hiCM and exposed them to DOX. The result showed that BCCs coculture-aggravated hiCM cell viability loss, cTnI leakage could be assuaged by RBMX knockdown in 231 cells (Fig. 6K and L). Moreover, RBMX silence in 231 cells abrogated the cell viability loss and cTnI leakage exacerbated by D-BCC-sEVs in DOX-treated hiCMs (Fig. 6M and N). Furthermore, examination of ferroptosis indicators including ferrous ion concentration and MDA level revealed that RBMX silence in 231 cells successfully mitigated D-BCC-sEVs-aggravated ferroptosis in DOX-treated hiCMs (Fig. 6O and P). These data further corroborated the pivotal role of RBMX in transmitting miR-338-3p from BCCs to cardiomyocytes in DOXIC and suggested that blocking miR-338-3p transmitting by interfering its RBMX-mediated packaging mechanism was effective in preventing BCC-induced DOXIC aggravating effects and the detrimental communication between breast cancer cells and cardiomyocytes in DOXIC.

### 3.8 miR-338-3p aggravates DOX-induced cardiomyocytes ferroptosis by targeting CP/SLC7a11/GPX4

**Fig. 7:**
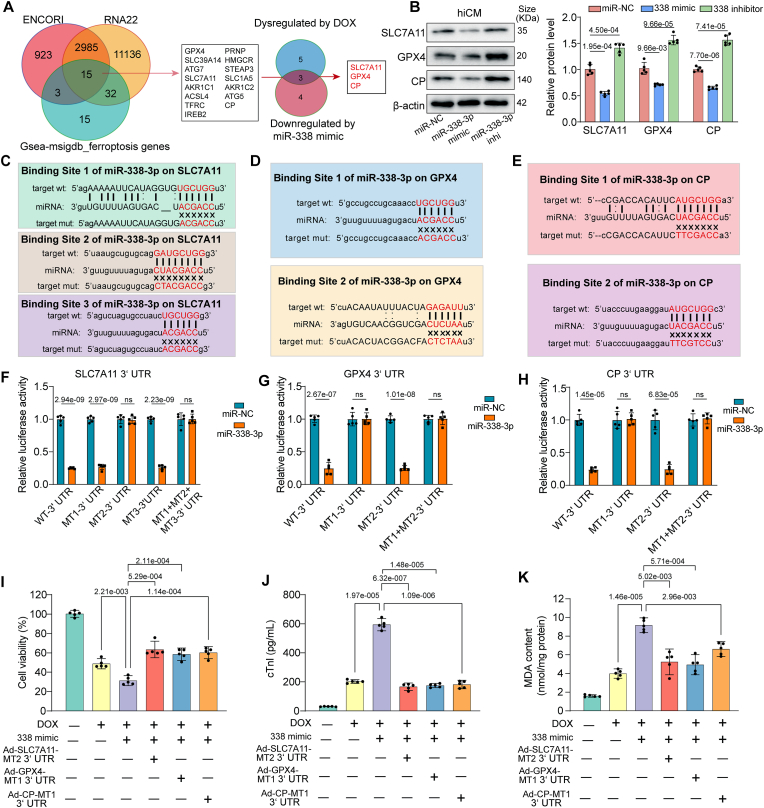
miR-338-3p aggravates DOX-mediated cardiomyocytes ferroptosis by targeting Ceruloplasmin (CP), Solute Carrier Family 7 Member 11 (SLC7A11), and Glutathione Peroxidase 4(GPX4). (A) Potential miR-338-3p targets were selected by intersecting the predicted targets from“ENCORI” and “RNA22” database, with ferroptosis related genes derived from MSigDB-GSEA and 15 putative targets were identified; The expression changes of these 15 putative targets in response to miR-338-3p overexpression and DOX challenge were then examined by qPCR, overlapping the DOX-dysregulated and miR-338-3p mimic-downregulated targets showed that Ceruloplasmin (CP), Solute Carrier Family 7 Member 11 (SLC7A11), and Glutathione Peroxidase 4(GPX4) were altered under both conditions, suggesting that these 3 genes might be the functional downstream targets of miR-338-3p; (B) The protein levels of CP, SLC7A11, and GPX4 in hiCMs transfected with miR-338-3p inhibitor or miR-338-3p mimic were measured by Western blot and quantitative analysis were performed; (C–E) Schematic illustration showing putative binding sites for miR-338-3p in the 3′-UTR sequences of CP, SLC7A11, and GPX4 mRNAs. The wild-type 3′-UTR sequences and the mutant 3′-UTR sequences of the CP, SLC7A11, and GPX4 mRNAs were cloned into construct reporter plasmids and mutant vectors, respectively; (F–H) Cotransfection of miR-338-3p mimic or miR-338-3p negative control mimic with 3′-UTR wildtype reporter plasmids or mutated reporter plasmids of CP, SLC7A11, and GPX4 was followed by assays of luciferase activity (n = 5); (I) CCK8-based cell viability assay to detect miR-338-3p-overexpressed hiCMs viability post DOX treatment (1 μM, 24 h) with co-expression of mutated CP, SLC7A11, and GPX4 3′-untranslated regions (UTRs) (n = 5); (J) cTnI leakage in the supernatants of hiCMs following the treatment mentioned in I (n = 5); (K) MDA concentration in the cell lysates of hiCMs following the treatment mentioned in I (n = 5); All data were collected from at least 3 independent experiments. The data in 7B, I, J, K were analyzed by 1-way ANOVA, followed by Bonferroni post hoc test. The data in 7F, G, H were analyzed by unpaired 2-tailed Student *t*-test and multiple comparisons P values were corrected by Holm-Sidak method (4 tests for G, H; 5 tests for F).

To explore the precise molecular mechanism by which miR-338-3p exacerbates DOX-induced cardiomyocytes injury and ferroptosis, we employed predictor tools to identify the downstream targets of miR-338-3p. We intersected the predicted miR-338-3p targets from “ENCORI” and “RNA22”, the two widely used target predictors for miRNAs, with ferroptosis related genes derived from MSigDB-GSEA and identified 15 putative target genes (Fig. 7A). Among the 15 putative targets, we used two steps to validate the genuine targets mediating the DOXIC aggravating effects of miR-338-3p. First, hiCMs were transfected with miR-338-3p mimic. Second, hiCMs were exposed to 1 μM DOX for 24 h. The mRNA levels of the 15 putative targets were examined by qPCR in both conditions (Fig. 7A, Supplementary Fig. 8A and B). Overlapping the DOX-dysregulated and miR-338-3p mimic-downregulated targets showed that Ceruloplasmin (CP), Solute Carrier Family 7 Member 11 (SLC7A11), and Glutathione Peroxidase 4 (GPX4) were altered under both conditions, suggesting that these 3 genes might be the functional downstream targets of miR-338-3p (Fig. 7A). Consistently, CP, SLC7A11, and GPX4 protein levels in hiCM were positively regulated by miR-338-3p inhibitor and negatively regulated by miR-338-3p mimic (Fig. 7B).

We next experimentally established CP, SLC7A11, and GPX4 as target genes of miR-338-3p by the following steps. First, we performed computational target-scan analysis and identified that SLC7A11 has 3, GPX4 has 2, and CP has 2 predicated binding sites for miR-338-3p in their 3′-untranslated regions (UTRs; Fig. 7C–E). Second, we cloned luciferase reporter plasmids with full-length wild-type 3′-UTR regions of CP, SLC7A11, and GPX4 (WT-3′-UTR) and performed a dual-luciferase reporter assay in AC16 cells. MiR-338-3p effectively reduced luciferase activities from all 3 reporters, demonstrating that CP, SLC7A11, and GPX4 3′-UTRs are direct targets of miR-338-3p (Fig. 7F–H).

To identify which sites are responsible for miR-338-3p and those 3′-UTR interactions, we generated luciferase reporter plasmids with each binding site and the combined mutations and detected luciferase activities from each (mutation details exhibited in Fig. 7C–E). As shown in Fig. 7F–H, miR-338-3p mimic cotransfection suppressed luciferase activity in AC16 cells expressing SLC7A11-MT1-3′-UTR, SLC7A11-MT3-3′-UTR, GPX4-MT2-3′-UTR, CP-MT2-3′-UTR, but not in cells expressing SLC7A11-MT2-3′-UTR, SLC7A11-MT1+MT2+MT3-3′-UTR, GPX4-MT1-3′-UTR, GPX4-MT1+MT2-3′-UTR, CP-MT1-3′-UTR, and CP-MT1+MT2-3′-UTR, suggesting that the binding site 2 of SLC7A11, binding site 1 of GPX4, and binding site 1 of CP mRNA 3′-UTRs interacts with miR-338-3p.

To further corroborate that miR-338-3p aggravated DOX-induced cardiomyocytes injury and ferroptosis through the interaction of miR-338-3p with those 3′-UTRs, we employed adenovirus expressing CP, SLC7A11, and GPX4 mRNA containing mutated 3′-UTR binding site, which has been identified as miR-338-3p resistant. All of the SLC7A11, GPX4 and CP 3′-UTR mutation prevented the increased hiCM viability loss, cTnI leakage and ferroptosis markers MDA content, ferrous iron, GSH loss, and GSSG accumulation induced by miR-338-3p mimic in DOX-treated hiCM (Fig. 7I–K and Supplementary Fig. 8C–E). Of note, SLC7A11 and GPX4 3′-UTR mutation were more effective in reversing GSH and GSSG, whereas CP1 3′-UTR mutation behaved better in averting ferrous iron accumulation (Supplementary Fig. 8 C-E).

### 3.9 Dual-functional decoying sEVs encapsulated with miR-338-3p inhibitor mitigated DOXIC in an orthotopic breast cancer mouse model

To further explore whether blocking the pathogenic miR-338-3p transmission was effective in assuaging DOX-induced cardiac injury in tumor bearing mice, herein we adopted a previously reported mesenchymal stem cell-derived decoying sEVs with the modified “G-C” abundant tetrahedral DNA nanostructure (TDN) and engineered cardiac targeting peptide (CTP) [35] to deliver miR-338-3p inhibitor (sEVs^TDN−CTP^-338-inh). This decoying sEVs (sEVs^TDN−CTP^) has been revealed to significantly detoxify DOX-induced direct heart injury by capturing and decoying DOX and preventing it from entering the nucleus [35]. By combining sEVs^TDN−CTP^ with miR-338-3p inhibitor, we wished to target the direct and indirect tumor-heart injury mechanism of DOXIC that we reported in this study simultaneously and examined whether they have a synergistical effect in alleviating DOXIC in tumor-bearing mice. We first successfully isolated and identified bone marrow derived mesenchymal stem cells (BMSCs) as we have previously reported [36] (Supplementary Fig. 9A). The sEVs were then extracted and purified from the serum-free medium of the BMSCs by ultracentrifugation. Using electron microscopy, immunoblotting, and nanoparticle tracking analysis (NTA), we confirmed that isolated particles manifested the characteristic morphology, surface markers and size of sEVs (Supplementary Fig. 9B–D). Next, we synthesized cholesterol-modified TDNs and engineered them onto the sEVs. Dynamic light scattering (DLS) showed that the particle size of the TDN was around 10 nm (Supplementary Fig. 9E and F). Agarose gel electrophoresis showed the successful synthesis of TDN and successful loading of TDN onto the sEVs (Supplementary Fig. 9G and H). Zeta potential analysis also validated the successful graft of TDN onto sEVs (Supplementary Fig. 9I). Consistent with previous study, when sEVs and TDN reacted with a mass ratio of 1:3 or 1:4, the grafting efficiency of TDN reached the maximum rate (Supplementary Fig. 9J). We further examined the capture efficiency of sEVs^TDN^ to DOX. The incubation of sEVs^TDN^ significantly quenched the fluorescence intensity of DOX, hindering it from entering the nucleus of hiCMs, and the capture efficiency of sEVs^TDN^ reached up to ∼80 % (Supplementary Fig. 9K–M). Next, we functionally characterized sEVs^CTP^ by studying its cardiac targeting ability. We intravenously injected DiR (1,1“-Dioctadecyl-3,3,3”,3′-Tetramethylindodicarbocyanine,4-Chlorobenzenesulfonate Salt)-labeled sEVs^TDN^ or sEVs^TDN−CTP^, and then use the small animal *in vivo* imaging system (IVIS) to examine their biodistribution. As shown in Supplementary Fig. 9N, sEVs^TDN^ was mostly enriched in the liver area, whereas sEVs^TDN−CTP^ was partially accumulated in the myocardial area 4 h post-delivery. In addition, PKH67 labeled sEVs^TDN^ and sEVs^TDN−CTP^ were incubated with hiCMs for 6 h and the significantly more enrichment of sEVs^TDN−CTP^ in the cytoplasm of hiCMs was observed compared with sEVs^TDN^ without CTP (Supplementary Fig. 9O). Furthermore, we also validated the successful loading of miR-338-3p inhibitor into sEVs^TDN−CTP^. As shown in Supplementary Fig. 9P, D-BCC-sEVs aggravated DOX-induced cell viability loss in hiCMs, this deteriorating effect could be slightly ameliorated by sEVs from MSCs and further improved by sEVs^TDN^. Either targeting the indirect injury mechanism by sEVs^CTP^ encapsulating miR-338-3p inhibitor (sEVs^CTP^-338-inh) or targeting the direct injury mechanism by sEVs^TDN−CTP^ encapsulating negative control inhibitor (sEVs^TDN−CTP^ NC-inh) effectively alleviated D-BCC-sEVs-aggravated DOX-induced cell viability loss, whereas simultaneously targeting by sEVs^TDN−CTP^ encapsulating miR-338-3p inhibitor (sEVs^TDN−CTP^-338-inh) exhibited the best protection against cell viability loss. Furthermore, we also examined the miR-338-3p level in hiCMs exposed to various BMSC sEVs incubation. D-BCC-sEVs induced significant upregulation of miR-338-3p in DOX-treated hiCMs, this upregulation could be abrogated by sEVs^CTP^-338- inhibitor and sEVs^TDN−CTP^-338- inhibitor, but not by sEVs^TDN−CTP^-NC-inhibitor (Supplementary Fig. 9Q). These findings proved the preliminary efficacy of combining sEVs^TDN−CTP^ with miR-338-3p inhibitor by sEVs^TDN−CTP^-338-inhibitor for the treatment of DOXIC.

**Fig. 8:**
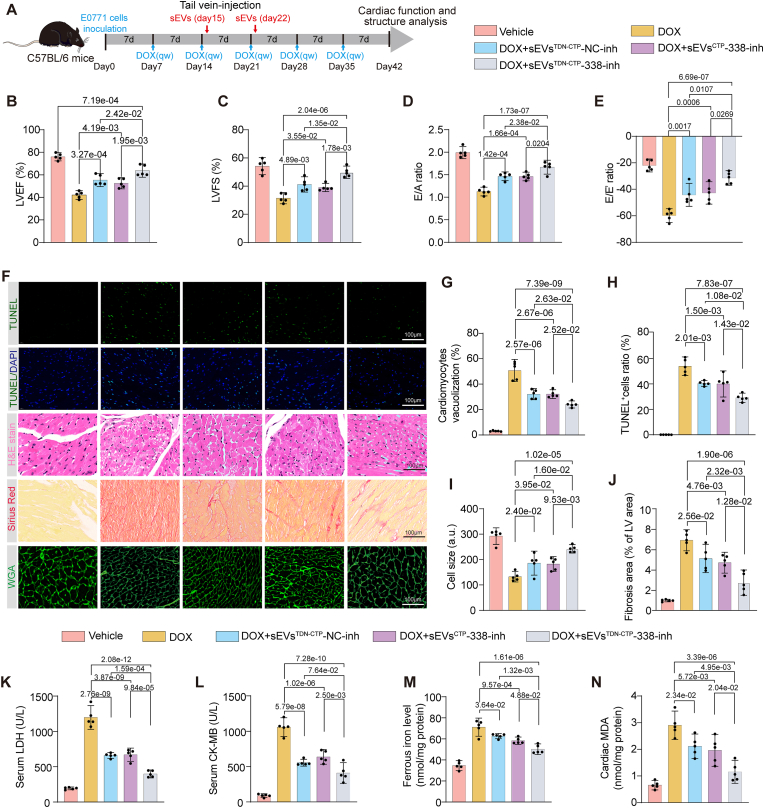
Dual-functional decoying sEVs encapsulated with miR-338-3p inhibitor mitigate DOXIC in tumor bearing mice. (A) Schematic illustration depicting DOX exposure and tail-vein injection of dual-functional decoying sEVs in an orthotopic breast cancer mouse model; Echocardiographic analysis of left ventricular ejection fraction (LVEF) (B), left ventricular fraction shortening (LVFS) (C), E/A ratios (D), and E/E′ ratio (E) in DOX exposure and sEVs-treated mice (n = 5); (F) Representative images of Terminal deoxynucleotidyl transferase dUTP nick end labeling (TUNEL) staining to evaluate cell death (First two layers), hematoxylin and eosin staining to visualize myocardial histological changes (Third layer), Sirius red staining (Fourth layer) to indicate myocardial fibrosis, and wheat germ agglutinin (WGA) immunofluorescence staining (Fifth layer) to evaluate myocardial atrophy; (G) The statistics of cardiac vacuolization ratio in ventricular tissues (n = 5); (H) The statistics of TUNEL positive ratio in ventricular tissues (n = 5); (I) Cell size was quantified based on WGA staining (n = 5); (J) The fibrotic area per left ventricle was quantified based on Sirius red staining (n = 5); Serum levels of Lactate dehydrogenase (LDH) (K) and Creatine kinase-MB (CK-MB) (L) were detected by colorimetric method; Ferrous iron level (M) and MDA concentration (N) in the homogenization of cardiac tissues was quantified by respective assay kit (n = 5); All data were collected from at least 3 independent experiments. Unless otherwise indicated, data were analyzed by 1-way ANOVA, followed by Bonferroni post hoc test.

Next, we employed these engineered therapeutic BMSC sEVs in DOX-induced cardiac injury in orthotopic breast cancer mice. Two doses of therapeutic sEVs were intravenously delivered at day 15 and day 22 post E0771 cells inoculation when the cardiac dysfunction became evident as revealed by a serial echocardiography examination (Supplementary Figs. 10A, B,Fig. 8A). Either targeting the indirect mechanism by sEVs^CTP^-338-inhibitor or targeting the direct mechanism by sEVs^TDN−CTP^-NC-inhibitor effectively reversed DOX-induced reductions in cardiac systolic and diastolic functional parameters comprising LVEF, LVFS, E/A, and E/Eʹ (Fig. 8B–F), retarded DOX-induced myocardial vacuolization, fiber breakage, fibrosis, and atrophy (Fig. 8G–J), and reduced DOX-induced serum levels of lactate dehydrogenase (LDH) and Creatine Kinase-MB (CK-MB) (Fig. 8K–L). Notably, sEVs^TDN−CTP^-338-inhibitor exhibited the highest efficacy in restoring DOX-induced cardiac dysfunction (echocardiographic functional parameters) and injury (myocardial vacuolization, fiber breakage, fibrosis, atrophy, LDH and CK-MB), compared with sEVs^CTP^-338-inhibitor or sEVs^TDN−CTP^-NC-inhibitor alone (Fig. 8K–L). Furthermore, either sEVs^CTP^-338-inhibitor or sEVs^TDN−CTP^-NC-inhibitor antagonized DOX-induced accumulation of malondialdehyde (MDA) and ferrous iron in cardiac tissues, these effects were more pronounced in sEVs^TDN−CTP^-338-inhibitor-treated mice (Fig. 8M and N). These data further elucidated that dual-functional decoying sEVs encapsulated with miR-338-3p inhibitor was a potential strategy to combat both the direct and indirect injury of DOXIC in breast cancer bearing mice.

### 3.10 sEVs from patients with DOX-induced cardiomyopathy aggravated cardiomyocytes injury and ferroptosis, which can be reversed by miR-338-3p inhibitor

**Fig. 9:**
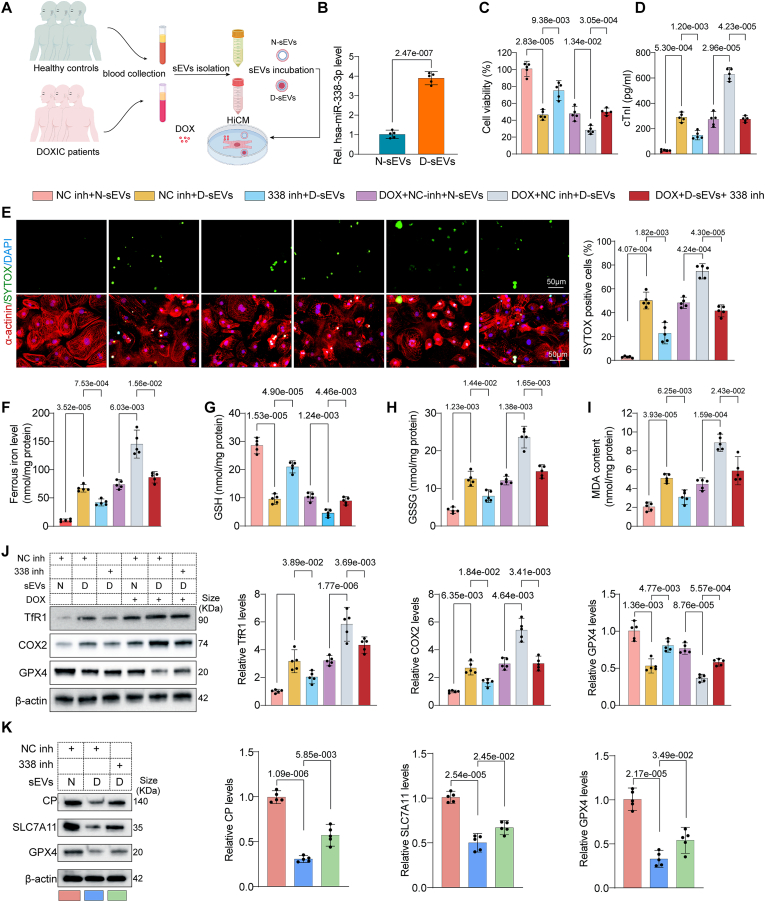
sEVs from patients experiencing DOXIC aggravate DOX-induced hiCMs injury and ferroptosis, which can be reversed by miR-338-3p inhibitor. (A) Schematic illustration depicting the preparation of sEVs isolated from Patients experiencing DOXIC and age and sex matched healthy donors (N-sEVs) and these sEVs were incubated with DOX-treated hiCMs; (B) qPCR analysis of miR-338-3p level in N-sEVs and D-sEVs (n = 5); The data were analyzed by unpaired 2-tailed Student *t*-test; Cell viability (C) and cTnI leakage (D) were measured in hiCMs treated with N-sEVs or D-sEVs in the presence of negative inhibitor (NC inh) or miR-338-3p inhibitor (338 inh) transfection in both normal and DOX conditions (n = 5); (E) Representative images and quantitative analysis of SYTOX staining denoting cell death in hiCMs following the treatment mentioned in C (n = 5); Ferrous iron level (F), glutathione (GSH) (G), oxidized state glutathione disulfide (GSSG) (H) and MDA concentration (I) in the cell lysates of hiCMs following the treatment mentioned in C were quantified by respective assay kit (n = 5); (J) The protein levels of TfR1, COX2, and GPX4 were detected by Western blot in hiCMs following treatment mentioned in C (n = 5); (K) The protein levels of CP, SLC7A11, and GPX4 were detected by Western blot in hiCMs following N-sEVs or D-sEVs incubation in the presence of negative control inhibitor (NC inh) or miR-338-3p inhibitor (338 inh) transfection (n = 5). All data were collected from at least 3 independent experiments. Unless otherwise indicated, data were analyzed by 1-way ANOVA, followed by Bonferroni post hoc test.

As a final step to evaluate the clinical relevance of our experimental findings, we isolated the sEVs from the plasmas of five breast cancer patients who have been treated with DOX and diagnosed as DOX-induced cardiomyopathy to verify whether the plasma sEVs from these patients have similar effects. sEVs were isolated from patients experiencing DOXIC (D-sEVs) and age and sex matched healthy donors (N-sEVs) (Fig. 9A, patients characteristics were summarized in Supplemental Table Ⅱ). sEVs characterization were performed using electron microscopy, immunoblotting, and nanoparticle tracking analysis (NTA) (Supplementary Fig. 11A–C). Interesting, miR-338-3p was significantly increased in D-sEVs compared with N-sEVs (Fig. 9B). Moreover, we found that D-EXOs aggravated DOX-induced cardiomyocyte injury, as evidenced by elevations in SYTOX staining positive cell death ratio, cell viability loss, and cTnI leakage (Fig. 9C–E). These effects could be reversed by transfection with miR-338-3p inhibitor (Fig. 9C–E). Moreover, ferroptosis-related markers including cellular ferrous iron concentration, GSH, GSSG, MDA, and marker proteins TfR1, COX2, and GPX4 levels all pointed to that D-sEVs aggravated DOX-induced cardiomyocyte ferroptosis, this effect was dampened by miR-338-3p inhibitor (Fig. 9F–J). In addition, we also detected the expression levels of miR-338-3p downstream targets CP, SLC7A11, and GPX4. D-sEVs induced a significant reduction in CP, SLC7A11, and GPX4 protein levels, an effect reversed by miR-338-3p inhibitor transfection (Fig. 9K). These results might suggest that sEVs-packaged miR-338-3p mediated the pathological communications between breast cancer cells and cardiomyocytes to exacerbate anthracycline cardiotoxicity in clinical settings.

## 4 Discussion

The present study generated a number of novel findings. First, we revealed a previously unreported indirect mechanism that sEVs secreted from DOX-exposed breast cancer cells exacerbated anthracycline cardiotoxicity. Second, DOX-treated breast cancer cells (D-BCCs) predispose cardiomyocytes to DOX-induced ferroptosis through transferring sEVs-packaged miR-338-3p. Third, miR-338-3p aggravates DOX-induced cardiomyocytes ferroptosis by targeting CP/SLC7A11/GPX4. Moreover, we also unraveled N^6^-methyladenosine-mediated excessive maturation accounting for the upregulation of miR-338-3p in DOX-treated breast cancer cells and RBMX-mediated sorting and packaging of miR-338-3p into sEVs. Therapeutically, we developed dual-functional decoying sEVs encapsulated with miR-338-3p inhibitor that effectively mitigated DOXIC in an orthotopic breast cancer mouse model. Finally, we revealed that sEVs from chemotherapy patients aggravated cardiomyocytes ferroptosis, which can be reversed by miR-338-3p inhibitor. Together, we provide novel insights into the evolving field of cardio-oncology focusing on organ communication with potential clinical implications.

Apart from the common risk factors shared by cancer and cardiovascular disease, recent studies have revealed potential crosstalk between cancer and heart in these two diseases entities [3]. These interactions can be mediated by either circulating soluble factors as well as sEVs [37]. In patients with heart failure, the circulating level of α1-antichymotrypsin (serpin A3) was elevated and contributed to the progression of colon cancer [8]. The elevated periostin levels in the serum of transverse aortic constriction (TAC)-operated was causatively associated with larger orthotopic breast and lung cancer tumors and a higher rate of metastatic spread [9]. In a more recent study, cardiac mesenchymal stromal cell–derived sEVs from infarcted heart carry cytokines and miRNAs with protumorigenic properties such as TGF-β, galectin-3, and miR-221 to accelerate the growth of lung and colon cancers [38]. Similarly, sEVs containing miR-22-3p secreted from cardiomyocytes increased the vulnerability of osteosarcoma cells to erastin-induced ferroptosis in ischemic heart failure [22]. Our current study revealed a possible role of sEVs-encapsulated pro-ferroptotic miR-338-3p from breast cancer cells in exacerbating DOX-induced cardiomyocytes ferroptosis in the context of doxorubicin-induced cardiac injury, which complemented the previous studies to show that sEVs secreted from tumor cells can also affect the biological behavior of cardiomyocytes.

Ferroptosis, knowns as an iron-dependent form of non-apoptotic cell death prevalent in various cardiovascular diseases and malignancies [39]. The hallmark of ferroptosis is unrestricted iron accumulation and lipid peroxidation. Ferroptosis was reported to mediate both chemotherapy- and ischemia/reperfusion-induced cardiomyopathy, both iron chelation and pharmacologically blocking of ferroptosis therapies could significantly ameliorate cardiomyopathy in mice [40]. Furthermore, the regulation of ferroptosis susceptibility has been implicated in the crosstalk between cardiomyocytes and cancer cells, sEVs from cardiomyocytes sensitized osteosarcoma cells to ferroptosis in ischemic heart failure by transferring miR-22-3p [22]. In our hands, we showed that breast cancer cells predispose cardiomyocytes to DOX-induced ferroptosis through transferring sEVs-packaged miR-338-3p. Reciprocally, our findings revealed that cancer cells can also affect the ferroptosis susceptibility of cardiomyocytes *via* transferring sEVs, which extended our understanding of sEVs mediating the interaction between cancer and heart through regulating ferroptosis susceptibility.

sEVs carry a variety of cargos, including but not limited to multiple RNA species such as miRNAs, lipids and proteins; however, most of the inter-tissue and inter-organ communication actions of sEVs in previous studies have been attributed to their miRNA content [29,41,42]. To elucidate this concept in the present study, we employed a Dicer-KO approach. Dicer is the key protein essential for processing miRNAs and when Dicer is depleted, cells can still make sEVs, but these sEVs contain greatly reduced levels of miRNAs [29]. We found that our KO approach led to successful Dicer KO with a corresponding ∼90 % decrease in miRNA content. Notably, D-BCC-sEVs derived from Dicer-KO cells had no effects on DOXIC, showing that the DOXIC aggravating effects of D-BCC-sEVs can be attributed to their miRNA content.

miRNAs have been recognized as crucial component of sEVs and essential regulators of various biological processes. Our current study showed that miR-338-3p is enriched in sEVs of DOX-treated BCCs and can be transferred to cardiomyocytes to regulate the ferroptosis susceptibility of cardiomyocytes. In accordance with our findings, a sEV miRNA profiling study indicated that miR-338-3p was elevated in the plasma of BC patients with recurrence compared with those without recurrence [43]. Further functional studies unveiled that miR-338-3p served as a tumor suppressor in BC by inhibiting BC cell proliferation, invasion, migration, and epithelial-mesenchymal transition [44,45]. It has been reported that some tumor suppressive miRNAs like miR-320, were enriched in sEVs and thus secreted by tumor cells, resulting in low level of miR-320 and increased proliferation of the donor tumor cells [46]. These findings enlightened us that BCCs may also accelerate its progression by secreting the tumor-suppressive miR-338-3p to sEVs, thus constituting a vicious cycle between BC progression and cardiomyocytes injury. Future studies are warranted to evaluate this assumption.

miRNAs have emerged as crucial regulators of ferroptosis. They usually participate in gene regulation through matching to a site in the 3′ UTR of messenger RNAs (mRNAs). Our research found that the transferred miR-338-3p further targeted anti-ferroptotic genes including Ceruloplasmin (CP), a copper-containing ferroxidase that facilitates the conversion of ferrous iron to ferric iron [47], Solute Carrier Family 7 Member 11 (SLC7A11), a sodium-independent cystine-glutamate antiporter that maintains the intracellular levels of glutathione that is essential for resisting oxidative stress [48], and Glutathione Peroxidase 4 (GPX4), a phospholipid hydroperoxidase that protects cells against membrane lipid peroxidation [49], to accelerate their mRNA degradation and thus fuels ferroptosis. In this study, while all of the SLC7A11, GPX4 and CP 3′-UTR mutations comparably prevented the increased hiCM viability loss, cTnI leakage induced by miR-338-3p transfection, SLC7A11 and GPX4 3′-UTR mutation were more effective in reversing GSH and GSSG, whereas CP1 3′-UTR mutation behaved better in averting ferrous iron accumulation, fitting well with their respective established roles. Moreover, 3′- UTR has been revealed to be effective RNA-based therapy tools for treating cardiac diseases irrespective of its cognate protein [50], the findings in this study suggested that mutated SLC7A11, GPX4 and CP 3′-UTRs may be effective RNA-based therapeutic targets for DOXIC in cancer patients. Notably, a previous study reported that miR-338-3p targeting SLC1A5 in retinal pigment epithelium cells promoted ferroptosis [51]. In our hands, although SLC1A5 was not experimentally established as a target of miR-338-3p in cardiomyocytes, the pro-ferroptotic role of miR-338-3p has been validated, which might constitute an appealing intervention target for diseases caused by aberrant ferroptosis activation.

We have found in this study that RNA-binding protein RBMX was responsible for sorting and packaging miR-338-3p into sEVs of BCCs. The specific association between miR-338-3p and RBMX was further reveal to mediate miR-338-3p sorting and packaging into sEVs of BCCs. Similarly, a previous study revealed that RBMX bound to miR-19b-3p to facilitate the packaging of miR-19b-3p into lung adenocarcinoma cell-derived exosomes [52]. A more recent study showed that de-sumoylated RBMX promoted the exosomal sorting of miR-26a, miR-23c and miR-874 in renal tubular epithelial cells to participate in the pathogenesis of renal tubulointerstitial fibrosis [53]. In this study, we have further validated the potential therapeutic value of this packaging mechanism, RBMX silencing efficiently prevented BCC-elicited DOXIC aggravating effects by blocking the adverse communication between breast cancer cells and cardiomyocytes. This finding might indicate the feasibility of targeting sorting and packaging mechanism in preventing sEVs-mediated pathological communication in the pathogenesis of other diseases associated with pathogenic sEVs transmitting.

In this study, N^6^-methyladenosine-mediated miR-338-3p maturation contributed to its upregulation in DOX-treated BCCs. As the most prevalent and abundant internal post-transcriptional modification, m^6^A played diverse and important roles in RNA metabolism including miRNA processing [30]. Consistent with the previous study, here we found in 231 cells that methyltransferase-like 3(METTL3) methylated pri-miR-338-3p, facilitating it for recognition and processing by DGCR8, thereby promoting the maturation process of miR-338-3p. Furthermore, we have further extended the functional relevance of this mechanism by showing that interfering METTL3 or DGCR8 or the small molecular inhibitor of METTL3, STM2457 alleviated the BCC-elicited DOXIC aggravating effects mediated by D-BCC-sEVs.

Extracellular vesicles have emerged as a next-generation therapy not only due to their capacity to deliver therapeutic agents (proteins, genes, nucleic acids, viruses, and compounds) but also due to their ability to orchestrate complex tasks of tissue regeneration and repair [54]. We have further developed a dual-functional decoying sEVs encapsulated with miR-338-3p inhibitor mitigated DOXIC in an orthotopic breast cancer mouse model. This decoying sEVs were derived from bone marrow mesenchymal stem cells, which has been revealed to be cardioprotective cell-free therapeutics [55]. Moreover, the decoying sEVs were engineered with the modified “G-C” abundant tetrahedral DNA nanostructure (TDN) to capture DOX, and cardiac targeting peptide (CTP) to improve its cardiac targeting ability. By combining sEVs^TDN−CTP^ with miR-338-3p inhibitor, we aimed to target the direct and indirect mechanism of DOXIC that we reported in this study simultaneously and examined whether they have a synergistical effect in alleviating DOXIC in tumor-bearing mice. As expected, this dual-functional decoying sEVs encapsulated with miR-338-3p inhibitor synergistically mitigated DOXIC in an orthotopic breast cancer mouse model, which may have implications for sEVs-based delivery of therapeutic oligonucleotides for cardiac diseases.

Notably, we observed significant cardiac atrophy following the DOX exposure in a subacute animal model. While the ultimate outcome of doxorubicin cardiotoxicity can be heart failure with features of dilated cardiomyopathy (which can involve some degree of “hypertrophy” in the context of chamber dilation as a compensatory mechanism), the direct and subacute effect of doxorubicin on the heart muscle itself is primarily atrophy [56] (a decrease in size and mass of cardiomyocytes).

Our current study suffered from several limitations. First, although our results suggested a significant contribution of breast cancer cells to DOXIC, we acknowledged the need to investigate the role of sEVs from other types of tumors in DOXIC. Secondly, the methods of infusing sEVs in experimental animals or adding them to cell cultures may not fully replicate the natural releasing process of cancerous sEVs. Third, although we revealed the pathological role of sEVs-transmitted miR-338-3p in DOXIC, we cannot exclude the contribution of other molecules such as long-noncoding RNAs or proteins in sEVs and circulating soluble factors such as small molecule metabolites outside sEVs in this process. Besides, the efficacy and safety of blocking sorting and packaging mechanism by silencing RBMX and blocking miR-338-3p maturation by interfering METTL3 or DGCR8 were not evaluated in mice, which warrants further investigations. In addition, despite the current finding that D-BCC-sEVs worsened DOX-induced cardiotoxicity, we cannot exclude the possibility that EVs from endothelial cells, peripheral blood mononuclear cells or cardiac fibroblasts might also contribute to toxicity in cardiomyocytes. Moreover, D-BCC-sEVs might also work in an indirect manner by impacting endothelial cells *in vivo*. We wished to study these issues in the future.

**Fig. 10:**
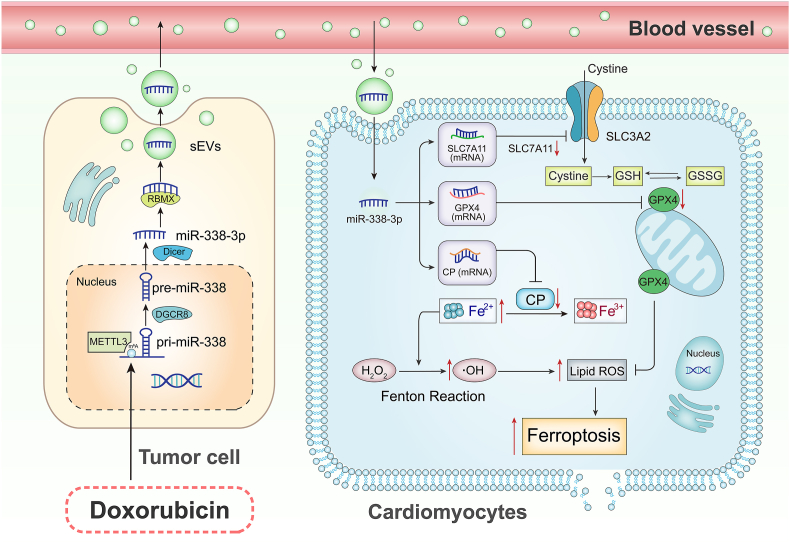
(Graphical abstract). Schematic illustration depicting the working model of this study. In response to DOX, Breast cancer cells (BCCs) can secrete sEVs that are released into the circulation, taken up by cardiomyocytes and participate in the pathogenesis of doxorubicin-induced cardiotoxicity (DOXIC). BCCs sEVs-transmitted miR-338-3p functions as a master regulator orchestrating the pathological communications between breast cancer cells and cardiomyocytes to exacerbate anthracycline cardiotoxicity. The mechanism underlying the detrimental effects of BCCs sEVs-transmitted miR-338-3p in DOXIC is that m^6^A-induced miR-338-3p sorted and packaged by RBMX could be transferred to cardiomyocytes and targeted SLC7A11, CP, and GPX4, thereby inducing ferrous iron accumulation and lipid peroxidation and ultimately predisposing cardiomyocytes to DOX-induced ferroptosis.

In summary, our present work provides both *in vitro* and *in vivo* evidence that BCCs sEVs-transmitted miR-338-3p functions as a master regulator orchestrating the pathological communications between breast cancer cells and cardiomyocytes to exacerbate anthracycline cardiotoxicity. The mechanism underlying the detrimental effects of BCCs sEVs-transmitted miR-338-3p in DOXIC is that m^6^A-induced miR-338-3p sorted and packaged by RBMX could be transferred to cardiomyocytes and predispose cardiomyocytes to DOX-induced ferroptosis by targeting SLC7A11, CP, and GPX4 (Fig. 10). Targeted inhibition of abnormal BC-sEV releasing by Rab27a silence in breast cancer tissue abrogated BCC-induced DOXIC aggravating effects. More importantly, dual-functional decoying sEVs encapsulated with miR-338-3p inhibitor have a synergistical effect in alleviating DOXIC in tumor-bearing mice. Our findings not only provide new insights into the pathological communications between breast cancer cells and cardiomyocytes in DOXIC, but may also have important implications for the development of novel strategies to treat patients with tumor and cardiovascular comorbidities.

## CRediT authorship contribution statement

**Dong Han:** Writing – review & editing, Writing – original draft, Visualization, Validation, Supervision, Resources, Project administration, Methodology, Investigation, Funding acquisition, Formal analysis, Data curation, Conceptualization. **Tianhu Wang:** Writing – original draft, Software, Methodology, Investigation, Data curation. **Xiaoyao Li:** Validation, Resources, Project administration, Investigation, Data curation. **Cheng Qin:** Writing – review & editing, Visualization, Validation, Supervision, Investigation, Formal analysis. **Yingjie Zhang:** Writing – original draft, Visualization, Validation, Software, Methodology. **Tingwen Zhou:** Visualization, Methodology, Investigation, Formal analysis, Data curation. **Shan Gao:** Validation, Software, Resources, Methodology. **Weiwei Zhang:** Supervision, Methodology, Investigation, Funding acquisition, Data curation. **Yongjun Wang:** Writing – review & editing, Visualization, Validation, Supervision, Resources, Project administration, Methodology, Investigation, Data curation, Conceptualization. **Yan Ma:** Writing – original draft, Visualization, Validation, Software, Methodology, Investigation, Data curation, Conceptualization. **Feng Cao:** Writing – review & editing, Supervision, Software, Resources, Project administration, Investigation, Funding acquisition, Formal analysis, Data curation, Conceptualization.

## Disclosures

None.

## Sources of funding

This work was supported by the 10.13039/501100001809National Natural Science Foundation of China (No. 82470366, 82100372), the 10.13039/501100012166National Key Research and Development Program of China (No. 2022YFA1104704), and Beijing Natural Science Foundation (7232159).

## Declaration of competing interest

The authors declare that they have no known competing financial interests or personal relationships that could have appeared to influence the work reported in this paper.

## Data Availability

Data will be made available on request.

## References

[1] Curigliano G., Cardinale D., Dent S., Criscitiello C., Aseyev O., Lenihan D., Cipolla C.M. (2016). Cardiotoxicity of anticancer treatments: epidemiology, detection, and management. CA Cancer J. Clin..

[2] Ahmad A. (2019). Breast cancer statistics: recent trends. Adv. Exp. Med. Biol..

[3] Lopez-Fernandez T., Marco I., Aznar M.C., Barac A., Bergler-Klein J., Meattini I., Scott J.M., Cardinale D., Dent S. (2024). Breast cancer and cardiovascular health. Eur. Heart J..

[4] Siegel R.L., Miller K.D., Jemal A. (2015). Cancer statistics, 2015. CA Cancer J. Clin..

[5] Herrmann J. (2020). Adverse cardiac effects of cancer therapies: cardiotoxicity and arrhythmia. Nat. Rev. Cardiol..

[6] de Wit S., Glen C., de Boer R.A., Lang N.N. (2023). Mechanisms shared between cancer, heart failure, and targeted anti-cancer therapies. Cardiovasc. Res..

[7] Hasin T., Gerber Y., McNallan S.M., Weston S.A., Kushwaha S.S., Nelson T.J., Cerhan J.R., Roger V.L. (2013). Patients with heart failure have an increased risk of incident cancer. J. Am. Coll. Cardiol..

[8] Meijers W.C., Maglione M., Bakker S.J.L., Oberhuber R., Kieneker L.M., de Jong S., Haubner B.J., Nagengast W.B., Lyon A.R., van der Vegt B., van Veldhuisen D.J., Westenbrink B.D., van der Meer P., Sillje H.H.W., de Boer R.A. (2018). Heart failure stimulates tumor growth by circulating factors. Circulation.

[9] Avraham S., Abu-Sharki S., Shofti R., Haas T., Korin B., Kalfon R., Friedman T., Shiran A., Saliba W., Shaked Y., Aronheim A. (2020). Early cardiac remodeling promotes tumor growth and metastasis. Circulation.

[10] Armenian S.H., Xu L., Ky B., Sun C., Farol L.T., Pal S.K., Douglas P.S., Bhatia S., Chao C. (2016). Cardiovascular disease among survivors of adult-onset cancer: a community-based retrospective cohort study. J. Clin. Oncol..

[11] Cosper P.F., Leinwand L.A. (2011). Cancer causes cardiac atrophy and autophagy in a sexually dimorphic manner. Cancer Res..

[12] da Costa T.S.R., Urias U., Negrao M.V., Jordao C.P., Passos C.S., Gomes-Santos I.L., Salemi V.M.C., Camargo A.A., Brum P.C., Oliveira E.M., Hajjar L.A., Chammas R., Filho R.K., Negrao C.E. (2021). Breast cancer promotes cardiac dysfunction through deregulation of cardiomyocyte Ca(2+)-Handling protein expression that is not reversed by exercise training. J. Am. Heart Assoc..

[13] Fu S., Zhang Y., Li Y., Luo L., Zhao Y., Yao Y. (2020). Extracellular vesicles in cardiovascular diseases. Cell Death Discov..

[14] Gabisonia K., Khan M., Recchia F.A. (2022). Extracellular vesicle-mediated bidirectional communication between heart and other organs. Am. J. Physiol. Heart Circ. Physiol..

[15] Gan L., Xie D., Liu J., Bond Lau W., Christopher T.A., Lopez B., Zhang L., Gao E., Koch W., Ma X.L., Wang Y. (2020). Small extracellular microvesicles mediated pathological communications between dysfunctional adipocytes and cardiomyocytes as a novel mechanism exacerbating ischemia/reperfusion injury in Diabetic mice. Circulation.

[16] Gan L., Liu D., Xie D., Bond Lau W., Liu J., Christopher T.A., Lopez B., Liu L., Hu H., Yao P., He Y., Gao E., Koch W.J., Zhao J., Ma X.L., Cao Y., Wang Y. (2022). Ischemic heart-derived small extracellular vesicles impair adipocyte function. Circ. Res..

[17] Zhao H., Chen X., Hu G., Li C., Guo L., Zhang L., Sun F., Xia Y., Yan W., Cui Z., Guo Y., Guo X., Huang C., Fan M., Wang S., Zhang F., Tao L. (2022). Small extracellular vesicles from brown adipose tissue mediate exercise cardioprotection. Circ. Res..

[18] Murach K.A., McCarthy J.J. (2017). MicroRNAs, heart failure, and aging: potential interactions with skeletal muscle. Heart Fail. Rev..

[19] Wang B., Zhang A., Wang H., Klein J.D., Tan L., Wang Z.M., Du J., Naqvi N., Liu B.C., Wang X.H. (2019). miR-26a limits muscle wasting and cardiac fibrosis through exosome-mediated microRNA transfer in chronic kidney disease. Theranostics.

[20] Cheng M., Yang J., Zhao X., Zhang E., Zeng Q., Yu Y., Yang L., Wu B., Yi G., Mao X., Huang K., Dong N., Xie M., Limdi N.A., Prabhu S.D., Zhang J., Qin G. (2019). Circulating myocardial microRNAs from infarcted hearts are carried in exosomes and mobilise bone marrow progenitor cells. Nat. Commun..

[21] Sun R., Liu W., Zhao Y., Chen H., Wang Z., Zhang Y., Sun X., Cui X. (2021). Exosomal circRNA as a novel potential therapeutic target for multiple myeloma-related myocardial damage. Cancer Cell Int..

[22] Yuan Y., Mei Z., Qu Z., Li G., Yu S., Liu Y., Liu K., Shen Z., Pu J., Wang Y., Wang C., Sun Z., Liu Q., Pang X., Wang A., Ren Z., Wang T., Liu Y., Hong J., Xie J., Li X., Wang Z., Du W., Yang B. (2023). Exosomes secreted from cardiomyocytes suppress the sensitivity of tumor ferroptosis in ischemic heart failure. Signal Transduct. Targeted Ther..

[23] Welsh J.A., Goberdhan D.C.I., O'Driscoll L., Buzas E.I., Blenkiron C., Bussolati B., Cai H., Di Vizio D., Driedonks T.A.P., Erdbrugger U., Falcon-Perez J.M., Fu Q.L., Hill A.F., Lenassi M., Lim S.K., Mahoney M.G., Mohanty S., Moller A., Nieuwland R., Ochiya T., Sahoo S., Torrecilhas A.C., Zheng L., Zijlstra A., Abuelreich S., Bagabas R., Bergese P., Bridges E.M., Brucale M., Burger D., Carney R.P., Cocucci E., Crescitelli R., Hanser E., Harris A.L., Haughey N.J., Hendrix A., Ivanov A.R., Jovanovic-Talisman T., Kruh-Garcia N.A., Ku'ulei-Lyn Faustino V., Kyburz D., Lasser C., Lennon K.M., Lotvall J., Maddox A.L., Martens-Uzunova E.S., Mizenko R.R., Newman L.A., Ridolfi A., Rohde E., Rojalin T., Rowland A., Saftics A., Sandau U.S., Saugstad J.A., Shekari F., Swift S., Ter-Ovanesyan D., Tosar J.P., Useckaite Z., Valle F., Varga Z., van der Pol E., van Herwijnen M.J.C., Wauben M.H.M., Wehman A.M., Williams S., Zendrini A., Zimmerman A.J., Consortium M., Thery C., Witwer K.W. (2024). Minimal information for studies of extracellular vesicles (MISEV2023): from basic to advanced approaches. J. Extracell. Vesicles.

[24] Fang X., Ardehali H., Min J., Wang F. (2023). The molecular and metabolic landscape of iron and ferroptosis in cardiovascular disease. Nat. Rev. Cardiol..

[25] Chen F., Chen J., Yang L., Liu J., Zhang X., Zhang Y., Tu Q., Yin D., Lin D., Wong P.P., Huang D., Xing Y., Zhao J., Li M., Liu Q., Su F., Su S., Song E. (2019). Extracellular vesicle-packaged HIF-1alpha-stabilizing lncRNA from tumour-associated macrophages regulates aerobic glycolysis of breast cancer cells. Nat. Cell Biol..

[26] Yang M.Q., Du Q., Goswami J., Varley P.R., Chen B., Wang R.H., Morelli A.E., Stolz D.B., Billiar T.R., Li J., Geller D.A. (2018). Interferon regulatory factor 1-Rab27a regulated extracellular vesicles promote liver ischemia/reperfusion injury. Hepatology.

[27] Wills C.A., Liu X., Chen L., Zhao Y., Dower C.M., Sundstrom J., Wang H.G. (2021). Chemotherapy-induced upregulation of small extracellular vesicle-associated PTX3 accelerates breast cancer metastasis. Cancer Res..

[28] Jiang Y., Glandorff C., Sun M. (2024). GSH and ferroptosis: side-By-side partners in the fight against tumors. Antioxidants.

[29] Ying W., Gao H., Dos Reis F.C.G., Bandyopadhyay G., Ofrecio J.M., Luo Z., Ji Y., Jin Z., Ly C., Olefsky J.M. (2021). MiR-690, an exosomal-derived miRNA from M2-polarized macrophages, improves insulin sensitivity in obese mice. Cell Metab..

[30] Alarcon C.R., Lee H., Goodarzi H., Halberg N., Tavazoie S.F. (2015). N6-methyladenosine marks primary microRNAs for processing. Nature.

[31] Fan R., Cui C., Kang B., Chang Z., Wang G., Cui Q. (2024). A combined deep learning framework for mammalian m6A site prediction. Cell Genom.

[32] Xiao Y., Wang Y., Tang Q., Wei L., Zhang X., Jia G. (2018). An Elongation- and ligation-based qPCR amplification method for the radiolabeling-free detection of locus-specific N(6) -Methyladenosine modification. Angew Chem. Int. Ed. Engl..

[33] Fabbiano F., Corsi J., Gurrieri E., Trevisan C., Notarangelo M., D'Agostino V.G. (2020). RNA packaging into extracellular vesicles: an orchestra of RNA-binding proteins?. J. Extracell. Vesicles.

[34] Cook K.B., Kazan H., Zuberi K., Morris Q., Hughes T.R. (2011). RBPDB: a database of RNA-binding specificities. Nucleic Acids Res..

[35] Fan M., Li H., Shen D., Wang Z., Liu H., Zhu D., Wang Z., Li L., Popowski K.D., Ou C., Zhang K., Zhang J., Cheng K., Li Z. (2022). Decoy exosomes offer protection against chemotherapy-induced toxicity. Adv. Sci. (Weinh.).

[36] Zhang Z., Liang D., Gao X., Zhao C., Qin X., Xu Y., Su T., Sun D., Li W., Wang H., Liu B., Cao F. (2014). Selective inhibition of inositol hexakisphosphate kinases (IP6Ks) enhances mesenchymal stem cell engraftment and improves therapeutic efficacy for myocardial infarction. Basic Res. Cardiol..

[37] Badila E., Japie C., Vrabie A.-M., Badila A., Georgescu A. (2023). Cardiovascular disease as a consequence or a cause of cancer: potential role of extracellular vesicles. Biomolecules.

[38] Caller T., Rotem I., Shaihov-Teper O., Lendengolts D., Schary Y., Shai R., Glick-Saar E., Dominissini D., Motiei M., Katzir I., Popovtzer R., Nahmoud M., Boomgarden A., D'Souza-Schorey C., Naftali-Shani N., Leor J. (2024). Small extracellular vesicles from infarcted and failing heart accelerate tumor growth. Circulation.

[39] Berndt C., Alborzinia H., Amen V.S., Ayton S., Barayeu U., Bartelt A., Bayir H., Bebber C.M., Birsoy K., Bottcher J.P., Brabletz S., Brabletz T., Brown A.R., Brune B., Bulli G., Bruneau A., Chen Q., DeNicola G.M., Dick T.P., Distefano A., Dixon S.J., Engler J.B., Esser-von Bieren J., Fedorova M., Friedmann Angeli J.P., Friese M.A., Fuhrmann D.C., Garcia-Saez A.J., Garbowicz K., Gotz M., Gu W., Hammerich L., Hassannia B., Jiang X., Jeridi A., Kang Y.P., Kagan V.E., Konrad D.B., Kotschi S., Lei P., Le Tertre M., Lev S., Liang D., Linkermann A., Lohr C., Lorenz S., Luedde T., Methner A., Michalke B., Milton A.V., Min J., Mishima E., Muller S., Motohashi H., Muckenthaler M.U., Murakami S., Olzmann J.A., Pagnussat G., Pan Z., Papagiannakopoulos T., Pedrera Puentes L., Pratt D.A., Proneth B., Ramsauer L., Rodriguez R., Saito Y., Schmidt F., Schmitt C., Schulze A., Schwab A., Schwantes A., Soula M., Spitzlberger B., Stockwell B.R., Thewes L., Thorn-Seshold O., Toyokuni S., Tonnus W., Trumpp A., Vandenabeele P., Vanden Berghe T., Venkataramani V., Vogel F.C.E., von Karstedt S., Wang F., Westermann F., Wientjens C., Wilhelm C., Wolk M., Wu K., Yang X., Yu F., Zou Y., Conrad M. (2024). Ferroptosis in health and disease. Redox Biol..

[40] Fang X., Wang H., Han D., Xie E., Yang X., Wei J., Gu S., Gao F., Zhu N., Yin X., Cheng Q., Zhang P., Dai W., Chen J., Yang F., Yang H.T., Linkermann A., Gu W., Min J., Wang F. (2019). Ferroptosis as a target for protection against cardiomyopathy. Proc Natl Acad Sci U S A..

[41] Rohm T.V., Castellani Gomes Dos Reis F., Isaac R., Murphy C., Cunha E.R.K., Bandyopadhyay G., Gao H., Libster A.M., Zapata R.C., Lee Y.S., Ying W., Miciano C., Wang A., Olefsky J.M. (2024). Adipose tissue macrophages secrete small extracellular vesicles that mediate rosiglitazone-induced insulin sensitization. Nat. Metab..

[42] Cao M., Isaac R., Yan W., Ruan X., Jiang L., Wan Y., Wang J., Wang E., Caron C., Neben S., Drygin D., Pizzo D.P., Wu X., Liu X., Chin A.R., Fong M.Y., Gao Z., Guo K., Fadare O., Schwab R.B., Yuan Y., Yost S.E., Mortimer J., Zhong W., Ying W., Bui J.D., Sears D.D., Olefsky J.M., Wang S.E. (2022). Cancer-cell-secreted extracellular vesicles suppress insulin secretion through miR-122 to impair systemic glucose homeostasis and contribute to tumour growth. Nat. Cell Biol..

[43] Sueta A., Yamamoto Y., Tomiguchi M., Takeshita T., Yamamoto-Ibusuki M., Iwase H. (2017). Differential expression of exosomal miRNAs between breast cancer patients with and without recurrence. Oncotarget.

[44] Liang Y., Xu X., Wang T., Li Y., You W., Fu J., Liu Y., Jin S., Ji Q., Zhao W., Song Q., Li L., Hong T., Huang J., Lyu Z., Ye Q. (2017). The EGFR/miR-338-3p/EYA2 axis controls breast tumor growth and lung metastasis. Cell Death Dis..

[45] He J., Wang J., Li S., Li T., Chen K., Zhang S. (2020). Hypoxia-inhibited miR-338-3p suppresses breast cancer progression by directly targeting ZEB2. Cancer Sci..

[46] Gao X., Wan Z., Wei M., Dong Y., Zhao Y., Chen X., Li Z., Qin W., Yang G., Liu L. (2019). Chronic myelogenous leukemia cells remodel the bone marrow niche via exosome-mediated transfer of miR-320. Theranostics.

[47] Schwantes A., Wickert A., Becker S., Baer P.C., Weigert A., Brune B., Fuhrmann D.C. (2024). Tumor associated macrophages transfer ceruloplasmin mRNA to fibrosarcoma cells and protect them from ferroptosis. Redox Biol..

[48] Yan Y., Teng H., Hang Q., Kondiparthi L., Lei G., Horbath A., Liu X., Mao C., Wu S., Zhuang L., James You M., Poyurovsky M.V., Ma L., Olszewski K., Gan B. (2023). SLC7A11 expression level dictates differential responses to oxidative stress in cancer cells. Nat. Commun..

[49] Seibt T.M., Proneth B., Conrad M. (2019). Role of GPX4 in ferroptosis and its pharmacological implication. Free Radic. Biol. Med..

[50] Zhao Y., Ling S., Li J., Zhong G., Du R., Li Y., Wang Y., Liu C., Jin X., Liu W., Liu T., Li Y., Zhao D., Sun W., Liu Z., Liu Z., Pan J., Yuan X., Gao X., Xing W., Chang Y.Z., Li Y. (2021). 3' untranslated region of Ckip-1 inhibits cardiac hypertrophy independently of its cognate protein. Eur. Heart J..

[51] Zhou J., Sun C., Dong X., Wang H. (2022). A novel miR-338-3p/SLC1A5 axis reprograms retinal pigment epithelium to increases its resistance to high glucose-induced cell ferroptosis. J. Mol. Histol..

[52] Chen J., Zhang K., Zhi Y., Wu Y., Chen B., Bai J., Wang X. (2021). Tumor-derived exosomal miR-19b-3p facilitates M2 macrophage polarization and exosomal LINC00273 secretion to promote lung adenocarcinoma metastasis via hippo pathway. Clin. Transl. Med..

[53] Yang Y., Ren S., Xue J., Dong W., He W., Luo J., Li X., Xu H., Zheng Z., Wang X., Wang L., Guan M., Jia Y., Xue Y. (2025). DeSUMOylation of RBMX regulates exosomal sorting of cargo to promote renal tubulointerstitial fibrosis in diabetic kidney disease. J. Adv. Res..

[54] Rai A., Claridge B., Lozano J., Greening D.W. (2024). The discovery of extracellular vesicles and their emergence as a next-generation therapy. Circ. Res..

[55] Lei B., Wu X., Xia K., Sun H., Wang J. (2021). Exosomal Micro-RNA-96 derived from bone marrow mesenchymal stem cells inhibits doxorubicin-induced myocardial toxicity by inhibiting the Rac1/Nuclear Factor-kappaB signaling pathway. J. Am. Heart Assoc..

[56] Willis M.S., Parry T.L., Brown D.I., Mota R.I., Huang W., Beak J.Y., Sola M., Zhou C., Hicks S.T., Caughey M.C., D'Agostino R.B., Jordan J., Hundley W.G., Jensen B.C. (2019). Doxorubicin exposure causes subacute cardiac atrophy dependent on the striated muscle-specific ubiquitin ligase MuRF1. Circ Heart Fail.

