# Supplementary material for: Small extracellular vesicles orchestrated pathological communications between breast cancer cells and cardiomyocytes as a novel mechanism exacerbating anthracycline cardiotoxicity by fueling ferroptosis
Source: Redox Biol. 2025 Sep 3;86:103843. doi: 10.1016/j.redox.2025.103843 (PMC12446661; doi:10.1016/j.redox.2025.103843)
Supplement: Multimedia component 1 [file mmc1.docx]

Supplemental Material

**Small extracellular vesicles** **orchestrated** **pathological communications between** **breast cancer cells and cardiomyocytes as a novel mechanism exacerbating anthracycline cardiotoxicity by** **fueling ferroptosis**

*Dong Han^1^*, Tianhu Wang^1^, Xiaoyao Li^2^, Cheng Qin^1^, Yingjie Zhang^1^, Tingwen Zhou^3^, Shan Gao^1^, Weiwei Zhang^1^, Yongjun Wang^3^, Yan Ma ^1*^, Feng Cao ^1, 4^*

^1^National Clinical Research Center for Geriatric Diseases, the Second Medical Center, Chinese PLA General Hospital, 100853, Beijing, China

^2^Arrhythmia Center, State Key Laboratory of Cardiovascular Disease, Fuwai Hospital, National Center for Cardiovascular Diseases, Chinese Academy of Medical Sciences and Peking Union Medical College, Beijing, China

^3^Department of Cardiovascular Surgery, Union Hospital, Tongji Medical College, Huazhong University of Science and Technology, 1277# Jiefang Avenue, Wuhan, Hubei 430022, China

^4^Institute of Geriatric Medicine, the Second Medical Center, Chinese PLA General Hospital, 100853, Beijing, China

Running head of the title: sEVs exacerbate DOXIC

*Corresponding author:

Dong Han, M.D., Ph.D. National Clinical Research Center for Geriatric Diseases, the Second Medical Center, Chinese PLA General Hospital, 100853, Beijing, China; [handong123566@163.com](mailto:handong123566@163.com) (D.H.)

Yan Ma, M.D., Ph.D. National Clinical Research Center for Geriatric Diseases, the Second Medical Center, Chinese PLA General Hospital, 100853, Beijing, China; aslmalkum@qq.com (Y.M.)

**Supplemental Material**

Supplemental Methods

Graphical abstract

Figures S1–S12

Tables S1–S4

Supplemental Methods

**Supplementary Methods**

**Orthotopic breast cancer (BC) mice model**

The animal study was approved by the Ethics Committee of the People's Liberation Army General Hospital (S2021-378-02). The orthotopic breast cancer (BC) mice model was constructed as previously described^23^. Briefly, the syngeneic murine mammary cancer cell line E0771 (CRL-3461, ATCC) cells with a number of 5×10^5^ resuspended in 100 µl calcium- and magnesium-free PBS were seeded into the into the left fifth inguinal mammary fat pad of C57BL/6J female mice aged 8-10 weeks old. The tumor volume was measured with a vernier caliper after the appearance of the tumor. The calculation formula of tumor volume is V = (shortest diameter)^2^ × (longest diameter) × 0.5. For doxorubicin (DOX) exposure experiments, mice received intraperitoneal injection of 5 mg/kg DOX, or an equivalent volume of PBS starting from day 7 and every one week after E0771 cells inoculation (a total of 5 doses). Non-invasive transthoracic echocardiography was performed to evaluate cardiac function.

**Intertumoral injection of shRab27a**

In the present study, we employed intratumoral injection of Rab27a shRNA‑expressing lentiviral vector to study the effects of blocking the secretion of BCCs sEVs on DOX-induced myocardial injury. ShRNA‑expressing lentiviral vector carrying the short hairpin Rab27a (shRab27a) or negative control (shNC) were designed and constructed by the Hanbio Biotechnology (Shanghai, China). Orthotopic breast cancer (BC) mice received an intratumoral injection of 50 μl (1 × 10^8^ IU/ml) of virus containing ShNC, or virus containing ShRab27a on day 8 and day 22 (the second day after the first and the third dose of DOX, respectively) post E0771 cells inoculation.

**Systemic delivery of D-BCC-sEVs** **through tail-vein injection**

To explore the effect of sEVs isolated from doxorubicin (DOX)-treated breast cancer cells (D-BCC-sEVs) on DOX-induced myocardial injury, sEVs isolated from DOX-treated (1 μM, 24h) or vehicle-treated (0.1% DMSO) E0771 cells were harvested. C57BL/6J female mice receiving DOX treatment were randomly divided into tail vein-injected non targeting D-BCC-sEVs group or N-BCC-sEVs group (vehicle-treated BCC-sEVs). Mice received tail-vein injections of 100 μg sEVs (diluted in 150μL PBS), or an equivalent volume of PBS, on day 8 and day 22 (the second day after the second and the fourth dose of DOX, respectively) post E0771 cells inoculation.

**sEVs^TDN-CTP^-338-inhitor preparation and deliver**

To explore the effects of mesenchymal stem cell sEVs engineered with “G-C” abundant tetrahedral DNA nanostructure (TDN), a cardiac-targeting peptide (CTP), and miR-338-3p inhibitor (sEVs^TDN-CTP^-338inh) on DOX-induced myocardial injury, we synthesized tetrahedral DNA with CTP engineered sEVs (sEVs^TDN-CTP^) by referring to the instructions of a previous study^24^ and loaded with miR-338-3p inhibitor. Bone Marrow Mesenchymal Stem Cells (BMSCs) sEVs were isolated using ultracentrifugation process (see “sEVs isolation and characterization” section).

The synthesis of DNA with tetrahedral DNA nanostructure was performed as previously described^24, 25^. Briefly, S1-cho-two strands (S1-cho and S2-cho), three strands (S1-cho, S2-cho and S3-cho), and four strands (S1-cho, S2-cho, S3-cho and S4) in equimolar amounts was mixed in a buffer (2 × 10^−2^ M Tris and 1.6 × 10^−2^ M MgCl2 at a pH of 8.0) then kept at 95 °C for 10 min, then slowly cooled to 4 °C. The sequence of each single strand of DNA has been listed in Supplementary Table Ⅲ. Four-stranded strands were further reacted to obtain TDN. TDNs can be stored at 4 °C for ≤1 month.

The preparation of sEVs-TDN was performed as previously described^24^. Briefly, a total of 10 μg of sEVs was taken and dispersed into PBS, after which 30 μg of the synthetic tetrahedral DNA was added. The samples were incubated at 37 °C for 1h and then quickly put on ice and shaken for 1 h. Then, a 100 KDa ultrafiltration tube was used to centrifuge the samples at 4500 × g for 15 min to remove excess tetrahedral DNA, after which MSC sEVs engineered with tetrahedral DNA (sEVs^TDN^) were obtained.

The preparation of sEVs^CTP^ was performed as previously described^26^. 1,2-dioleoyl-sn-glycero-3-phosphoethanolamine-N-(polyethy-lene-glycol)-succinyl-N hydroxysuccinimidyl ester (DOPE-PEG-NHS) was purchased from Ruixibiotech (Xi’an, China). Briefly, 20 μg of streptomycin (SA) was incubated with 300 μg of DOPE-PEG-NHS solution in dimethyl sulfoxide at 37 °C for 4 h, and the SA was coupled to the NHS end. Next, peptides (30 μg) of biotinylated cardiac-targeting peptide (CTP, APWHLSSQYSRT) were added, and the mixture was incubated for another 24 h. The reaction was terminated with Tris-HCl buffer (1 M, pH 8.0). Finally, MSC-sEVs (200 μg) were added to the above mixture and incubated at 37 °C for 4 h to obtain sEVs^CTP^.

The loading of miRNA inhibitors into sEVs was performed as previously described^27^. miR-338-3p inhibitor or its negative control (NC) inhibitor modified with 2′-O-methyl and conjugated with cholesterol at the 3’ end were synthesized by (GenePharma, Shanghai). To load miRNA inhibitors into sEVs, these synthesized inhibitors (1 nmol) were incubated with 100 μg/mL sEVs at 37℃ for 1h. Next, unloaded miRNA inhibitors attaching to the sEVs were washed with PBS using Amicon® Ultra Centrifugal Filters (UFC8010, Merck Millipore). To calculate the concentration of miRNA inhibitors loaded onto the sEVs, FAM-labelled and cholesterol-conjugated inhibitors were synthesized and loaded onto the sEVs. After being solubilized using 0.5% Triton X-100, the sEVs were subjected to fluorescence analysis. A standard curve of free FAM-labelled NC inhibitor was plotted to estimate the miRNA inhibitors concentration in the sEVs.

For sEVs^TDN-CTP^-338inh delivery, orthotopic breast cancer (BC) mice receiving DOX chemotherapy were randomly divided into tail vein-injected sEVs^TDN-CTP^-338-inh group, sEVs^CTP^-338-inh or sEVs^TDN-CTP^-NC-inh group. Mice received tail-vein injections of 100 μg modified sEVs (diluted in 150μL PBS), or an equivalent volume of PBS, on day 15 and day 22 (the second day after the second and the fourth dose of DOX, respectively) after E0771 cells inoculation.

**Biodistribution of DiR-labeled sEVs**

The synthesized sEVs^TDN-CTP^ was incubated with 20 mM DiR (Umibio, UR21017). at room temperature for 15 min and then put through a 100KDa ultrafiltration tube at 4 °C. After centrifugation at 4500× g to remove the excess dye, the obtained DiR-labeled sEVs^TDN-CTP^ was resuspended in PBS. After tail-vein injections of 200 μg modified sEVs (diluted in 150μL PBS) for 4 hours, the mice were sacrificed and major organs including the hearts, livers, spleens, lungs, kidneys of mice were removed, and the enrichment of sEVs^TDN-CTP^ in different organs was observed with IVIS Spectrum system (Caliper, USA).

**Agarose electrophoresis**

For the agarose electrophoresis of synthesized TDN, 10 uL of the each synthesized DNA , sEVs-TDN or freshly extracted sEVs was taken, into which 2 uL 6× DNA loading buffer was added. The samples were mixed well and then loaded into the 3% agarose gel well, where they were run at 90 V for 30 min. A gel imager (GelDoc Go Gel Imaging System, Bio-Rad) was used to image the gel.

**The study of DOX uptake into the nuclei**

The hiCMs were seeded onto 30 mm glass-bottom culture dish at a density of 1x10^4^ cells/well. After the cells adhered to the well, 1 uM of Biotin-doxorubicin (Med Chem Express, HY-152965) was added, followed by 24 h of incubation. The original medium was aspirated and cells were washed with PBS three times, after that, the cells were fixed with 4% paraformaldehyde, incubated with 0.1% Triton-X100 and stained with anti-biotin antibody (Abcam, ab53494) followed by the immunofluorescence secondary antibody. Negative controls were incubated overnight with PBS (not primary antibody), and stained with the secondary antibody. Concentration-matched rabbit IgG Isotype control was also employed to serve as negative controls. Nuclei were stained with DAPI. Images were taken by a laser scanning confocal microscopy (LSM710, Zeiss).

**Measurement of mouse cardiac function**

Mouse cardiac function was measured using non-invasive transthoracic echocardiography. Briefly, the mouse inhalation isoflurane anesthesia machine was used to anesthetize. The parameters were set as the oxygen flow rate of 1 L/min and isoflurane concentration of 1.5%. Visual Sonics Vevo 2100 machine with a 30 MHz high-frequency transducer was used to perform cardiac function tests in mice. We recorded M-mode 2D images in parasternal long-axis view. The VEVO computer algorithm was used to calculate left ventricular ejection fraction (LVEF) and left ventricular short-axis shortening (LVFS). We also measured and recorded the late (atrial) diastolic transmitral flow velocity (A), late (atrial) diastolic mitral annular velocity (a’), early diastolic transmitral flow velocity (E), and early diastolic mitral annular velocity (E’) with the color Doppler images of the mitral valve under the color Doppler flow imaging. The VEVO computer algorithm was used to calculate the E/A ratio and the E/E’ ratio.

**Histological analysis**

Samples were fixed in 4% paraformaldehyde, embedded in paraffin, and cut cross-sectionally into 3.0–5.0 µm thick sections. Histological examination was performed with Hematoxylin and Eosin (HE) staining. Additionally, Sirius Red staining was used to identify collagen fibers. Cardiac atrophy was examined by Alexa Fluor 488 labeled wheat germ agglutinin (Invitrogen) staining. Images were taken with Nikon Eclipse Ti microscope and images were analyzed with Image-Pro Plus 6.0 (NIH, Bethesda, MD, USA). For each heart six-ten different images were taken from different regions of the heart section and nearly two hundred to six hundred cells (depending upon the availability) area were measured and the average value was taken for each heart. For Sirius Red staining fibrosis, the fibrosis value was determined by the percentage of positive staining area in whole left ventricle area in each heart section.

**Mouse serum level of cardiac enzymes**

After echocardiography, mice were sacrificed and the blood samples were collected and left to stand at room temperature for 20 min and followed by centrifugation at 2000 g for 20 min to obtain mouse serum. Serum levels of Creatine kinase-MB (CK-MB) and Lactate Dehydrogenase (LDH) levels were measured by ELISA kits following the manufacturers’ instructions (Abcam, Cambridge, MA, USA).

**Cell lines and cell culture**

Human iPSC-derived cardiomyocytes (hiCMs) were chemically induced and cultured as we previously described^28^. Beating cardiomyocytes were maintained in Gibco™ RPMI 1640 medium supplemented with B27 (Thermo Fisher Scientific, USA).

MDA-MB-231 (HTB-26) and E0771 (CRL-3461) breast cancer cells were obtained from American Type Culture Collection and culture in DMEM medium (Gibco) with 10% fetal bovine serum (FBS; Gibco) and RPMI-1640 medium (HyClone, supplemented with 10 mmol/l HEPES), respectively.

Human embryonic kidney cell line (293T) was purchased from the Cell Bank of the Chinese Academy of Sciences (Shanghai, China) and cultured in Dulbecco’s modified Eagle’s medium (DMEM) (Gibco, USA) containing 10% FBS (Gibco, USA).

Adult mouse ventricular myocytes (AMVCs) were prepared from the ventricles of wildtype C57BL/6J wild-type mice aged 6-8 weeks, following a simplified, Langendorff-free method in accordance with previously described procedures^29^. AMVCs were cultured in Dulbecco's Modified Eagle Medium F12 (DMEM/F12, Gibco) supplemented with 0.1% penicillin/streptomycin.

Murine bone marrow mesenchymal stem cells (BMSCs) were isolated from wildtype adult C57BL/6J mice and identified as we previously reported^30, 31^. Briefly, bone marrow was flushed from the femur and tibia of and cultured with fetal bovine serum (FBS) (Gibco, USA)-free DMEM (Gibco, USA). After passing through a 70-μm strainer and centrifugation at 1200 rpm for 5 min at room temperature, the cell pellet was resuspended in DMEM supplemented with 20% FBS and cultured at 37 °C in an incubator containing 5% CO2. After 24 h, the medium was replaced to remove the non-adherent cells and then was completely replaced every 3 days. Third-passage BM-MSCs with optimal growth at the third generation were used for sEV isolation.

AC16 human cardiomyocyte cell line was obtained from Sigma-Aldrich (SCC109). AC16 were maintained in DMEM/F12 with 10% fetal bovine serum (FBS; Gibco), 1 % penicillin/streptomycin (100 U/ml; 100 µg/ml; Sigma Aldrich).

Co-culture of the two cell types was achieved by Transwell inserts (Corning), with MDA-MB-231 or E0771 breast cancer cells in the upper layer and hiCMs or AMVCs in the lower layer. The 0.4 μm membrane between the layers allowed sEVs to pass freely. All cells were randomly allocated into various experimental groups. For doxorubicin treatment, cells were treated with 1μM doxorubicin (Sigma-Aldrich) for 24 hours, the dose and treatment period of doxorubicin were based on our previous study^28^.

All cells used in this study were confirmed to be free of mycoplasma contamination by using the short tandem repeat profiling method. Mycoplasma detection was performed by PCR primer: 5’-GGGAGCAAACAGGATTAGATACCCT-3’(Forward) and 5’-TGCACCATCTGTCACTCTGTTAACCTC-3’(Reverse) on a MiniAmp thermocycler (Applied Biosystems, USA).

**Adenoviral transduction**

Recombinant adenoviruses expressing the human wildtype and mutated 3′-UTRs of CP, SLC7A11 and GPX4 were produced by RiboBio (Guangzhou, China). All constructs were verified by sequencing. Cardiomyocytes were infected with 3′-UTRs of CP, SLC7A11 and GPX4 adenovirals and the Ad-CMV-null control vector at 10 infectious units (IFUs)/cell for 24 h in serum-free medium.

**Oligonucleotide, expression plasmid and shRNA transfection**

Negative control (miR-NC), miR-338-3p inhibitor and miR-338-3p mimic were synthesized by Genechem Co (Shanghai, China). Human METTL3-coding sequence (CDS) expression plasmid were generated by cloning the full-length open reading frame of homo sapiens METTL3 gene (NM_019852.4) into pEZ-M02 vector for transient expression. The shRNA-negative control and shRNA-RBMX, shRNA-Rab27a, shRNA-Rab27b, and shRNA-METTL3 and shRNA-DGCR8 were designed and ordered from RiboBio (RiboBio, Guangzhou). Transfections were performed by using the Lipofectamine^TM^ 3000 Transfection Reagent (L3000015, Invitrogen) according to the manufacturers' instructions. All oligonucleotides and shRNA sequences used in this study are summarized in Supplementary Table Ⅳ.

**Western blot**

The protocol of Western blot analysis was as previously described^28^. After respective treatment, cells were harvested and protein was extracted, protein concentrations from different samples were determined with the BCA protein assay kit. Proteins on the separation gel were transferred to PVDF membranes (BioRed Laboratories, USA) by wet transfer under 300 mA for 120 min. The PVDF membrane were blocked with 5% FBS in TBST (Tris-buffered saline solution with 0.1% Tween-20) for 2 h at room temperature and then incubated with 1:1000 TBST diluted primary antibodies. ImageJ was used to calculate the gray value of the band. All the primary and secondary antibodies used in this study are summarized in Supplementary Table Ⅲ.

**Human study of plasma sEVs**

The human study was approved by the Ethics Committee of the People's Liberation Army General Hospital (S2022-522-01). Five breast cancer patients suffered from doxorubicin induced cardiotoxicity and age and sex matched healthy controls were recruited from Chinese PLA general hospital from 2021-2023 (patient's characteristics were summarized in Supplementary Table Ⅱ). All study subjects were Chinese nationality. The diagnostic criteria for breast cancer were based on the 2012 World Health Organization (WHO) criteria. The diagnostic criteria for patients suffered from doxorubicin induced cardiotoxicity are as follows, (1) In the follow-up examination of DOX therapy, patients who develop new significant LV systolic dysfunction (decline of >10 points in LVEF to ≤40 percent or an absolute decline of ≤15 percentage points to an LVEF >40 percent and/or heart failure); (2) Patients with an asymptomatic decline of LVEF to <50 percent but >40 percent (https://www.uptodate.cn/contents/clinical-manifestations-diagnosis-and-treatment-of-anthracycline-induced-cardiotoxicity). Participants were excluded from the following conditions: (1) a history of myocarditis, dilated cardiomyopathy, congenital heart disease, coronary heart disease, and other primary cardiac diseases; (2) Patients with severe blood disorders, active liver disease, other malignant tumors, inflammatory processes, and infectious diseases in the past two months. All subjects signed the informed consent before being included in the study. These peripheral fasting blood samples were drawn *via* a freshly inserted 21-23G butterfly syringe into BD K2 Ethylenediamine tetra acetic acid (EDTA) lined tubes and processed within 2 h. Blood was centrifuged at 2,500 x g for 10 min at room temperature to remove cells and aliquot the resulting platelet-free supernatant (plasma). The plasma was snap-frozen at -80 °C until further usage. All procedures relating to human clinical samples were performed in accordance with the principles of the 1975 Declaration of Helsinki.

**sEVs isolation and characterization**

sEVs were isolated from murine blood, human blood and culture media in this study. Mouse blood was collected by intracardiac puncture into an Eppendorf tube (~1.5 ml) containing trisodium citrate as an anticoagulant and centrifuged at 1000×g for 5 minutes twice to obtain plasma within 2 hours. Fasting human blood was collected in EDTA anticoagulant tubes and centrifuged, to obtain plasma within 2 hours. To isolate sEVs for further study, the supernatant was filtered through a 0.22-µM filter into Ultra centrifuge tubes (U-tubes, 355631, Beckman Coulter). To eliminate larger extracellular vesicles, balanced U-tubes were centrifuged at 16,000g for 40 min, 4 °C, using the Optima XPN-80 Ultracentrifuge. The supernatant was then transferred into new U-tubes and smaller extracellular vesicles, including exosomes, were pelleted at 120,000g for 4 h at 4 °C. The sEV pellet was resuspended in PBS and stored at -80 °C. Isolation of sEVs from plasma is similar, except for the ultracentrifuge process, which involves 1.5-ml microcentrifuge tubes (REF357448, Beckman Coulter) spinning for 2 h at maximum speed (186,000g) in a tabletop Optima Ultracentrifuge. sEVs were observed by electron microscopy using negative staining, and quantified using a NanoSight NS300 instrument (Malvern Instruments) equipped with NTA 3.0 analytical software. Primary antibodies against CD63 (ab134045, Abcam, USA), CD9 (ab236630, Abcam, USA), Calnexin (ab133615, Abcam, USA), GM130 (ab52649, Abcam, USA) were used for the identification of sEVs by Western blot.

To deplete miRNA content in sEVs, Dicer was knocked out by CRISPR/Cas9 KO plasmids targeting DICER1 (Santa Cruz, sc-400365), a key regulator of miRNA biogenesis, in MDA-MB-231 cells. The CRISPR/Cas9 KO plasmid was transfected into 231 cells by Lipofectamine^TM^ 3000 Transfection Reagent (L3000015, Invitrogen). At 48 h post-transfection, puromycin selection was applied at a concentration of 1 mg/mL for 72 h. Then, the remaining cells were sorted as a single cell into a 96-well plate and cultured in regular medium for an additional 3 weeks. Dicer 1 deficient cells were genotyped by the PCR assay according to the manufacturer’s instructions.

For BCC-sEVs treatment in hiCMs or AMVCs, a total of 1×10^6^ hiCMs or AMVCs were treated with 20 µg of sEVs or an equal volume of medium in the presence or absence of DOX for 24 hours.

**Visualization of sEVs internalization**

To study the uptake of sEVs by cardiomyocytes, PKH26 or PKH63 fluorescent cell linker Kit (PKH26/63PCL, Sigma-Aldrich, Inc, USA) was used to label sEVs isolated from 231 cells. sEVs were then washed through exosome spin columns (MW3000, Invitrogen). Labelled sEVs were added to AMVCs or HiCMs and then visualized by a laser scanning confocal microscopy (LSM710, Zeiss). To further monitor cargo delivery between 231 cells and hiCMs, we employed a stable lentivector-based system to label sEVs with RFP by expressing the pCT-CD63-RFP Cyto-Tracer (CYTO120R-VA-1, SBI) in the parent 231 cells, 231 cells were then transfected by Cy5-miR-338-3p mimic and incubated with hiCMs, colocalization of Cy5 and RFP was observed in hiCMs recipient cells following incubation with 231/CD63-RFP + Cy5-miR-338-3p cells by a laser scanning confocal microscopy (LSM710, Zeiss).

**sEVs miRNA sequencing and data analysis**

miRNA library preparation and sequencing were conducted by a commercial service (Nuohe Biological Co., Ltd, China). Briefly, total RNAs were extracted from sEVs purified from 50 ml of culture medium. Both 3' and 5' adaptors were added to each end, respectively, followed by reverse transcription and PCR amplification. The PCR products derived from the 18- to 30- nucleotide RNA molecules were purified by electrophoresis and sequenced using the Illumina HiSeq 2500 platform. Differential expression of miRNAs between the 2 groups was analyzed using cluster analysis. For the data analysis, the criterion for significantly differential expression was set at p-value <0.05 and |log2(fold change) | > 1. miRNA sequencing has been deposited in the Gene Expression Omnibus (GEO) under accession code GSE192763.

**Real-time polymerase chain reaction (qPCR)**

Total RNAs from tissues, cells, and sEVs were extracted using an RNAfast 200 kit (Fastagen, 220011). sEVs-containing microRNAs were extracted using exoRNeasy Midi Kit (Qiagen, 77144). MicroRNA reverse transcription and qPCR analysis were performed using Taqman miRNA reverse transcription kit (Applied Biosystems, 4366596) and Taqman premix (Takara, RR390A), respectively. Total cDNA was synthesized using HiScript II Reverse Transcriptase (Vazyme, R201-02). The quantitative real-time-PCR reaction was performed by using SYBR Green Master Mix (A46109, Thermo Fisher Scientific, USA) and was performed with the ABI Prism 7500 System. Human endogenous U6 small RNA was used for the normalization of cellular miRNA. Considering that the U6 RNA may be unstable in the sEVs, the cel-miR-39 was set as the expression control. The PCR primers for miRNA and U6 were purchased from RiBoBio (Guangzhou, China). All primer sequences used in this study are summarized in Supplementary Table Ⅲ.

**RIP-qPCR**

RNA immunoprecipitation coupled with quantitative reverse transcription polymerase chain reaction (RIP-qPCR) were performed using EZ-Magna RIP Kit (Millipore) following the manufacturer's instructions. Briefly, cells were harvested and lysed in a buffer containing an RNA-processing enzyme (RNase) inhibitor and protease inhibitor cocktail. The cell lysates (100 μL from 2 × 10^7^ cells per RIP reaction) were processed for the RIP assay using primary antibodies against m^6^A (ab208577, Abcam), DGCR8 (ab18757, Abcam), RBMX (#14794, Cell Signaling Technology), or nonspecific IgG (negative control) (ab171870, Abcam, USA). The antibody-bound protein/RNA complexes were then recovered using magnetic protein A/G beads (Millipore). Finally, coprecipitated RNA was purified and reverse transcribed using the standard protocol described above followed by quantitative analysis by qPCR. The primers used in RIP-qPCR are summarized in Supplementary Table Ⅲ.

**Pull down assay with biotinylated DNA probes**

RNA pulldown assays were performed as previously described^33^. GenePharma provided the 3′-end biotinylated miR-338-3p and its negative control (NC). After transfection with biotinylated miR-338-3p or its NC, 231 cells were harvested 48 hours later. The cells were then collected and treated for 10 minutes on ice in lysis buffer [20 mmol/L Tris, pH 7.5, 200 mmol/L NaCl, 2.5 mmol/L MgCl2, 0.05% IGEPAL, 60 U/mL Superase-In (Ambion), 1 mmol/L dithiothreitol, protease inhibitors (Roche)]. Small aliquots (50 L) of the samples were utilized as input after the lysates were precleared by centrifugation. The leftover lysates were then pelleted after being treated with streptavidin-Sepharose beads (Sigma) and were washed five times. The resultant beads were then loaded onto SDS-PAGE gels after being incubated in SDS-PAGE loading buffer. Western blot was then employed to examine the expression of RBMX in pull down products. The oligonucleotide sequences were shown in Supplementary Table Ⅲ.

**Detection of Cardiac Troponin I level**

The level of Cardiac Troponin I release in cell supernatant was detected using a Cardiac Troponin I (TNNI3) ELISA Kit (EHTNNI3, Thermo Fisher Scientific, USA) as per the manufacturer's instructions. Absorbance was then measured at 490 nm.

**Cell viability assay**

We used the Cell Counting Kit-8 assay kit (C0037; Beyotime, China) to determine cell viability as per the manufacturer's instructions. Microplate readers were used to measuring 450 nm absorbance.

**Cell death staining**

Cells were grown on coverslips and treated as described. Paraformaldehyde at a final concentration of 4% was added to the medium of cultured cardiomyocytes, fixed for 20 min, permeabilized and stained with 1 μM SYTOX Nucleic Acid Stain (Thermo Fisher Scientific, USA) for 5 min, excess dye was washed away with PBS. Subsequently, cells were permeabilized with 0.1% Triton X-100 in PBS for 5 min, blocked with PBS containing 5%FBS for 30 min, stained with primary antibody against α-actinin (A7811, Sigma-Aldrich, Inc, USA) for 1 h, and stained with secondary antibodies for 1 h. Cell nuclei were then labelled by 200 ng/mL 40,6-diamidino-2-phenylindole (DAPI) (ThermoFisher Scientific, 62247) for 2 min, and the image was captured using an inverted fluorescence microscope (Olympus, Tokyo, Japan). The SYTOX/ DAPI positive ratio is calculated from ten randomly selected fields.

**Luciferase reporter assay**

AC16 cells were co-transfected with 160 ng psiCHECK-2-CP 3'UTR/ mutant or psiCHECK-2 SLC7A11 3'UTR/mutant or psiCHECK-2- GPX4 3'UTR/ mutant and 50 nmol/L miR-338-3p mimic or negative control (Ribobio, China) using Lipofectamine-3000 (Invitrogen) following the manufacturer's instructions. Cells were incubated for 6 hours with the transfection complex before it was discarded and replaced by fresh media. After 48 hours, cells were harvested, and firefly and renilla luciferase activities were analyzed with the Dual-Luciferase Reporter Assay System (Promega. USA) according to the manufacturer's instruction. Luciferase activity was normalized by the Renilla/Firefly luciferase signal in 293T cells.

**Electrophoretic mobility shift assays (EMSA)**

NE-PER™ Nuclear and Cytoplasmic Extraction Reagents (78835, Thermo Fisher Scientific, USA) were used to prepare nuclear extracts. DNA probes containing the RBMX binding site of the miR-338-3p (Supplementary Table Ⅲ) were labeled with biotin at the 3' end using Pierce™ Biotin 3' End DNA Labeling Kit (89818, Thermo Fisher Scientific, USA), according to manufacturer’s instructions. The DNA binding reaction was performed using a LightShift™ Chemiluminescent EMSA Kit (20148, Thermo Fisher Scientific, USA). A 50-fold molar excess was added simultaneously with unlabeled oligonucleotides as competitor-labeled probes. To identify DNA-binding proteins, nuclear extracts were incubated with anti-RBMX antibody or nonspecific anti-IgG antibody (negative control) 20 minutes before adding a labeled probe.

**m^6^A dot blot**

Poly (A) RNA was purified from total cellular RNA using the PolyATtract® mRNA Isolation System (Promega, USA). RNA samples (250 ng, 500 ng) dissolved in 1× saline sodium citrate (SSC) (0.15 mol/L NaCl, 0.015 mol/L sodium citrate; Sigma-Aldrich) were then transferred onto an Amersham Hybond-N+ membrane (Amersham Biosciences) with a Bio-Dot apparatus (Bio-Rad, Hercules, CA, USA), followed by ultraviolet cross-linking for 5 min. Then, to ensure consistency among different groups, the membrane was stained with 0.02% methylene blue (Sangon Biotech, Shanghai, China) in 0.3 mol/L sodium acetate (pH 5.2). The membrane was then blocked with 5% nonfat milk in PBS containing 0.6% Triton-X 100 (PBST) and incubated with m^6^A antibody (Abcam) overnight at 4°C. Finally, after incubation with horseradish peroxidase (HRP)-conjugated goat anti-rabbit immunoglobulin G (IgG) (1:3000 dilution; Proteintech, USA), the membrane was visualized using the chemiluminescence method (Amersham Biosciences).

**Single-base elongation-and ligation-based qPCR amplification (SELECT) assay**

The m^6^A modification site was analyzed using the SELECT method according to a previous report^34^. In brief, total RNA with the appropriate treatment was recovered by phenol-chloroform extraction and ethanol precipitation. Then, RNA was mixed with up primers, down primers, and dNTPs in 1× CutSmart buffer (50 mmol/L KAc, 20 mmol/L Tris-HAc, 10 mmol/L MgAc2, and 100 μg/mL BSA, pH 7.9). After annealing, a mixture containing Bst 2.0 DNA polymerase (NEB, M0537S), 0.5 U SplintR ligase (NEB, M0375S), and 10 nmol ATP (NEB, P0756S) was added for the reaction, followed by qRT-PCR analysis. Primer sequences are listed in Supplementary Table Ⅲ.

**Ferroptosis associated assays**

Liperfluo probe for lipid peroxides detection

Liperfluo (L248, Dojindo, Japan) was utilized to specifically detect the fluorescent imaging of lipid peroxides in living cells at a concentration of 10 μM. Upon lipid oxidation in the cells, Liperfluo undergoes specific oxidation by lipid peroxides and emits intense fluorescence. Fluorescence was detected with a laser confocal fluorescence microscope (Excitation: 488 nm, Emission: 500-550 nm). Fluorescence intensities of 10-15 randomly selected field were averaged and analyzed using FlowJo Software.

Evaluation of malondialdehyde (MDA)

To detect the changes of malondialdehyde (MDA), a key indicator of lipid peroxidation in cell lysates and homogenization of cardiac tissues, MDA Assay Kit (MAK085, Sigma-Aldrich, Inc, USA) was used to assess MDA concentrations according to the manufacturer's instructions.

Evaluation of iron concentration

Treated HiCM (2 × 10^6^) or cardiac tissues were rapidly homogenized in iron assay buffer with iron assay kit (sigma Aldrich, cat number: MAK025) as previously described^35^. Briefly, iron (total iron, ferrous iron, and ferric iron) is released by the addition of an acidic buffer. Released iron reacted with the iron probe resulting in a colorimetric (593 nm) product, proportional to the iron present. Then the solution was centrifuged at 13,000g for 10 min at 4°C to remove insoluble material and was measured at 593 nm with a microplate fluorometer.

Glutathione measurement

Glutathione level measurement was performed as per the manufacturer’s instructions. After respective treatment, cell lysates or cardiac tissue homogenates were prepared for measurement of glutathione using the Glutathione Assay Kit (#703002, Cayman Chemical) according to the manufacturer’s protocol. The GSH and GSSG concentrations were calculated using a standard curve and normalized to the total protein level.

Fe^2+^ fluorescence detection

The level of intracellular ferrous ion was determined using FerroOrange dye (#F374, Dojindo Laboratories). Cells were seeded and exposed to the indicated treatments. After treatment, FerroOrange (1 μM) was added to the cells and the cells were incubated at 37°C for 30 min. Fluorescence was detected with a laser confocal fluorescence microscope (excitation wavelength: 532 nm, emission wavelength: 572 nm). Fluorescence intensities of 10-15 randomly selected field were averaged and analyzed using FlowJo Software.

**Supplementary Table Ⅰ. Upregulated and downregulated miRNAs in D-BCC-sEVs**

**revealed by miRNA sequencing.**

| **miRNA information** | | | | | |  |
| --- | --- | --- | --- | --- | --- | --- |
| **Mature miRNA** | **pre-mature** | **log2FoldChange** | **pvalue** | **padj** | **Regulation** | |
| hsa-miR-206 | hsa-mir-206 | 6.101338 | 0.000458 | 0.032289 | up | |
| hsa-miR-338-3p | hsa-mir-338 | 5.973128 | 3.57E-06 | 0.00088 | up | |
| hsa-miR-34c-5p | hsa-mir-34c | 5.653405 | 0.006087 | 0.269538 | up | |
| hsa-miR-145-5p | hsa-mir-145 | 4.998883 | 0.037175 | 0.663843 | up | |
| hsa-miR-505-5p | hsa-mir-505 | 4.678509 | 0.045427 | 0.722432 | up | |
| hsa-miR-1298-5p | hsa-mir-1298 | 3.992603 | 0.003643 | 0.179921 | up | |
| hsa-miR-654-5p | hsa-mir-654 | 3.603348 | 0.03905 | 0.663843 | up | |
| hsa-miR-30e-5p | hsa-mir-30e | 2.496881 | 0.035605 | 0.663843 | up | |
| hsa-miR-128-3p | hsa-mir-128-1 | 1.604706 | 0.000309 | 0.025375 | up | |
| hsa-miR-424-3p | hsa-mir-424 | 1.11436 | 0.030865 | 0.663843 | up | |

**Downregulated sEVs miRNAs**

| **miRNA information** | | | | | |  |
| --- | --- | --- | --- | --- | --- | --- |
| **Mature miRNA** | **pre-mature** | **log2FoldChange** | **pvalue** | **padj** | **Regulation** | |
| hsa-miR-106b-3p | hsa-mir-106b | -1.10106 | 0.008783 | 0.309294 | down | |
| hsa-miR-28-3p | hsa-mir-28 | -1.10091 | 0.033203 | 0.663843 | down | |

**Supplementary Table Ⅱ. Characteristics of healthy donor and cancer patients (without past history of heart disease, Chinese ethnicity) suffered from DOXIC.**

| **Subject** | **Diagnosis** | **Age(years)** | **Gender** | **LVEF (%)** | **Cumulative dose of DOX** |
| --- | --- | --- | --- | --- | --- |
| 1 | healthy donor | 41 | female | 72 | - |
| 2 | healthy donor | 39 | female | 69 | - |
| 3 | healthy donor | 55 | female | 65 | - |
| 4 | healthy donor | 33 | female | 70 | - |
| 5 | healthy donor | 41 | female | 67 | - |
| 6 | breast cancer | 42 | female | 64 | 350 mg/m^2^ |
| 7 | breast cancer | 37 | female | 51 | 250 mg/m^2^ |
| 8 | breast cancer | 53 | female | 46 | 280 mg/m^2^ |
| 9 | breast cancer | 33 | female | 40 | 300 mg/m^2^ |
| 10 | breast cancer | 42 | female | 38 | 320 mg/m^2^ |

**Supplementary Table Ⅲ. qPCR (Quantitative PCR) primer and other nucleotide sequences used in this study (* F: forward; R: reverse).**

| 1. Primers for qPCR | |
| --- | --- |
| Name | **Sequences (5'-3')** |
| hsa-miR-206-F | GCAGTGGAATGTAAGGAAGT |
| hsa-miR-206-R | CCAGTTTTTTTTTTTTTTTCCACACA |
| hsa-miR-338-3p-F | GCAGTCCAGCATCAGTG |
| hsa-miR-338-3p-R | GGTCCAGTTTTTTTTTTTTTTTCAAC |
| hsa-miR-34c-5p-F | GCAGAGGCAGTGTAGTTAG |
| hsa-miR-34c-5p-R | CCAGTTTTTTTTTTTTTTTGCAATCAG |
| hsa-miR-145-5p-F | GTCCAGTTTTCCCAGGAATC |
| hsa-miR-145-5p-R | AGGTCCAGTTTTTTTTTTTTTTTAGG |
| hsa-miR-505-5p-F | GGGGAGCCAGGAAGT |
| hsa-miR-505-5p-R | GGTCCAGTTTTTTTTTTTTTTTACATCA |
| hsa-miR-1298-5p-F | GTTCATTCGGCTGTCCAG |
| hsa-miR-1298-5p-R | GGTCCAGTTTTTTTTTTTTTTTACATC |
| hsa-miR-654-5p-F | TGGGCCGCAGAACA |
| hsa-miR-654-5p-R | GGTCCAGTTTTTTTTTTTTTTTGCA |
| hsa-miR-30e-5p-F | CGCAGTGTAAACATCCTTGAC |
| hsa-miR-30e-5p-R | CCAGTTTTTTTTTTTTTTTCTTCCAG |
| hsa-miR-128-3p-F | CACAGTGAACCGGTCTC |
| hsa-miR-128-3p-R | CAGGTCCAGTTTTTTTTTTTTTTTAAAG |
| hsa-miR-424-3p-F | CGCAGCAAAACGTGAGG |
| hsa-miR-424-3p-R | GGTCCAGTTTTTTTTTTTTTTTATAGCA |
| hsa-pri-miR-338-F | GAAGAAGTGGCGAAGGACAC |
| hsa-pri-miR-338-R | TGCCCTCTTCAACAAAATCA |
| Cel-miR-39-F | GTCACCGGGTGTAAATCAG |
| Cel-miR-39-R | GGTCCAGTTTTTTTTTTTTTTTCAAG |
| U6-F | CGCTTCGGCAGCACATATAC |
| U6-R | AAAATATGGAACGCTTCACGA |
| mmu-miR-338-3p-F | GCAGTCCAGCATCAGTG |
| mmu-miR-338-3p-R | GGTCCAGTTTTTTTTTTTTTTTCAAC |
| GPX4-F | GAGGCAAGACCGAAGTAAACTAC |
| GPX4-R | CCGAACTGGTTACACGGGAA |
| SLC39A14-F | AAGGCCCTACTCAACCACCT |
| SLC39A14-R | CGACTGCTCGCTGAAATTGTG |
| ATG7-F | ATGATCCCTGTAACTTAGCCCA |
| ATG7-R | CACGGAAGCAAACAACTTCAAC |
| SLC7A11-F | TCTCCAAAGGAGGTTACCTGC |
| SLC7A11-R | AGACTCCCCTCAGTAAAGTGAC |
| AKR1C1-F | TTCATGCCTGTCCTGGGATTT |
| AKR1C1-R | CTGGCTTTACAGACACTGGAAAA |
| ACSL4-F | CATCCCTGGAGCAGATACTCT |
| ACSL4-R | TCACTTAGGATTTCCCTGGTCC |
| TFRC-F | ACCATTGTCATATACCCGGTTCA |
| TFRC-R | CAATAGCCCAAGTAGCCAATCAT |
| IREB2-F | TCGATGTATCTAAACTTGGCACC |
| IREB2-R | GCCATCACAATTTCGTACAGCAG |
| PRNP-F | AGTCAGTGGAACAAGCCGAG |
| PRNP-R | CTGCCGAAATGTATGATGGGC |
| HMGCR-F | TGATTGACCTTTCCAGAGCAAG |
| HMGCR-R | CTAAAATTGCCATTCCACGAGC |
| STEAP3-F | CTCCCCGGAGGTCATCTTTG |
| STEAP3-R | TCTTGCTCTGTAGGGTTGCTC |
| SLC1A5-F | GAGCTGCTTATCCGCTTCTTC |
| SLC1A5-R | GGGGCGTACCACATGATCC |
| AKR1C2-F | GACAGGCATGAAGTGACCATC |
| AKR1C2-R | GAAGAAACATTTGCTAACCAGG |
| ATG5-F | AAAGATGTGCTTCGAGATGTGT |
| ATG5-R | CACTTTGTCAGTTACCAACGTCA |
| CP-F | GGGCCATCTACCCTGATAACA |
| CP-R | TTAAAGGTCCGATGAGTCCTGA |
| METTL3-F | TTGTCTCCAACCTTCCGTAGT |
| METTL3-R | CCAGATCAGAGAGGTGGTGTAG |
| RAB27A-F | GCTTTGGGAGACTCTGGTGTA |
| RAB27A-R | TCAATGCCCACTGTTGTGATAAA |
| RAB27B-F | TAGACTTTCGGGAAAAACGTGTG |
| RAB27B-R | AGAAGCTCTGTTGACTGGTGA |
| DGCR8-F | GCAGAGGTAATGGACGTTGG |
| DGCR8-R | AGAGAAGCTCCGTAGAAGTTGAA |
| GAPDH-F | TCCACCACCCTGTTGCTGTAG |
| GAPDH-R | ACCCACTCCTCCACCTTTGAC |
| 1. miRNA inhibitor and mimics sequences (5'-3') | |
| hsa-miR-34c-5p mimic | AGGCAGUGUAGUUAGCUGAUUGC |
| hsa-miR-145-5p mimic | GTCCAGTTTTCCCAGGAATCCCT |
| hsa-miR-206 mimic | TGGAATGTAAGGAAGTGTGTGG |
| hsa-miR-1298-5p mimic | TTCATTCGGCTGTCCAGATGTA |
| hsa-miR-338-3p mimic | TCCAGCATCAGTGATTTTGTTG |
| hsa-miR-338-3p inhibitor | CAACAAAATCACTGATGCTGGA |
| Negative control mimic | TTTGTACTACACAAAAGTACTG |
| 1. ShRNA and its negative control (NC) sequences (5'-3') | |
| shMETTL3 | GCAAGTATGTTCACTATGAAATTCAAGAGAATAATTCGTCTGAAGTGCAGC-TTTTTT |
| ShRBMX | ACATGGATGACGGTGGATATTTTCAAGAGA- AATATCCACCGTCATCCATGT-TTTTT |
| shNC | GCGCTTGTCTAAGATCAAATTTTCAAGAGA- AATTTGATCTTAGACAAGCGC-TTTTT |
| shDGCR8 | GGGGTGAGAGTGCTGATAATTTTCAAGA GATTATCAGCACTCTCACCCCTTTTT |
| ShACO1 | GGATGTTTAAGGAAGTCTATTCAAGAGATAGACTTCCTTAAACATCCTTTTT |
| ShELAVL1 | GGTGAAGTTGAATCTGCAATTCAAGAGAGGTGAAGTTGAATCTGCAATTTTT |
| 1. Biotin-coupled probe pull down assay probe sequences (5'-3') | |
| miR-338-3p | Biotin-CAACAAAATCACTGATGCTGGA |
| miR-338-3p-mut | Biotin-CAACAAAATCACTGATGTACCA |
| 1. Electrophoretic mobility shift assay probe sequences (5'-3') | |
| miR-338-3p | TCCAGCATCAGTGATTTTGTTG-Biotin |
| miR-338-3p mut1 | TCCATCATCAGTGATTTTGTTG-Biotin |
| miR-338-3p mut2 | TGGTACATCAGTGATTTTGTTG-Biotin |

1. **SELECT m^6^A qPCR primer sequences used in this study (5'-3')**

| M6A 414-F | AGCCCCTGGGGACCCCGGGG |
| --- | --- |
| M6A 414-R | TCGCCACTTCTTCCAGCA |
| M6A 438-F | GCTGGAAGAAGTGGCGAA |
| M6A 438-R | CTGGGGCTGACGGGGCCACG |
| M6A 635-F | CCACTCCACCCCCTAGGCCCA |
| M6A 635-R | GGCCCTGTAGCGATCCCCTGGCA |
| M6A 969-F | CAGGGCTGGACCCTAAAACT |
| M6A 969-R | GGGGTCCTGCCGGCTGCCGG |
| M6A 996-F | GGCAGCCGGCAGGACCCCTG |
| M6A 996-R | TGGGGAAACTGAGGCCTGGAG |

1. **RIP-qPCR primer sequences used in this study**

| DGCR8 RIP-F | GCCAGGATGCCCAGCCCCTGG |
| --- | --- |
| DGCR8 RIP-R | CCACATAAAACCCATGTCTGCG |
| MeRIP-F | CAGCCCCTGGGGACCCCGGGG |
| MeRIP-R | TGCCAGGGGATCGCTACAGGGCC |

1. **The sequence of each single strand of DNA**

| ssDNA S1 | GAGCGTTAGCCACACACACAGTC |
| --- | --- |
| ssDNA S2 | TTAGGCGAGTGTGGCAGAGGTGT |
| ssDNA S3 | CGCCTAAACAAGTGGAGACTGTG |
| ssDNA S4 | AACGCTCACCACTTGAACACCTC |

**Supplementary Table Ⅳ. Antibodies and reagents used in this study.**

| **Name** | **Company** | **Catalog No.** |
| --- | --- | --- |
| Doxorubicin | Beyotime | SC0159 |
| Ferrostatin-1 | Sigma Aldrich | SML0583 |
| Liproxstatin-1 | Selleck Chemicals | S7699 |
| Necrosulfonamide | Selleck Chemicals | S8251 |
| Z-VAD-FMK | Sigma Aldrich | V116 |
| VX765 | Selleck Chemicals | S2228 |
| Hydroxychloroquine | Selleck Chemicals | E4824 |
| STM2457 | Selleck Chemicals | S9870 |
| GW4869* | Selleck Chemicals | S7609 |
| Anti-CD63 | Abcam | ab134045 |
| Anti-CD9 | Abcam | ab236630 |
| Anti-Calnexin | Abcam | ab133615 |
| Anti-GM130 | Abcam | ab52649 |
| Anti-β-actin | Abcam | ab8226 |
| Anti-DGCR8 | Abcam | ab18757 |
| Anti-RBMX | Cell Signaling Technology | 14794 |
| Anti-TfR1 | Abcam | ab214039 |
| Anti-COX2 | Abcam | ab283574 |
| Anti-GPX4 | Abcam | ab125066 |
| anti-SLC7A11 | Abcam | ab307601 |
| Anti-CP | Abcam | ab157452 |
| anti-IgG | Abcam | ab171879 |
| Anti-Cardiac Troponin I | Abcam | ab47003 |
| Anti-Cardiac Troponin T | Abcam | ab45932 |
| Anti-Rabbit IgG H&L (HRP) | Cell Signaling Technology | 7074 |
| Anti-Mouse IgG H&L (HRP) | Cell Signaling Technology | 7076 |
| anti-Mouse IgG (H+L), Alexa Fluor 594 | Thermofisher | A-21203 |
| Anti-Rabbit IgG (H+L), Alexa Fluor 488 | Thermofisher | A-21206 |
| Anti-Rabbit IgG (H+L), Alexa Fluor 647 | Thermofisher | A-31573 |
| Mouse IgG Isotype Control | Thermofisher | 31903 |
| Rabbit IgG Isotype Control | Thermofisher | 31235 |

*The GW4869 powder was dissolved in DMSO to a stock concentration of 1 mM and then diluted with medium to a 10 μM working solution.

**Supplementary FIG. 1**

**
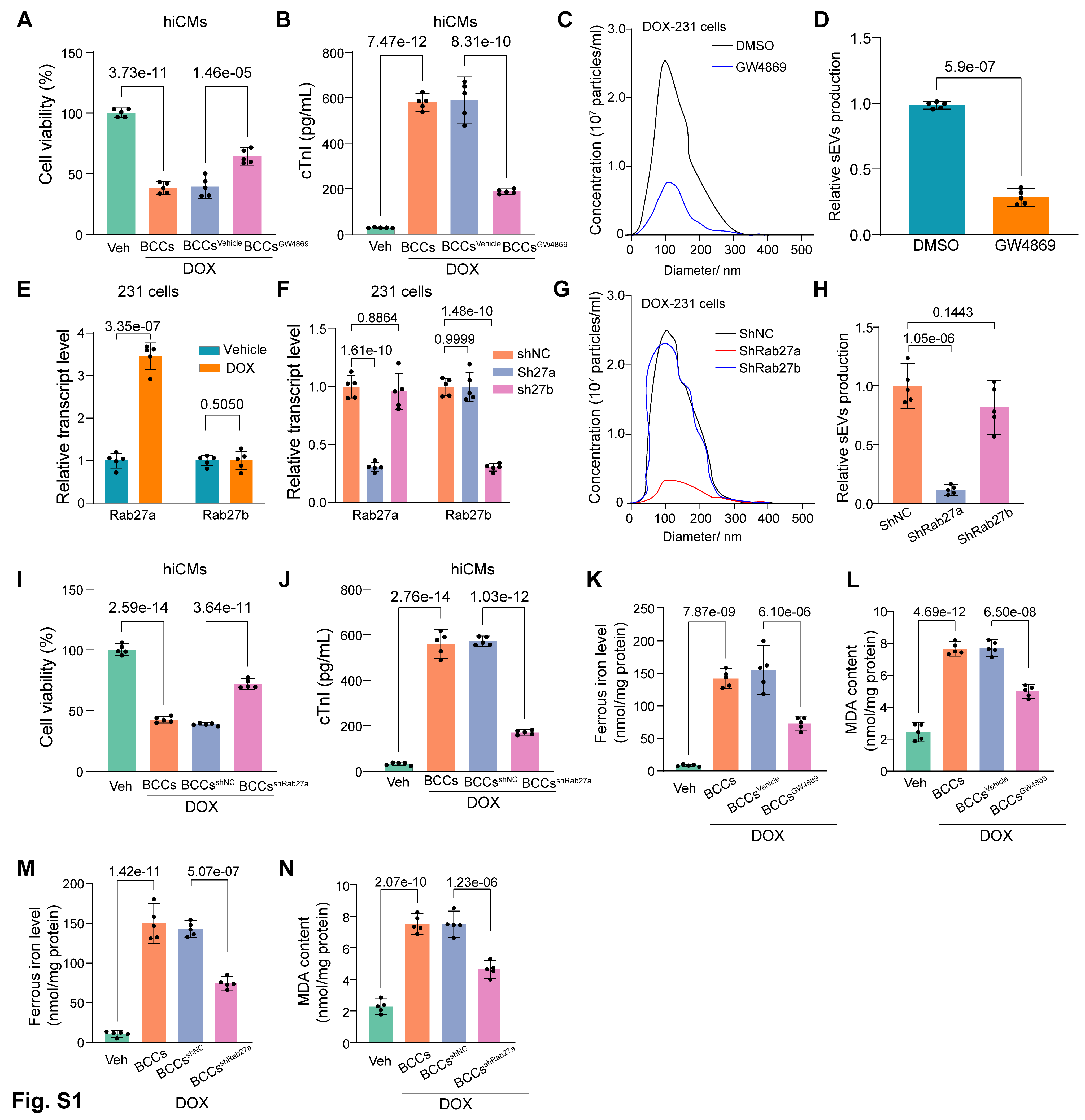
**

**Supplementary FIG. 1. Small extracellular vesicles (sEVs) mediated the pathological communications between BCCs and cardiomyocytes in DOXIC.** sEVs were depleted from 231 cells by pretreating cells with sEVs secretion inhibitor GW4869 (10 μM, 48 h) before coculture. GW4869 or vehicle pretreated-231 cells were cocultured with hiCMs in a transwell system and challenged with 1 μM DOX for 24 hours, cell viability (**A**) and cTnI leakage (**B**) were measured in hiCMs (n=5); (**C, D**) The nanosight tracking analysis (NTA) was used to determine particle concentrations of isolated sEVs in 231 cells upon GW4869 treatment (n=5); (**E**) qPCR analysis of Rab27a and Rab27b mRNA level in DOX-treated 231 cells (n=5); (**F**) qPCR analysis of Rab27a and Rab27b mRNA level upon shRNA transfection (n=5); (**G, H**) The nanosight tracking analysis (NTA) was used to determine particle concentrations of isolated sEVs in 231 cells upon shRNA transfection (n=5); (**I, J**) Negative ShRNA (ShNC) or ShRNA targeting Rab27a (ShRab27a)-transfected 231 cells were cocultured with hiCMs in a transwell system and challenged with 1 μM DOX for 24 hours, cell viability (**I**) and cTnI leakage (**J**) were measured in hiCMs (n=5); (**K, L**) Ferrous iron level (**K**) and MDA concentration (**L**) in the cell lysates of hiCMs following the treatment mentioned in A were quantified by respective assay kit (n=5); (**M, N**) Ferrous iron level (**M**) and MDA concentration (**N**) in the cell lysates of hiCMs following the treatment mentioned in F were quantified by respective assay kit (n=5). All data were collected from at least 3 independent experiments. Unless otherwise indicated, data were analyzed by 1-way ANOVA, followed by Bonferroni post hoc test.

**Supplementary FIG. 2**

**
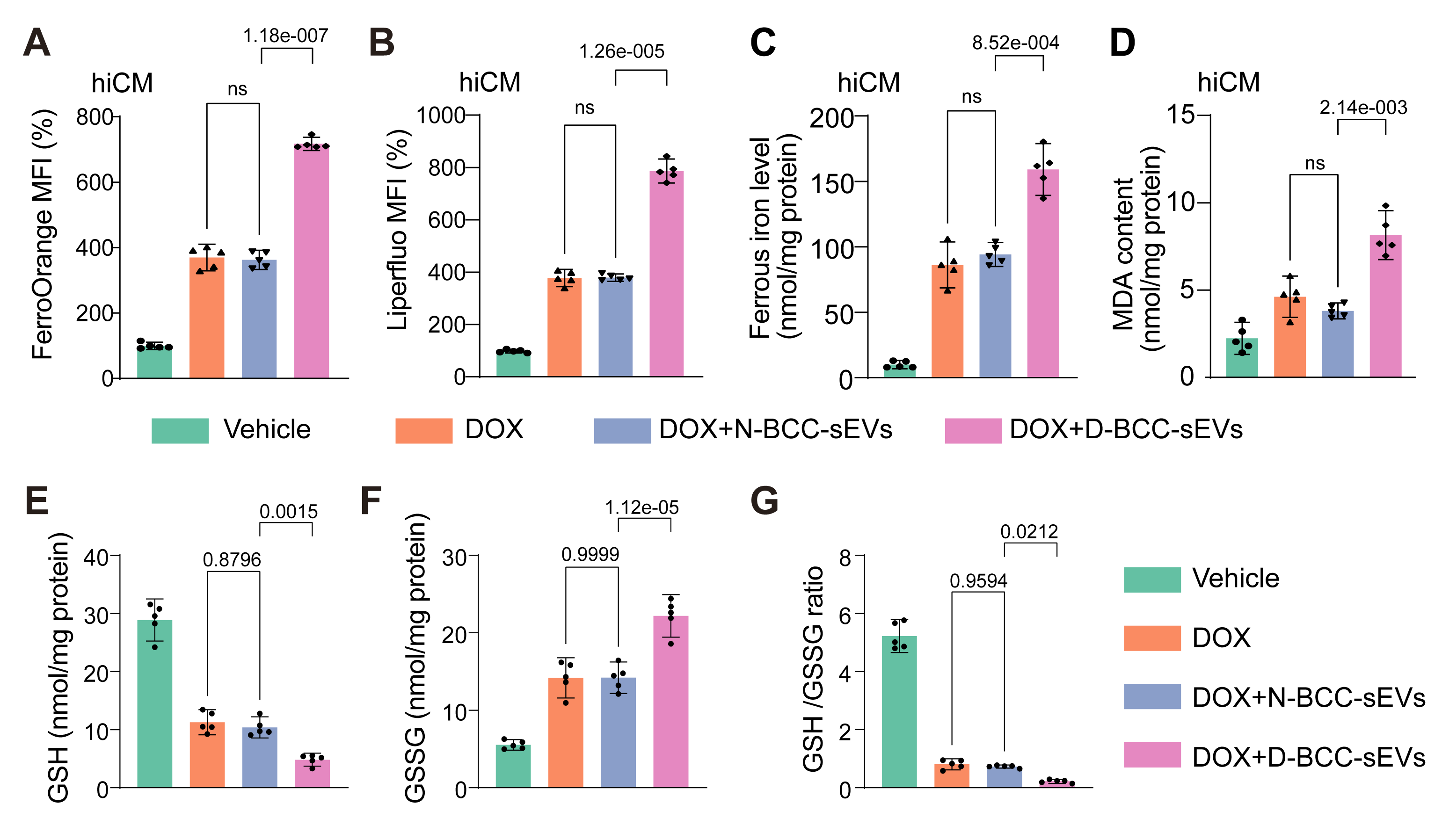
**

**Supplementary FIG. 2. Small extracellular vesicles (sEVs) from DOX-treated breast cancer cells** **aggravated DOX-induced cardiomyocytes ferroptosis. (A)**  Quantitative analysis of FerroOrange fluorescent intensities denoting intracellular ferrous iron deposition in hiCMs after 1μM DOX treatment for 24 h in the presence or absence of N-BCC-sEVs and D-BCC-sEVs; **(B)** Quantitative analysis of Liperfluo fluorescent intensities denoting lipid peroxidation levels in hiCMs following treatment mentioned in A. (**C, D**) Ferrous iron level (**J**) and MDA concentration (**K**) in the cell lysates of hiCMs following the treatment mentioned in A were quantified by respective assay kit (n=5). (**E-G**) glutathione (GSH) (**E**), oxidized state glutathione disulfide (GSSG) (**F**) and GSH/GSSG (**G**) in the cell lysates of hiCMs following the treatment mentioned in A were quantified by assay kit (n=5); All data were collected from at least 3 independent experiments. Unless otherwise indicated, data were analyzed by 1-way ANOVA, followed by Bonferroni *post hoc* test.

**Supplementary FIG. 3**

**
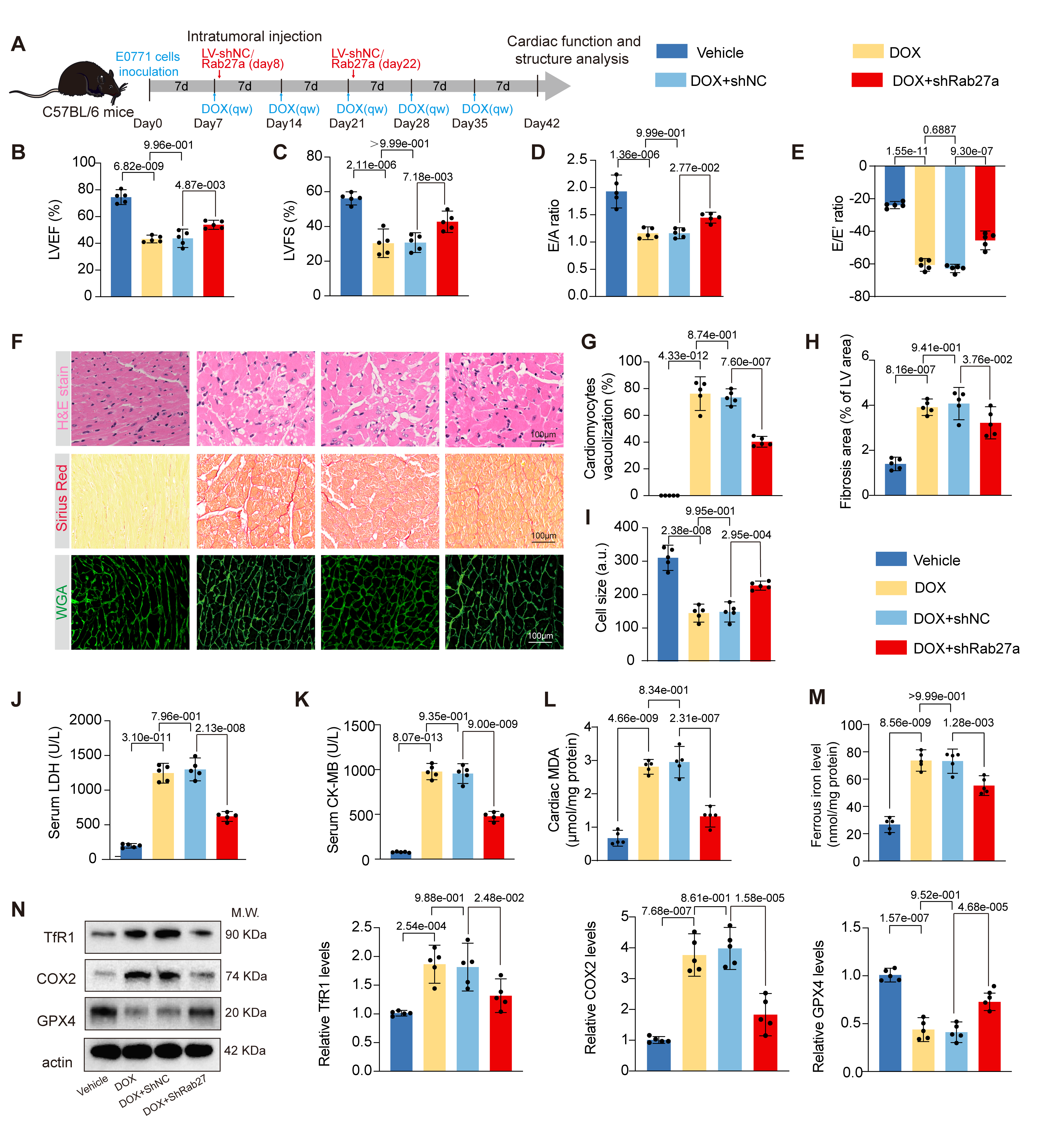
Supplementary FIG. 3. Blockage of sEVs release from breast cancer tissue (BCT) mitigated doxorubicin toxicity to heart in an orthotopic breast cancer mouse model.** (**A**) Schematic illustration showing DOX exposure and intratumoral injection of lentiviral shRNA targeting Rab27a (LV-ShRab27a) in orthotopic breast cancer mice; (**B**) Echocardiographic analysis of left ventricular ejection fraction (LVEF) (**B**), left ventricular fraction shortening (LVFS) (**C**), E/A ratios (**D**), and E/E’ ratio (**E**) in DOX exposure and LV-ShRab27a-treated mice (n=5); (**F**) Representative images of hematoxylin and eosin staining to visualize myocardial histological changes (upper), Sirius red staining (middle) to indicate myocardial fibrosis, and wheat germ agglutinin (WGA) immunofluorescence staining (lower) to evaluate myocardial atrophy; (**G**) The statistics of cardiac vacuolization ratio in ventricular tissues (n=5); (**H**) The fibrotic area per left ventricle was quantified based on Sirius red staining (n=5); (**I**) Cell size was quantified based on WGA staining (n=5); Serum levels of Lactate dehydrogenase (LDH) (**J**) and Creatine kinase-MB (CK-MB) (**K**) were detected by colorimetric method (n=5); MDA concentration (**L**) and ferrous iron level (**M**) in the homogenization of cardiac tissues was quantified by respective assay kit (n=5); (**N**) The protein levels of TfR1, COX2, and GPX4 were detected and quantified by Western blot in murine ventricular tissues (n=5); All data were collected from at least 3 independent experiments. Unless otherwise indicated, data were analyzed by 1-way ANOVA, followed by Bonferroni *post hoc* test.

**Supplementary FIG. 4**

**
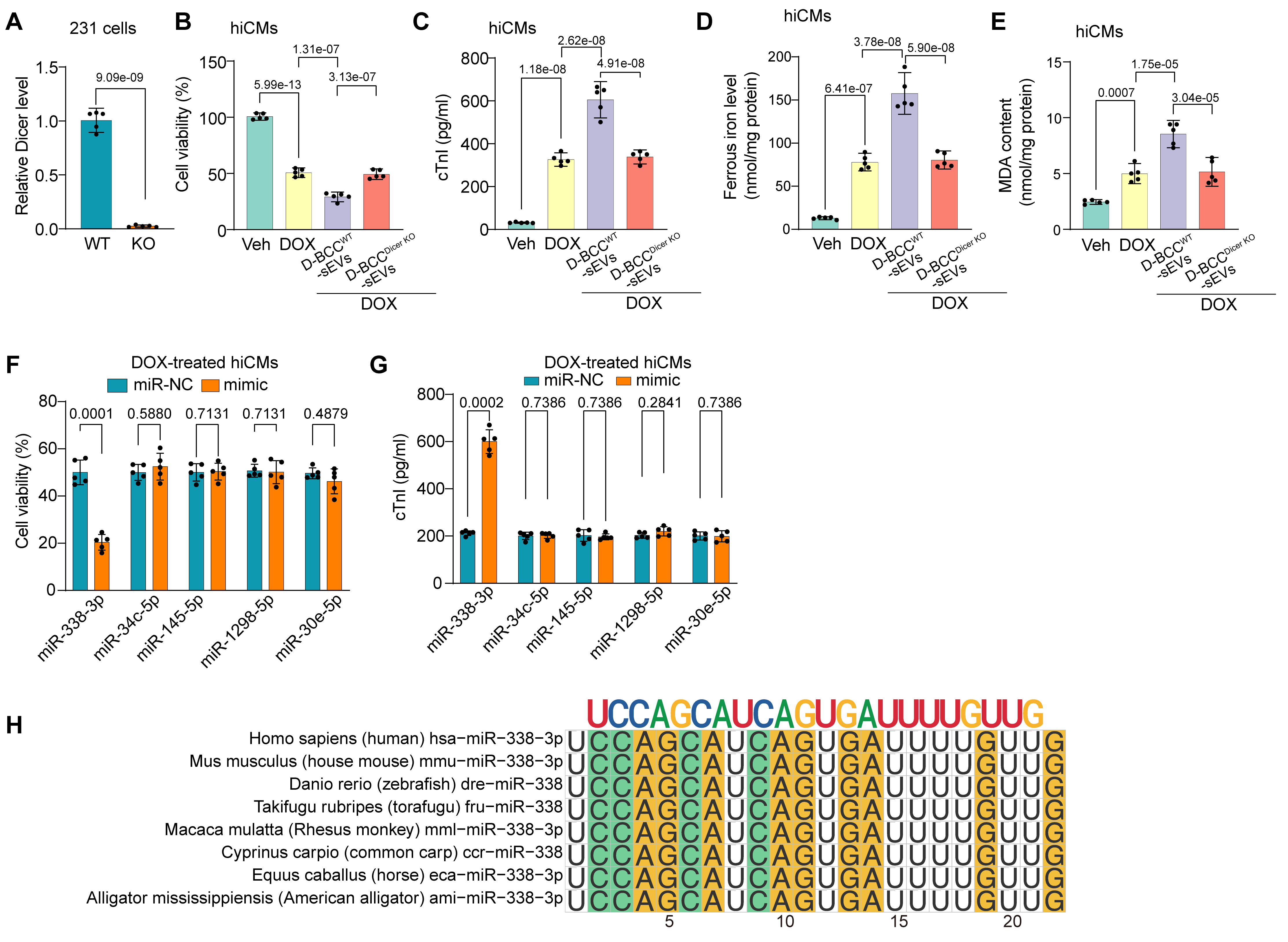
Supplementary FIG. 4. miRNA is necessary for D-BCC-sEVs to aggravate DOX-induced hiCMs injury. (A)** qPCR analysis of Dicer mRNA level in CRISPR-Cas9 mediated Dicer KO 231 cells (n=5); (**B**) D-BCC-sEVs were isolated from wildtype or Dicer KO 231 cells (BCC^WT^ and BCC^Dicer KO^, respectively), their effects on DOX-induced cell viability (**B**), cTnI leakage (**C**), Ferrous iron level (**D**) and MDA concentration (**E**) in hiCMs were examined (n=5); (**F, G**) hiCMs were transfected with the mimics of 5 candidate miRNAs separately and exposed to DOX. cell viability (**F**), and cTnI leakage (**G**) in hiCMs were examined (n=5); (**H**) The sequence conservation of miR-338-3p among mammals was shown. All data were collected from at least 3 independent experiments. Unless otherwise indicated, data were analyzed by 1-way ANOVA, followed by Bonferroni post hoc test.

**Supplementary FIG. 5**

**
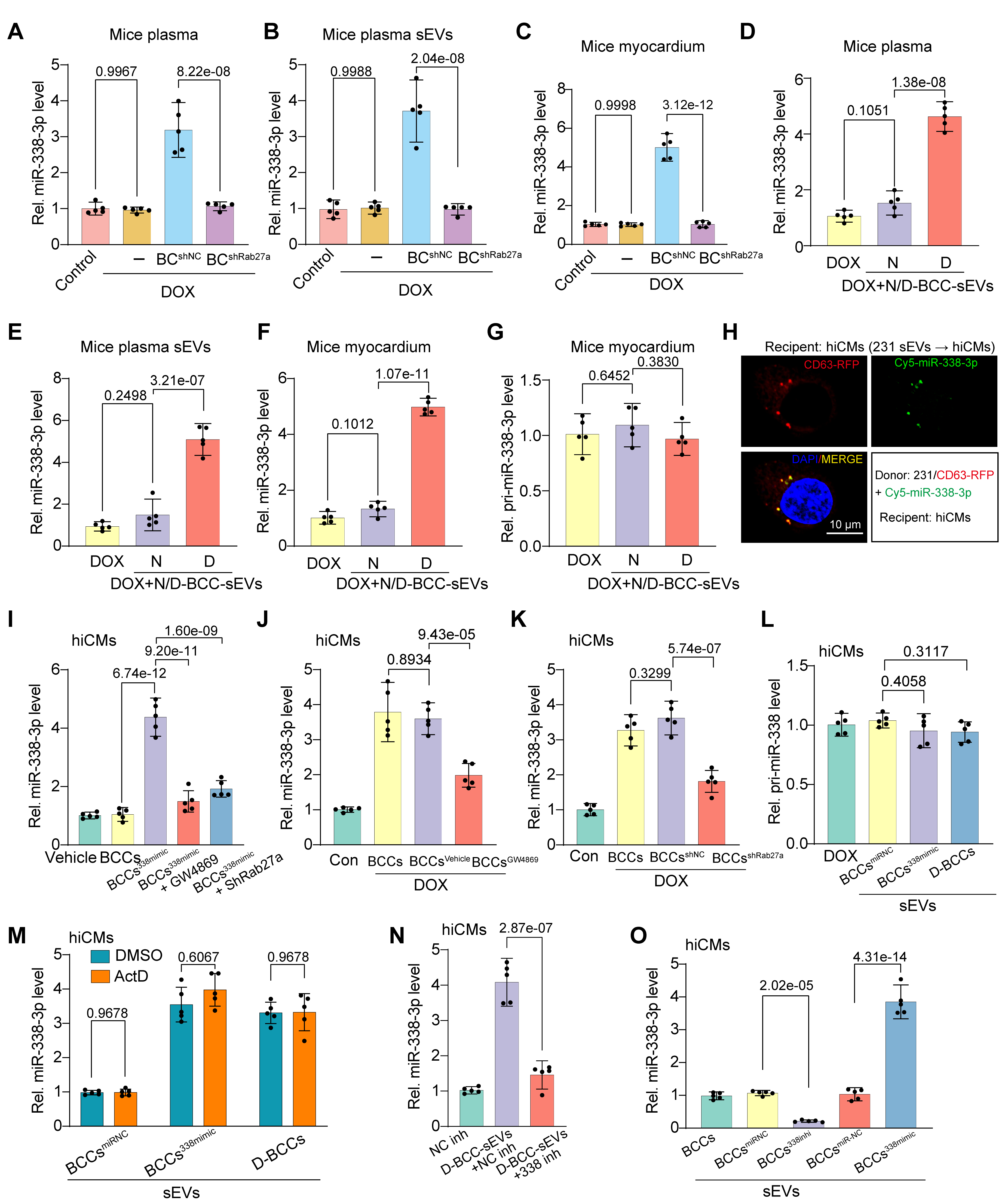
Supplementary FIG. 5.** **The increased cardiac miR-338-3p is of breast cancer origin and transmitted to recipient heart/cardiomyocytes in a sEV-dependent manner.** **(A-C)** Non-tumor bearing or tumor bearing (orthotopic breast cancer) mice treated with DOX were subjected to intratumoral injection of lentiviral shRNA targeting Rab27a or negative control ShRNA (ShNC), qPCR analysis of miR-338-3p levels in plasma **(A)**, plasma sEVs **(B),** and cardiac tissues **(C)** was shown (n=5); (**D-F**) In non-tumor bearing mice with DOX exposure and tail-vein injection of sEVs from vehicle and DOX-treated E0771 breast cancer cells (N-BCC-sEVs and D-BCC-sEVs, respectively), qPCR analysis of miR-338-3p levels in plasma **(D)**, plasma sEVs **(E),** and cardiac tissues **(F)** was shown (n=5); (**G**) qPCR analysis of pri-miR-338 level in cardiac tissues of mice following the treatment mentioned in D (n=5); (**H**) 231 cells were transfected with Cy5-miR-338-3p-mimic and transduced with lentiviral RFP-CD63 and used them as donor cells. Colocalization of Cy5 and RFP was observed in hiCMs recipient cells following incubation with co-transfected 231 cells; (**I**) 231 cells were transfected with the exogenously synthesized miR-338-3p mimic in the presence or absence of ShRab27a cotransfection or GW4869 (10 μM, 48 h) pretreatment, and subsequently incubated with hiCMs using a transwell system. qPCR analysis of miR-338-3p levels in hiCMs were evaluated (n=5); (**J**) sEVs were depleted from 231 cells by pretreating cells with sEVs secretion inhibitor GW4869 (10 μM, 48 h) before coculture. GW4869 or vehicle pretreated-231 cells were cocultured with hiCMs in a transwell system and challenged with 1 μM DOX for 24 hours, qPCR analysis of miR-338-3p levels in hiCMs were evaluated (n=5); (**K**) Negative ShRNA (ShNC) or ShRNA targeting Rab27a (ShRab27a)-transfected 231 cells were cocultured with hiCMs in a transwell system and challenged with 1 μM DOX for 24 hours, qPCR analysis of miR-338-3p levels in hiCMs were evaluated (n=5); (**L**) D-BCC-sEVs or sEVs isolated from miR-338-3p-overexpressed 231 cells (BCC^338mimic^-sEVs) incubation failed to increase the primary miR-338-3p (pri-miR-338) in hiCMs (n=5); (**M**) The increased miR-338-3p levels in recipient hiCMs following the incubation with sEVs isolated miR-338-3p-mimic transfected 231 cells (BCC338^mimic^) or DOX-treated 231 cells (D-BCCs) were not affected by the RNA polymerase II inhibitor, actinomycin (1 μg/mL, n=5); (**N**) miR-338-3p inhibitor successfully blocked the expression of transmitted miR-338-3p in hiCMs induced by D-BCC-sEVs (n=5); (**O**) sEVs were isolated from Dicer-KO 231 cells that depleted all endogenous miRNAs and loaded with exogenous miR-338-3p mimic and inhibitor to exclude the potential involvement of other miRNAs. The efficacies of these sEVs in modulating miR-338-3p in recipient hiCMs were validated (n=5); All data were collected from at least 3 independent experiments. Unless otherwise indicated, data were analyzed by 1-way ANOVA, followed by Bonferroni post hoc test.

**Supplementary FIG. 6**


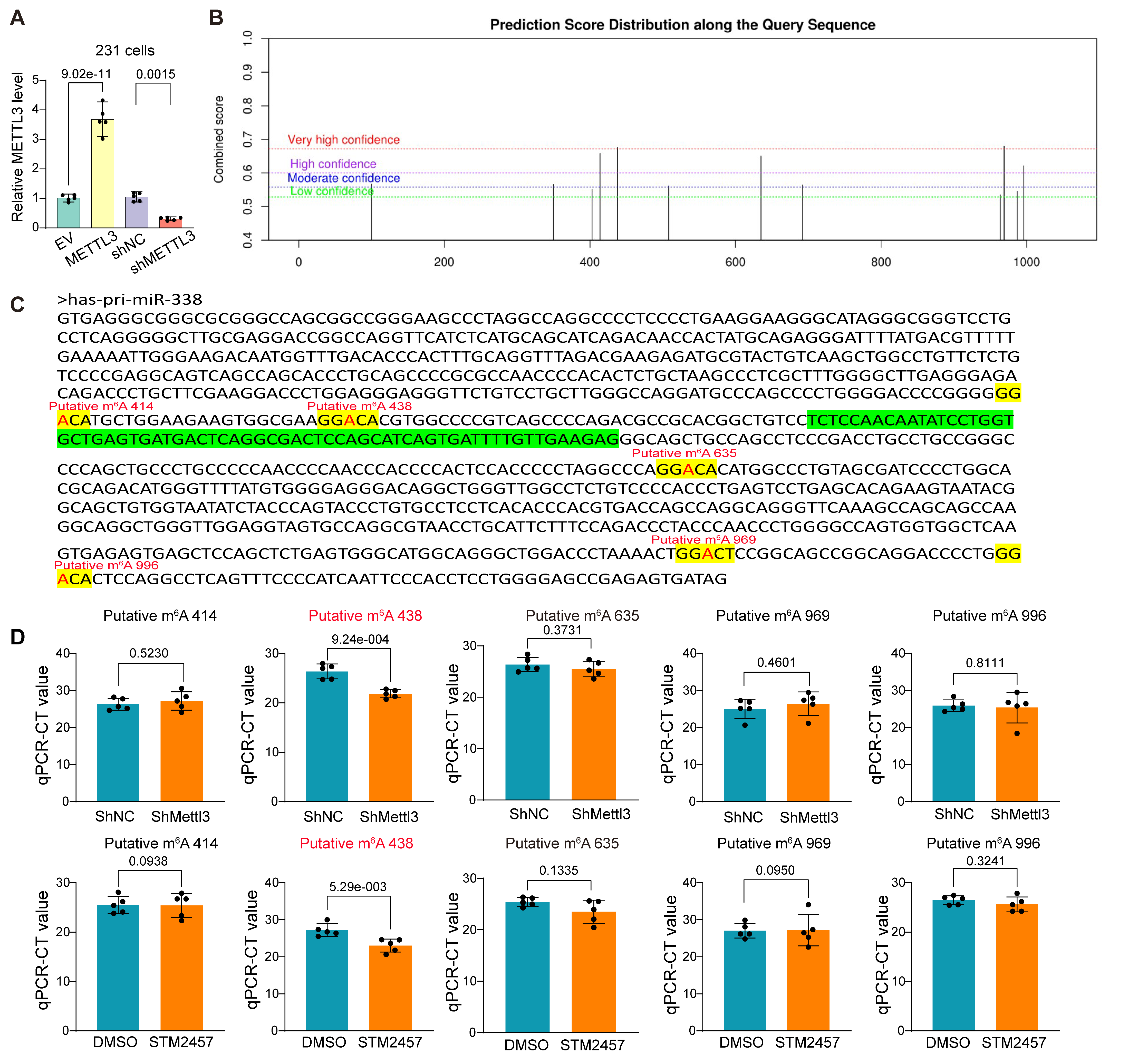


**Supplementary FIG. 6. Bioinformatic analysis predicted the potential m^6^A methylation sites resided in the sequence of pri-miR-338 and its experimental validation.** **(A)** METTL3 was manipulated in 231 cells by transfecting 231 cells with plasmids that overexpress METTL3 or ShRNA targeting METTL3 (ShMETTL3) to knockdown METTL3, the expression of METTL3 mRNA was evaluated by qPCR (n=5); **(B, C)** Primary miR-338 sequence was analyzed and several consensus m^6^A motifs (RRACH) within the pri-miR-338 sequence were identified using a sequence-based N^6^-methyladenosine (m^6^A) modification site predictor; (**D**) The predicted m^6^A sites were validated by a single-base elongation- and ligation-based quantitative PCR amplification method (called SELECT) in DOX-induced 231 cells. Only the qPCR cycle threshold (Ct) values of m^6^A 438 decreased upon METTL3 silence and STM2457 treatment (n=5). All data were collected from at least 3 independent experiments. The data in Supplementary FIG. 6A were analyzed by 1-way ANOVA, followed by Bonferroni post hoc test. The data in Supplementary FIG. 6D were analyzed by unpaired 2-tailed Student t test and multiple comparisons P values were corrected by Holm-Sidak method.

**Supplementary FIG. 7**

**
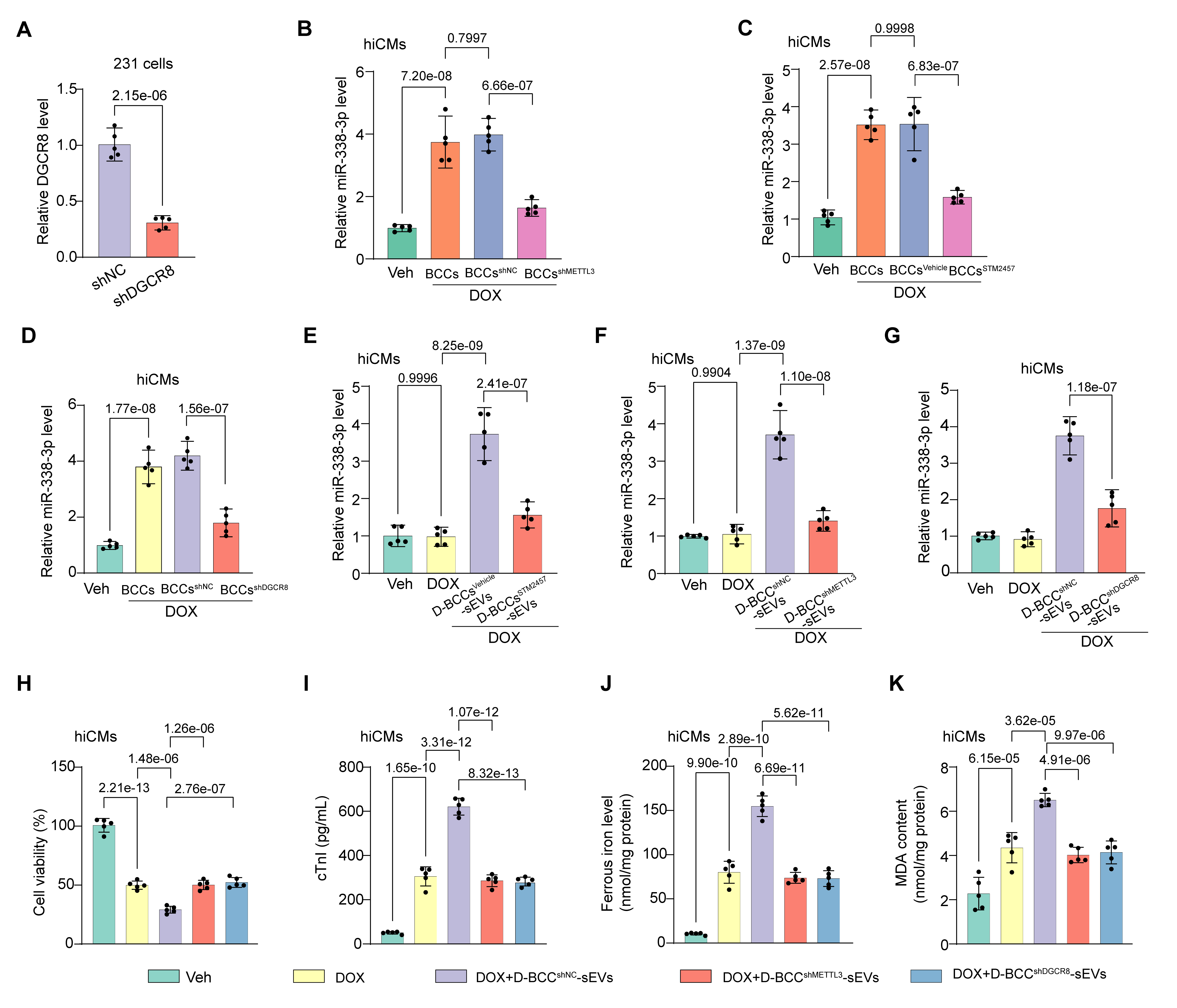
Supplementary FIG. 7. Blockage of the m^6^A-mediated primary pri-miR-338 maturation in BCCs suppressed sEVs-transmitted miR-338-3p and D-BCC-sEVs-induced DOXIC aggravating effects. (A)** qPCR analysis of DGCR8 mRNA level in 231 cells transfected with ShRNA targeting DGCR8 (ShDGCR8) to knockdown DGCR8 (n=5); The data in Supplementary FIG. 7A were analyzed by unpaired 2-tailed Student t test; **(B)** Negative control ShRNA (ShNC) or ShRNA targeting METTL3 (ShMETTL3)-transfected 231 cells were cocultured with hiCMs in a transwell system and challenged with 1 μM DOX for 24 hours, miR-338-3p level was examined in hiCMs (n=5); **(C)** STM2457 (10 μM, 24 hours) or vehicle pretreated-231 cells were cocultured with hiCMs in a transwell system and challenged with 1 μM DOX for 24 hours, miR-338-3p level was examined in hiCMs (n=5); (**D**) Negative ShRNA (ShNC) or ShRNA targeting DGCR8 (ShDGCR8)-transfected 231 cells were cocultured with hiCMs in a transwell system and challenged with 1 μM DOX for 24 hours, miR-338-3p level was examined in hiCMs (n=5); (**E**) D-BCC-sEVs were isolated from vehicle or STM2457 pretreated (10 μM, 24 hours; BCC^vehicle^ and BCC^STM2457^, respectively), their effects on DOX-induced miR-338-3p level was examined in hiCMs (n=5); (**F**) D-BCC-sEVs were isolated from negative control shRNA (ShNC) or shRNA targeting METTL3 (ShMETTL3) transfected 231 cells (BCC^ShNC^ and BCC^ShMETTL3^, respectively), their effects on DOX-induced miR-338-3p level was examined in hiCMs (n=5); (**F**) D-BCC-sEVs were isolated from negative control shRNA (ShNC) or shRNA targeting DGCR8 (ShDGCR8) transfected 231 cells (BCC^ShNC^ and BCC^ShDGCR8^, respectively), their effects on DOX-induced miR-338-3p level was examined in hiCMs (n=5); (**H-K**) D-BCC-sEVs were isolated from negative control shRNA (ShNC) or shRNA targeting METTL3 (ShMETTL3) or shRNA targeting DGCR8 (ShDGCR8) transfected 231 cells (BCC^ShNC^, BCC^ShMETTL3^, and BCC^ShDGCR8^, respectively), their effects on DOX-induced cell viability (**H**), cTnI leakage (**I**), Ferrous iron level (**J**) and MDA concentration (**K**) in hiCMs were examined (n=5); All data were collected from at least 3 independent experiments. Unless otherwise indicated, data were analyzed by 1-way ANOVA, followed by Bonferroni post hoc test.

**Supplementary FIG. 8**

**
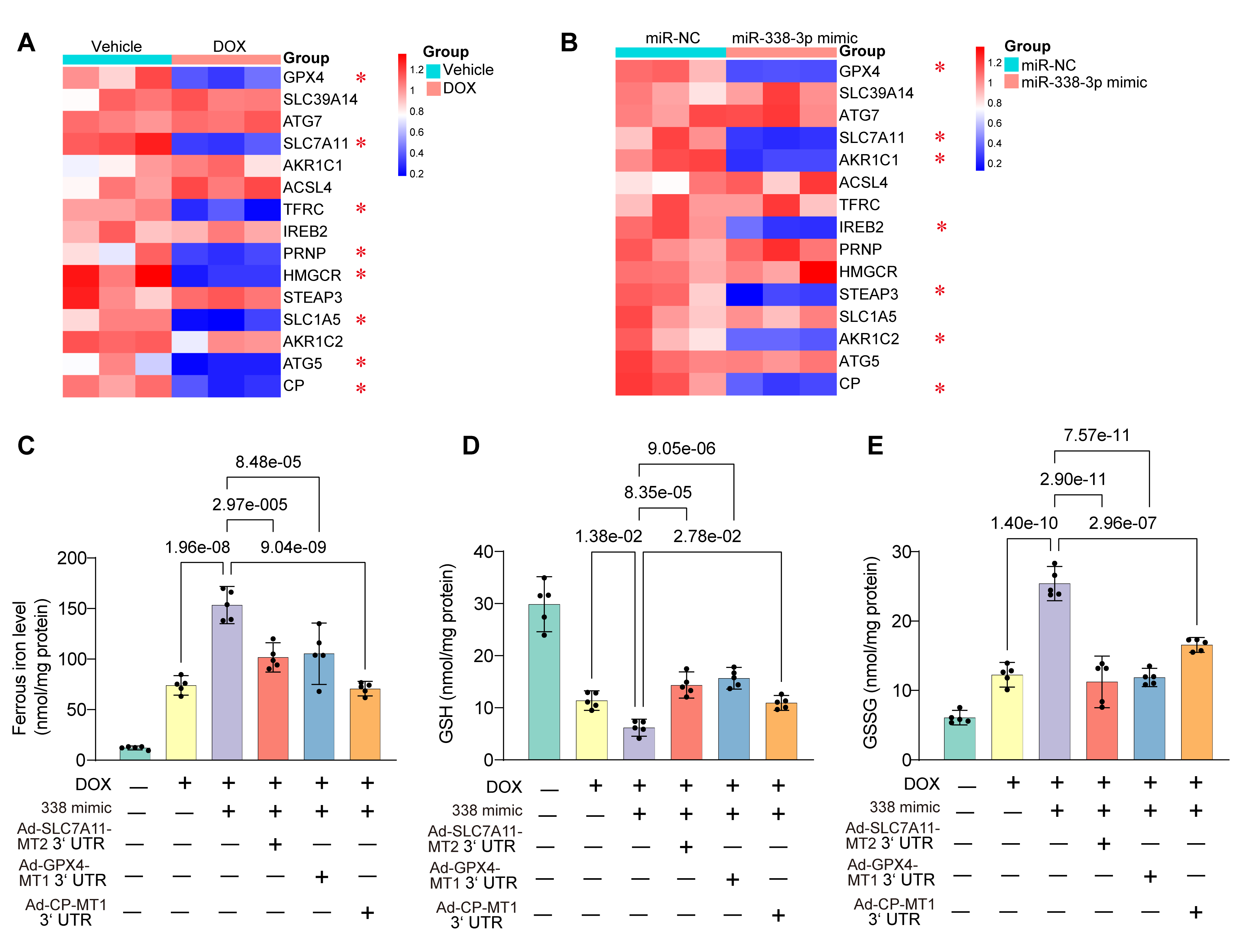
**

**Supplementary FIG. 8. miR-338-3p aggravated DOX-induced cardiomyocytes injury and ferroptosis through the interaction of miR-338-3p with CP, SLC7A11, and GPX4 3′-UTRs. (A)** hiCMs were transfected with miR-338-3p mimic. Heatmap depicting the mRNA changes of the 15 putative targets examined by qPCR. **(B)** hiCMs were exposed to 1μM DOX for 24 hrs. Heatmap depicting the mRNA changes of the 15 putative targets examined by qPCR. (**C-E**) in miR-338-3p-overexpressed hiCMs post DOX treatment (1 μM, 24 hours) with co-expression of mutated CP, SLC7A11, and GPX4 3′-untranslated regions (UTRs), Ferrous iron level (**C**), glutathione (GSH) (**D**), oxidized state glutathione disulfide (GSSG) (**E**) in the cell lysates of hiCMs were quantified by respective assay kit (n=5). All data were collected from at least 3 independent experiments. Unless otherwise indicated, data were analyzed by 1-way ANOVA, followed by Bonferroni post hoc test.

**Supplementary FIG. 9**

**
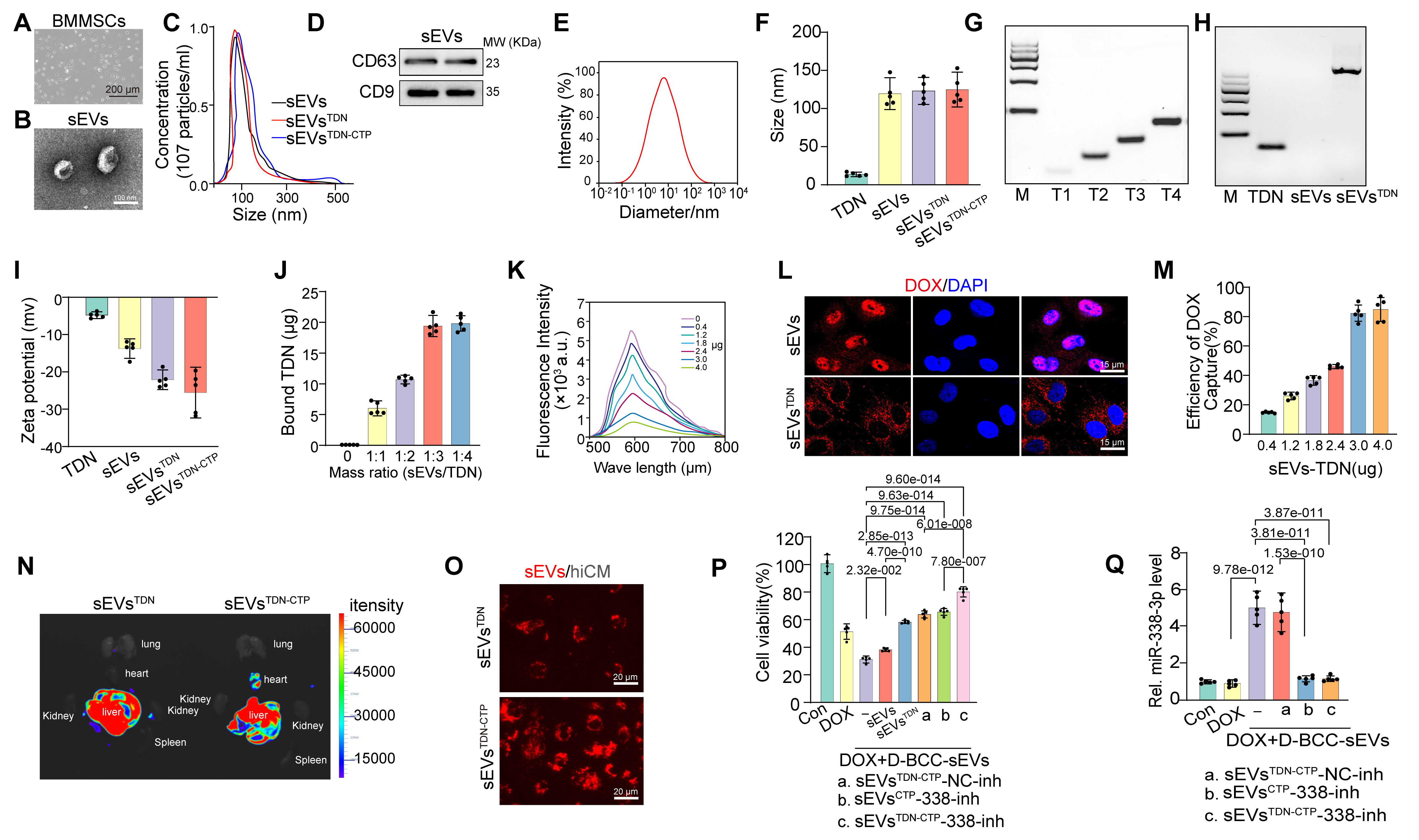
Supplementary FIG. 9. Characterization of dual-functional decoying sEVs encapsulated with miR-338-3p inhibitor.** (**A**) Representative brightfield image of third (P3) passage of bone marrow derived mesenchymal stem cells (BMSCs); (**B**) Representative TEM image of sEVs isolated from BMSCs; (**C**) The nanosight tracking analysis (NTA) was used to determine particle size distributions of isolated sEVs; (**D**) Immunoblot of isolated sEVs probed for sEV markers; (**E**) Dynamic light scattering (DLS) showed that the particle size of the TDN was around 10 nm; (**F**) Comparisons of particle size distributions of TDN and isolated sEVs (n=5); (**G**) Agarose electrophoresis of TDN (T1: S1; T2: S1+S2; T3: T2+S3; T4: T3+S4); (**H**) Agarose electrophoresis analysis of the synthesis of sEVs-TDN; (**I**) Zeta potential analysis of TDN, sEVs, and sEVs-TDN; (**J**) The grafting efficiency of TDN on the sEVs (n=5); (**K**) The fluorescence spectrophotometer of 1 μM DOX solution after adding different amounts of sEVs-TDN; (**L**) Confocal imaging of the inhibition of DOX from entering the nucleus of hiCMs cells by sEVs^TDN^; (**M**) The capture efficiency of DOX by sEVs^TDN^ (n=5); (**N**) *Ex vivo* fluorescence imaging to detect the biodistribution of sEVs^TDN and^ sEVs^TDN-CTP^; (**O**) PKH67 labelled sEVs^TDN^ and sEVs^TDN-CTP^ were incubated with hiCMs for 6 hours and the enrichment of sEVs^TDN-CTP^ in the cytoplasm of hiCMs was observed under microscopy; (**P**) The effects of various therapeutic sEVs on D-BCC-sEVs aggravated DOX-induced cell viability loss in hiCMs (n=5); (**Q**) The effects of various therapeutic sEVs on D-BCC-sEVs induced miR-338-30 accumulation in hiCMs (n=5). All data were collected from at least 3 independent experiments. Unless otherwise indicated, data were analyzed by 1-way ANOVA, followed by Bonferroni post hoc test.

**Supplementary FIG. 10**

**
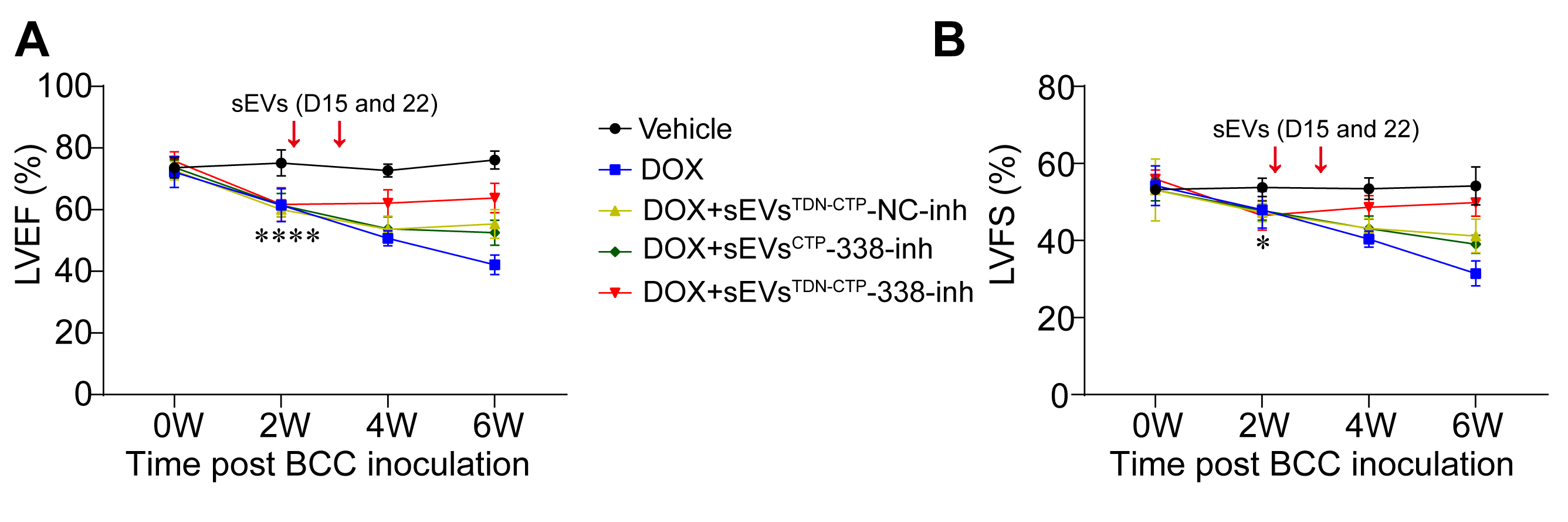
**

**Supplementary FIG. 10. A serial echocardiography examination to reveal cardiac function measured in mice receiving various therapeutic sEVs.** Echocardiographic analysis of dynamic changes in left ventricular ejection fraction (LVEF) (**A**), left ventricular fraction shortening (LVFS) (**B**) in DOX-exposured orthotopic breast cancer mice receiving various therapeutic sEVs (n=5). All data were collected from at least 3 independent experiments. Unless otherwise indicated, data were analyzed by 1-way ANOVA, followed by Bonferroni post hoc test.

**Supplementary FIG. 11**

**
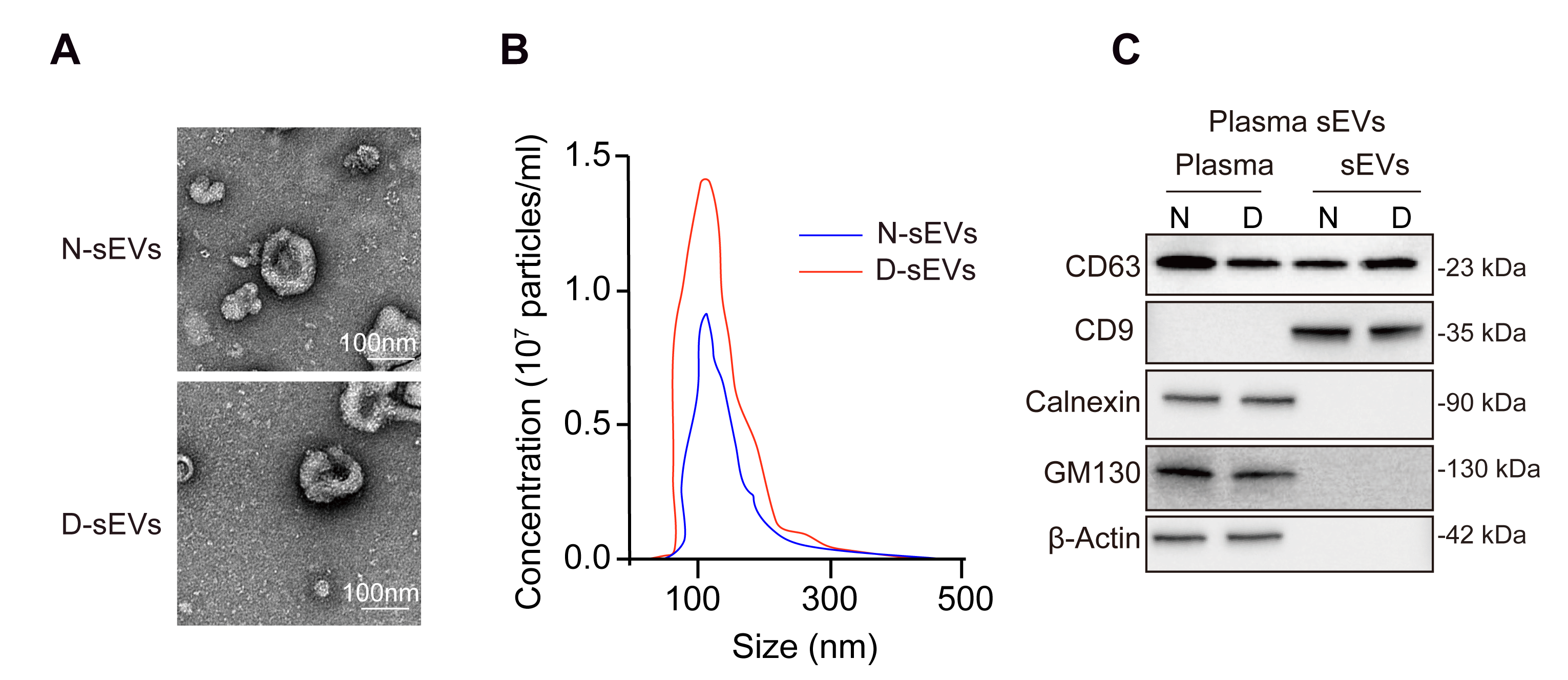
**

**Supplementary FIG. 11. Isolation and identification of human plasma EVs. (A)** Transmission electron microscopy (TEM) was used to characterize sEVs from healthy control and patients diagnosed as DOX-induced cardiomyopathy (N-sEVs and D-sEVs, respectively); **(B)** The nanosight tracking analysis (NTA) was used to determine particle size distributions of isolated sEVs; (**C**) Immunoblot of N-sEVs and D-sEVs probed for positive and negative sEV markers. All data were collected from at least 3 independent experiments.
